# Meta-Analysis of Genome-Wide Association Studies in African Americans Provides Insights into the Genetic Architecture of Type 2 Diabetes

**DOI:** 10.1371/journal.pgen.1004517

**Published:** 2014-08-07

**Authors:** Maggie C. Y. Ng, Daniel Shriner, Brian H. Chen, Jiang Li, Wei-Min Chen, Xiuqing Guo, Jiankang Liu, Suzette J. Bielinski, Lisa R. Yanek, Michael A. Nalls, Mary E. Comeau, Laura J. Rasmussen-Torvik, Richard A. Jensen, Daniel S. Evans, Yan V. Sun, Ping An, Sanjay R. Patel, Yingchang Lu, Jirong Long, Loren L. Armstrong, Lynne Wagenknecht, Lingyao Yang, Beverly M. Snively, Nicholette D. Palmer, Poorva Mudgal, Carl D. Langefeld, Keith L. Keene, Barry I. Freedman, Josyf C. Mychaleckyj, Uma Nayak, Leslie J. Raffel, Mark O. Goodarzi, Y-D Ida Chen, Herman A. Taylor, Adolfo Correa, Mario Sims, David Couper, James S. Pankow, Eric Boerwinkle, Adebowale Adeyemo, Ayo Doumatey, Guanjie Chen, Rasika A. Mathias, Dhananjay Vaidya, Andrew B. Singleton, Alan B. Zonderman, Robert P. Igo, John R. Sedor, the FIND Consortium, Edmond K. Kabagambe, David S. Siscovick, Barbara McKnight, Kenneth Rice, Yongmei Liu, Wen-Chi Hsueh, Wei Zhao, Lawrence F. Bielak, Aldi Kraja, Michael A. Province, Erwin P. Bottinger, Omri Gottesman, Qiuyin Cai, Wei Zheng, William J. Blot, William L. Lowe, Jennifer A. Pacheco, Dana C. Crawford, the eMERGE Consortium, the DIAGRAM Consortium, Elin Grundberg, the MuTHER Consortium, Stephen S. Rich, M. Geoffrey Hayes, Xiao-Ou Shu, Ruth J. F. Loos, Ingrid B. Borecki, Patricia A. Peyser, Steven R. Cummings, Bruce M. Psaty, Myriam Fornage, Sudha K. Iyengar, Michele K. Evans, Diane M. Becker, W. H. Linda Kao, James G. Wilson, Jerome I. Rotter, Michèle M. Sale, Simin Liu, Charles N. Rotimi, Donald W. Bowden, for the MEta-analysis of type 2 DIabetes in African Americans (MEDIA) Consortium

**Affiliations:** 1 Center for Genomics and Personalized Medicine Research, Wake Forest School of Medicine, Winston-Salem, North Carolina, United States of America; 2 Center for Diabetes Research, Wake Forest School of Medicine, Winston-Salem, North Carolina, United States of America; 3 Center for Research on Genomics and Global Health, National Human Genome Research Institute, Bethesda, Maryland, United States of America; 4 Program on Genomics and Nutrition, School of Public Health, University of California Los Angeles, Los Angeles, California, United States of America; 5 Center for Metabolic Disease Prevention, School of Public Health, University of California Los Angeles, Los Angeles, California, United States of America; 6 Center for Public Health Genomics, University of Virginia, Charlottesville, Virginia, United States of America; 7 Department of Public Health Sciences, University of Virginia, Charlottesville, Virginia, United States of America; 8 Institute for Translational Genomics and Population Sciences, Los Angeles BioMedical Research Institute at Harbor-UCLA Medical Center, Torrance, California, United States of America; 9 Department of Medicine, University of Mississippi Medical Center, Jackson, Mississippi, United States of America; 10 Division of Epidemiology, Department of Health Sciences Research, Mayo Clinic, Rochester, Minnesota, United States of America; 11 The GeneSTAR Research Program, Division of General Internal Medicine, Department of Medicine, The Johns Hopkins University School of Medicine, Baltimore, Maryland, United States of America; 12 Laboratory of Neurogenetics, National Institute on Aging, National Institutes of Health, Bethesda, Maryland, United States of America; 13 Center for Public Health Genomics, Division of Public Health Sciences, Wake Forest School of Medicine, Winston-Salem, North Carolina, United States of America; 14 Department of Biostatistical Sciences, Division of Public Health Sciences, Wake Forest School of Medicine, Winston-Salem, North Carolina, United States of America; 15 Department of Preventive Medicine, Northwestern University Feinberg School of Medicine, Chicago, Illinois, United States of America; 16 Cardiovascular Health Research Unit, University of Washington, Seattle, Washington, United States of America; 17 Department of Medicine, University of Washington, Seattle, Washington, United States of America; 18 San Francisco Coordinating Center, California Pacific Medical Center Research Institute, San Francisco, California, United States of America; 19 Department of Epidemiology and Biomedical Informatics, Emory University, Atlanta, Georgia, United States of America; 20 Division of Statistical Genomics, Washington University School of Medicine, St. Louis, Missouri, United States of America; 21 Division of Sleep Medicine, Brigham and Women's Hospital, Boston, Massachusetts, United States of America; 22 The Charles Bronfman Institute for Personalized Medicine, Icahn School of Medicine at Mount Sinai, New York, New York, United States of America; 23 The Genetics of Obesity and Related Metabolic Traits Program, Icahn School of Medicine at Mount Sinai, New York, New York, United States of America; 24 Division of Epidemiology, Department of Medicine, Vanderbilt Epidemiology Center, Vanderbilt-Ingram Cancer Center, Vanderbilt University School of Medicine, Nashville, Tennessee, United States of America; 25 Division of Endocrinology, Metabolism and Molecular Medicine, Northwestern University Feinberg School of Medicine, Chicago, Illinois, United States of America; 26 Division of Public Health Sciences, Wake Forest School of Medicine, Winston-Salem, North Carolina, United States of America; 27 Department of Biochemistry, Wake Forest School of Medicine, Winston-Salem, North Carolina, United States of America; 28 Department of Biology, Center for Health Disparities, East Carolina University, Greenville, North Carolina, United States of America; 29 Department of Internal Medicine, Wake Forest School of Medicine, Winston-Salem, North Carolina, United States of America; 30 Medical Genetics Research Institute, Cedars-Sinai Medical Center, Los Angeles, California, United States of America; 31 Department of Medicine, University of Mississippi Medical Center, Jackson, Mississippi, United States of America; 32 Jackson State University, Tougaloo College, Jackson, Mississippi, United States of America; 33 Collaborative Studies Coordinating Center, Department of Biostatistics, University of North Carolina at Chapel Hill, Chapel Hill, North Carolina, United States of America; 34 Division of Epidemiology and Community Health, University of Minnesota, Minneapolis, Minnesota, United States of America; 35 Human Genetics Center, University of Texas Health Science Center at Houston, Houston, Texas, United States of America; 36 Division of Allergy and Clinical Immunology, Department of Medicine, The Johns Hopkins University School of Medicine, Baltimore, Maryland, United States of America; 37 Department of Epidemiology, Johns Hopkins Bloomberg School of Public Health, Baltimore, Maryland, United States of America; 38 Laboratory of Personality and Cognition, National Institute on Aging, National Institutes of Health, Baltimore, Maryland, United States of America; 39 Department of Epidemiology and Biostatistics, Case Western Reserve University, Cleveland, Ohio, United States of America; 40 Department of Medicine, Case Western Reserve University, MetroHealth System campus, Cleveland, Ohio, United States of America; 41 Department of Physiology and Biophysics, Case Western Reserve University, Cleveland, Ohio, United States of America; 42 Division of Epidemiology, Department of Medicine, Vanderbilt University Medical Center, Nashville, Tennessee, United States of America; 43 Department of Epidemiology, University of Washington, Seattle, Washington, United States of America; 44 Department of Biostatistics, University of Washington, Seattle, Washington, United States of America; 45 Department of Epidemiology and Prevention, Division of Public Health Sciences, Wake Forest School of Medicine, Winston-Salem, North Carolina, United States of America; 46 Department of Medicine, University of California, San Francisco, California, United States of America; 47 Department of Epidemiology, School of Public Health, University of Michigan, Ann Arbor, Michigan, United States of America; 48 Division of Epidemiology, Department of Medicine, Vanderbilt Epidemiology Center, Vanderbilt-Ingram Cancer Center, Vanderbilt University School of Medicine, Nashville, Tennessee; International Epidemiology Institute, Rockville, Maryland, United States of America; 49 Center for Genetic Medicine, Northwestern University Feinberg School of Medicine, Chicago, Illinois, United States of America; 50 Center for Human Genetics Research and Department of Molecular Physiology and Biophysics, Vanderbilt University, Nashville, Tennessee, United States of America; 51 Department of Twin Research and Genetic Epidemiology, King's College London, London, United Kingdom; 52 Child Health and Development Institute, Icahn School of Medicine at Mount Sinai, New York, New York, United States of America; 53 Department of Health Services, University of Washington, Seattle, Washington, United States of America; 54 Health Disparities Unit, National Institute on Aging, National Institutes of Health, Baltimore Maryland, United States of America; 55 Department of Health Policy and Management, Johns Hopkins Bloomberg School of Public Health, Baltimore, Maryland, United States of America; 56 Department of Physiology and Biophysics, University of Mississippi Medical Center, Jackson, Mississippi, United States of America; 57 Department of Medicine, University of Virginia, Charlottesville, Virginia, United States of America; 58 Department of Biochemistry and Molecular Genetics, University of Virginia, Charlottesville, Virginia, United States of America; 59 Department of Epidemiology, University of California Los Angeles, Los Angeles, California, United States of America; 60 Departments of Epidemiology and Medicine, Brown University, Providence, Rhode Island, United States of America; Wellcome Trust Sanger Institute, United Kingdom

## Abstract

Type 2 diabetes (T2D) is more prevalent in African Americans than in Europeans. However, little is known about the genetic risk in African Americans despite the recent identification of more than 70 T2D loci primarily by genome-wide association studies (GWAS) in individuals of European ancestry. In order to investigate the genetic architecture of T2D in African Americans, the MEta-analysis of type 2 DIabetes in African Americans (MEDIA) Consortium examined 17 GWAS on T2D comprising 8,284 cases and 15,543 controls in African Americans in stage 1 analysis. Single nucleotide polymorphisms (SNPs) association analysis was conducted in each study under the additive model after adjustment for age, sex, study site, and principal components. Meta-analysis of approximately 2.6 million genotyped and imputed SNPs in all studies was conducted using an inverse variance-weighted fixed effect model. Replications were performed to follow up 21 loci in up to 6,061 cases and 5,483 controls in African Americans, and 8,130 cases and 38,987 controls of European ancestry. We identified three known loci (*TCF7L2*, *HMGA2* and *KCNQ1*) and two novel loci (*HLA-B* and *INS-IGF2*) at genome-wide significance (4.15×10^−94^<*P*<5×10^−8^, odds ratio (OR) = 1.09 to 1.36). Fine-mapping revealed that 88 of 158 previously identified T2D or glucose homeostasis loci demonstrated nominal to highly significant association (2.2×10^−23^ < locus-wide *P*<0.05). These novel and previously identified loci yielded a sibling relative risk of 1.19, explaining 17.5% of the phenotypic variance of T2D on the liability scale in African Americans. Overall, this study identified two novel susceptibility loci for T2D in African Americans. A substantial number of previously reported loci are transferable to African Americans after accounting for linkage disequilibrium, enabling fine mapping of causal variants in trans-ethnic meta-analysis studies.

## Author Summary

Despite the higher prevalence of type 2 diabetes (T2D) in African Americans than in Europeans, recent genome-wide association studies (GWAS) were examined primarily in individuals of European ancestry. In this study, we performed meta-analysis of 17 GWAS in 8,284 cases and 15,543 controls to explore the genetic architecture of T2D in African Americans. Following replication in additional 6,061 cases and 5,483 controls in African Americans, and 8,130 cases and 38,987 controls of European ancestry, we identified two novel and three previous reported T2D loci reaching genome-wide significance. We also examined 158 loci previously reported to be associated with T2D or regulating glucose homeostasis. While 56% of these loci were shared between African Americans and the other populations, the strongest associations in African Americans are often found in nearby single nucleotide polymorphisms (SNPs) instead of the original SNPs reported in other populations due to differential genetic architecture across populations. Our results highlight the importance of performing genetic studies in non-European populations to fine map the causal genetic variants.

## Introduction

The prevalence of type 2 diabetes (T2D) among adults in the USA is currently 11.3%, with substantially higher prevalence in African Americans (18.7%) than in European Americans (10.2%) [1]. To date, genome-wide association studies (GWAS) have identified >70 susceptibility loci for T2D [2]–[8]. While it is known that T2D is heritable in African Americans [9], it is unclear how much heritability is explained by the known genetic associations discovered primarily from European ancestry populations and whether there are risk loci specific to African Americans. Given that individuals of African ancestry tend to harbor more genetic diversity than individuals of other ancestries [10], we hypothesized that large-scale association analyses in African Americans could shed light on the genetic architecture of T2D and the risk attributable to cosmopolitan *vs*. population-specific variants.

## Results

### Study overview

We conducted a meta-analysis of 17 African American GWAS on T2D comprising 8,284 cases and 15,543 controls (Tables S1 and S2). Missing genotypes in individual studies were imputed to one of the HapMap reference panels (Phase II release 21–24 CEU+YRI, Phase II release 22 all populations, Phase II+III release 27 CEU+YRI, Phase II+III release 27 CEU+YRI+ASW or Phase II+III release 27 all populations) using MACH, IMPUTE2 or BEAGLE (Table S3). Genomic control corrections [11] were applied to each study (λ = 1.01–1.08) and after meta-analysis (λ = 1.06) due to modest inflated association results (Table S3) [12]. Association results for ∼2.6M SNPs were subsequently examined.

From stage 1 meta-analysis, 49 SNPs moderately associated with T2D (*P*<1×10^−5^) and two candidate SNPs near the p value threshold (rs231356 at *KCNQ1*, *P* = 2.84×10^−5^ and rs2244020 at *HLA-B*, *P* = 1.02×10^−5^) totaling 51 SNPs in 21 loci were followed up for replication. rs231356 is 14 kb downstream of the reported T2D index SNP, rs231362, in Europeans [3]. Moderate associations have also been observed across the *HLA* region in Europeans [3]. The stage 2 replication included *in silico* and *de novo* replication in up to 11,544 African American T2D cases and controls, as well as *in silico* replication in 47,117 individuals of European ancestry from DIAGRAMv2 [3] (Table S4). Meta-analyses were performed to combine results from African Americans (stage 1+2a, *n*≤35,371, Table S4) and both African Americans and Europeans (stage 1+2a+2b, *n*≤82,488, Table S4).

### T2D loci reaching genome-wide significance

**Figure 1:**
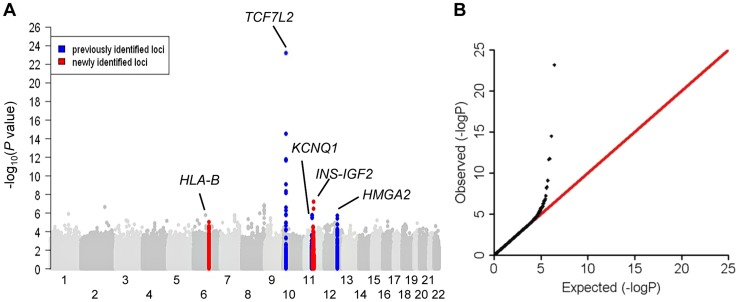
Association results of stage 1 meta-analysis in African Americans in a model adjusted for age, sex, study sites and study-specific principle components. (**A**) Manhattan plot. Previously identified loci are denoted in red. Novel loci identified in this study are denoted in blue. (**B**) Quantile-quantile plot. The observed *P* values (*y* axis) were compared with the expected *P* values under the null distribution (*x* axis).

Five independent loci reached genome-wide significance (*P*<5×10^−8^). Stage 1 meta-analysis identified the established *TCF7L2* locus. Stage 1+2a meta-analysis identified the established *KCNQ1* and *HMGA2* loci. Stage 1+2a+2b meta-analysis identified a second signal at *KCNQ1* and a novel *HLA-B* locus. Secondary analysis including body mass index (BMI) adjustment in stage 1+2a meta-analysis identified the second novel locus at *INS-IGF2* (Table 1 and Figure 1). None of the most strongly associated SNPs at these loci demonstrated significant heterogeneity of effect sizes among studies within each stage, between African Americans in stages 1 and 2a, or between African Americans in stage 1+2a and Europeans in stage 2b after Bonferroni correction of multiple comparisons (*P*_het_>0.001) (Figure S1).

**Table 1:** Novel and previously identified loci associated with T2D at *P*<5×10^−8^.

						Stage 1 GWAS meta-analysis in in African Americans: up to 8,284 cases and 15,543 controls			Stage 2a replication in African Americans: up to 6,061 cases and 5,483 controls			Stage 1+2a meta-analysis in African Americans: up to 14,345 cases and 21,026 controls			Stage 2b replication in Europeans (DIAGRAMv2): up to 8,130 cases and 38,987 controls		Stage 1+2a+2b meta-analysis of all African Americans and Europeans: up to 22,475 cases and 60,013 controls		
Loci	Chr	Position (Build 36)	SNP	Alleles^a^	RAF^b^	OR (95% CI)	*P*	*P*_het_	OR (95% CI)	*P*	*P*_het_	OR (95% CI)	*P*	*P*_het_	OR (95% CI)	*P*	OR (95% CI)	*P*	*P*_het_
**Previously identified T2D loci**																			
***TCF7L2*** c	10	114748339	rs7903146	T/C	0.30	1.32 (1.25–1.4)	**6.62E-24**	1.81E-01	1.34 (1.26–1.43)	**8.38E-20**	6.01E-03	1.33 (1.28–1.39)	**4.78E-44**	7.34E-01	1.4 (1.34–1.46)	**2.21E-51**	1.36 (1.32–1.4)	**4.15E-94**	1.16E-01
***KCNQ1*** c	11	2661919	rs231356	T/A	0.27	1.14 (1.07–1.21)	2.84E-05	9.11E-01	1.05 (0.98–1.14)	1.68E-01	3.26E-01	1.11 (1.06–1.16)	1.94E-05	1.08E-01	1.08 (1.04–1.13)	4.37E-04	1.09 (1.06–1.13)	**3.93E-08**	5.27E-01
***KCNQ1*** c	11	2806106	rs2283228	A/C	0.89	1.22 (1.14–1.31)	6.10E-08	9.48E-02	1.17 (1.06–1.28)	1.04E-03	7.10E-01	1.2 (1.14–1.27)	**9.90E-11**	4.34E-01	1.16 (1.06–1.26)	9.73E-04	1.19 (1.13–1.24)	**4.87E-13**	4.90E-01
***HMGA2*** c	12	64537207	rs343092	T/G	0.81	1.16 (1.09–1.24)	1.91E-06	9.48E-01	1.15 (1.04–1.26)	3.99E-03	3.37E-01	1.16 (1.1–1.22)	**8.79E-09**	7.93E-01	1.12 (1.06–1.19)	5.43E-05	1.14 (1.1–1.19)	**2.75E-12**	4.41E-01
**Newly identified T2D loci**																			
***HLA-B*** c	6	31455430	rs2244020	G/A	0.69	1.12 (1.06–1.17)	1.02E-05	2.11E-02	1.1 (0.98–1.22)	1.01E-01	1	1.11 (1.07–1.16)	1.14E-06	7.57E-01	1.07 (1.03–1.12)	7.67E-04	1.09 (1.06–1.13)	**6.57E-09**	2.55E-01
***INS-IGF2*** d	11	2135246	rs3842770	A/G	0.23	1.18 (1.11–1.25)	8.18E-08	7.16E-01	1.07 (0.99–1.16)	8.09E-02	7.16E-01	1.14 (1.09–1.19)	**2.78E-08**	7.37E-02	-	-	-	-	-

Abbreviations: Chr, chromosome; SNP, single nucleotide polymorphism; RAF, risk allele frequency; OR, odds ratio for risk allele; CI, confidence interval; *P*_het_, heterogeneity *P* value.

a Alleles are ordered as risk allele/other allele aligned to the forward strand of NCBI Build 36.

b Risk allele frequency in Stage 1 samples.

c Associations were performed with adjustment for age, sex, study sites, and study-specific principal components.

d Associations were performed with adjustment for age, sex, study sites, study-specific principal components and body mass index.

*P*<5×10^−8^ are in bold.

**Figure 2:**
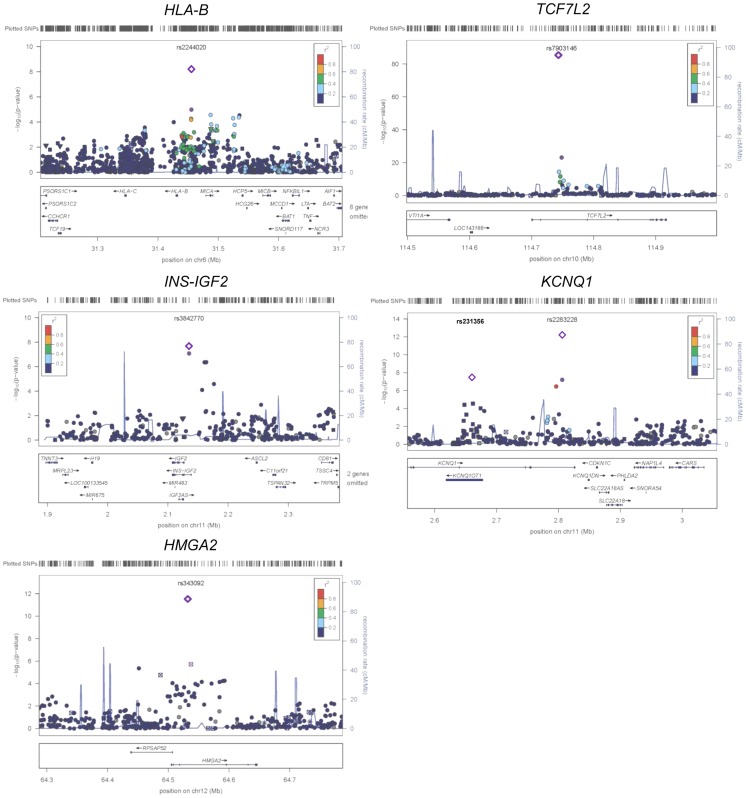
Regional plots of five previously and newly identified T2D loci in African Americans. Association *P* values (on a −log_10_ scale) of genotyped and imputed SNPs from stage 1 meta-analysis are plotted as a function of genomic position (NCBI Build 36). Plots for *HLA-B*, *TCF7L2*, *KCNQ1*, and *HMGA2* used the model without BMI adjustment whereas plots for *INS-IGF2* used the model with BMI adjustment. In each panel, the most strongly associated SNP from stage 1 and stage 1+2a+2b meta-analysis is denoted by a purple circle and a purple diamond, respectively. The color of all other SNPs indicates LD with the most strongly associated SNP based on the HapMap 2 YRI data. At *KCNQ1*, two independent signals are shown.

At the *TCF7L2* locus, the most strongly associated SNP in stage 1+2a African Americans samples was rs7903146 (OR = 1.33, *P* = 4.78×10^−44^, Table 1 and Figure 2). rs7903146 is also the index SNP (most significantly associated with T2D in prior studies) in Europeans (OR = 1.40, *P* = 2.21×10^−51^) [3], South Asians (OR = 1.25, *P* = 3.4×10^−19^) [4] and East Asians (OR = 1.48, *P* = 2.44×10^−15^) [13].

Two association signals were observed at *KCNQ1* (Table 1 and Figure 2). The first association signal was represented by rs2283228 located at the 3′ end of *KCNQ1* (stage 1+2a OR = 1.20, *P* = 9.90×10^−11^; stage 1+2a+2b OR = 1.19, *P* = 4.87×10^−13^). Using data from individuals of African ancestry in Southwest USA (ASW) from the 1000 Genomes Project (1KGP) [14], rs2283228 mapped to the same linkage disequilibrium (LD)-based interval as index SNPs from other populations (rs2283228 [15] and rs2237892 [16]–[17] in Japanese, rs2237892 in Hispanics [18], rs163182 [19] and rs2237895 [20] in Han Chinese). The second association signal was represented by rs231356 (r^2^ = 0 with rs2283228 in both ASW and CEU) (stage 1+2a OR = 1.11, *P* = 1.94×10^−5^; stage 1+2a+2b OR = 1.09, *P* = 3.93×10^−8^), located 144 kb upstream of the first signal. rs231356 is located at the same LD interval as the index SNPs rs231362 in Europeans [3] and rs231359 in Chinese [20].

At the *HMGA2* locus, the most strongly associated SNP was rs343092 (stage 1+2a OR = 1.16, *P* = 8.79×10^−9^; stage 1+2a+2b OR = 1.14, *P* = 2.75×10^−12^; Table 1 and Figure 2). rs343092 is located 76 kb downstream and at the same LD interval as of the index SNP rs1531343 reported in Europeans [3].

Two novel T2D loci were identified. The effect sizes of rs2244020 located near *HLA-B* were similar in African Americans and Europeans (OR = 1.11 *vs*. 1.07, *P*_het_ = 0.26; stage 1+2a+2b *P* = 6.57×10^−9^) (Table 1 and Figure 2). *HLA-B* encodes the class I major histocompatibility complex involved in antigen presentation in immune responses.

The most strongly associated SNP near *INS-IGF2* was rs3842770 in African Americans (OR = 1.14, *P* = 2.78×10^−8^, stage 1+2a BMI adjusted, Table 1 and Figure 2) but the risk A allele was absent in the CEU population. Insulin plays a key role in glucose homeostasis. Mutations at *INS* lead to neonatal diabetes, type 1 diabetes, and hyperinsulinemia [21]. Insulin-like growth factor 2 (IGF2) is involved in growth and development. IGF2 overexpression in transgenic mice leads to islet hyperplasia [22] and IGF2 deficiency in the Goto–Kakizaki rat leads to beta cell mass anomaly [23].

### Associations at previously reported T2D and glucose homeostasis loci

We investigated index SNPs from 158 independent loci associated with T2D and/or glucose homeostasis from prior genome-wide and candidate gene studies in individuals of European, East Asian, South Asian, or African American ancestry (Table S5). Among the 104 T2D-associated index SNPs, 19 were associated with T2D in stage 1 African American samples (*P*<0.05). Most of the 17 T2D-associated SNPs that showed consistent direction of effects had similar effect sizes between this study and prior reports, despite that rs10440833 at *CDKAL1* had substantially stronger effect size in Europeans (OR = 1.25) than in African Americans (OR = 1.06, *P*_het_ = 5.86×10^−6^). Additionally, 3 out of 54 trait-increasing alleles from glucose homeostasis-associated index SNPs were associated with increased T2D risk in African Americans (*P*<0.05).

We also performed a locus-wide analysis to test for associations of all SNPs within the LD region at r^2^≥0.3 with the previously reported index SNPs and results were corrected for the effective number of SNPs [24]. Since the causal variant(s) at each locus may be different or reside on different haplotypes across populations with different LD structures, this approach allows the identification of the most strongly associated SNPs in African Americans that may or may not be in LD with the index SNPs reported in other populations. A total of 55 T2D- and 29 glucose-associated loci were associated with T2D in African Americans (*P_locus_*<0.05, corrected for LD in ASW for SNPs within a locus; Table S6). We compared the genetic architecture between the previously reported index SNPs and our fine-mapped SNPs for these 84 loci. The respective average risk allele frequencies were 0.51 and 0.46, and the distributions or pairwise differences of risk allele frequencies were not significantly different (*P* = 0.255, Wilcoxon rank sum test; and *P* = 0.295, Wilcoxon signed-rank test, respectively, Figure S2). In contrast, the average odds ratios for the risk alleles were higher for the fine-mapped SNPs as compared to the index SNPs (1.14 *vs*. 1.05). The distributions and pairwise differences of risk allele odds ratios were significantly different (*P* = 1.18×10^−19^ and 5.55×10^−14^, respectively, Figure S2). Thus, the locus-wide analysis identified variants with larger effect sizes and similar allele frequencies.

We leveraged differences in LD between African Americans and Europeans to fine-map and re-annotate several established loci. The association signal spanning ∼100 kb at *INTS8* in African Americans overlapped the ∼200 kb *TP53INP1* T2D locus in Europeans [3]. The most strongly associated SNP in MEDIA tended to have larger effect size in African Americans than in Europeans (rs17359493, OR = 1.13 vs. 1.06, *P* = 1.39×10^−7^ vs. 3.20×10^−2^, respectively, *P*_het_ = 0.06) (Table S4). However, rs17359493 at intron 10 of *INTS8* was only in weak LD with the reported index SNP rs896854 in Europeans (r^2^ = 0.21 in CEU, 0.10 in ASW). Neither the reported index SNP rs896854 nor its proxies from the CEU data demonstrated significant association to T2D in African Americans (Table S6 and Figure S3a,b), suggesting that rs17359493 may be an independent novel signal. *INTS8* encodes a subunit of the integrator complex which is involved in the cleavage of small nuclear RNAs. At *KCNQ1*, the most strongly associated SNP rs231356 was in weak LD with the index SNP rs231362 reported in Europeans [3] (r^2^ = 0.24 in CEU and 0.17 in ASW). Given rs231362 was modestly associated with T2D in African American (*P* = 0.04) and was in weak LD (r^2^ = 0.21 to 0.46 in CEU) with other associated SNPs in this region (Table S6 and Figure S3c,d), the results suggest a refinement of the localization of causal variant(s) to variants in strong LD with rs231356. At *HMGA2*, the most strongly associated SNP rs343092 was in moderate LD with the index SNP rs1531343 (r^2^ = 0.60 in CEU and 0.32 in ASW). Despite rs1531343 and its proxies in high LD were not associated with T2D in African Americans (*P*>0.05), several SNPs in moderate LD, including rs343092, showed nominal to strong associations (Table S6 and Figure S3e,f). Trans-ethic fine mapping will be particularly useful to dissect the causal variant(s) at this locus.

### Effect of obesity on T2D susceptibility loci

We investigated the influence of obesity by comparing the stage 1 meta-analysis results with or without adjustment for BMI at the 51 most significantly associated SNPs from the GWAS for follow up (Tables S4 and S7) and 158 established T2D or glucose homeostasis index SNPs (Table S5). Association results were highly similar with and without BMI adjustment (correlation coefficients were 0.99 for both effect sizes and −log*P* values). Of particular note, *FTO* is suggested to influence T2D primarily through modulation of adiposity in Europeans [3], [25], but evidence is contradictory across multiple ethnic groups [26]–[28]. The index SNP rs11642841 was not significantly associated with T2D in African Americans without and with BMI adjustment (*P* = 0.06 and 0.23, respectively) (Table S5). The frequency of the risk A allele was 0.13 in this study. It had 100% power to detect association at the reported OR of 1.13 at type 1 error rate of 0.05, suggesting that *FTO* is unlikely a key T2D susceptibility gene in African Americans.

### Gene expression and bioinformatics analyses

Among the six genome-wide significant loci (Table 1), we found no coding variants in the most significantly associated SNPs or their proxies. These SNPs demonstrated only weak associations with expression quantitative trait loci (eQTLs) (*P*>0.001, Table S8). Examination of the ENCODE data [29] revealed that several SNPs at *TCF7L2*, *KCNQ1*, and *HMGA2* were located at protein binding sites or were predicted to alter motif affinity for transcription factors implicated in energy homeostasis (Table S9). The most strongly associated SNP rs7903146 in *TCF7L2* is predicted to alter the binding affinity for a POU3F2 regulatory motif [30]. POU3F2 is a neural transcription factor that enhances the activation of genes regulated by corticotropin-releasing hormone which stimulates adrenocorticotropic hormone (ACTH). ACTH is synthesized from pre-pro-opiomelanocortin (pre-POMC) which regulates energy homeostasis. For the 3′ signal at *KCNQ1*, several tag SNPs are predicted to alter the binding affinity for regulatory motifs, including SREBP, CTCF and HNF4A. SREBP is a transcription factor involved in sterol biosynthesis. CTCF regulates the expression of IGF2 [31]. HNF4A is a master regulator of hepatocyte and islet transcription. The tag SNP rs2257883 at *HMGA2* is predicted to alter the binding affinity of MEF2, which regulates GLUT4 transcription in insulin responsive tissues [32].

## Discussion

We have performed the largest genetic association analysis to date for T2D in African Americans. Our data support the hypothesis that risk for T2D is partly attributable to a large number of common variants with small effects [7]. We identified *HLA-B* and *INS-IGF2* as novel T2D loci, the latter specific to African Americans. We found evidence supporting association for 88 previously identified T2D and glucose homeostasis loci. Taken together, these 90 loci yielded a sibling relative risk of 1.19. The phenotypic variance measured on the liability scale is substantially larger in African Americans than in European Americans (17.5% *vs*. 5.7%) [7] due to larger effect sizes upon fine-mapping as well as higher disease prevalence in African Americans.

The two novel T2D loci, *HLA-B* and *INS-IGF2*, have been implicated in type 1 diabetes (T1D) risk in Europeans [33]–[35]. One limitation of our study is the lack of autoantibody measurement. However, our results are unlikely to be confounded by the presence of misclassified patients. Among diabetic youth aged <20 years, T2D characterized by insulin resistance without autoimmunity is more prevalent in African Americans (40.1%) than in European Americans (6.2%), while African Americans less often present with autoimmunity and insulin deficiency resembling T1D compared to European Americans (32.5% vs. 62.9%, respectively) [36]. Autoimmunity is also uncommon in African American diabetic adults [37]. Furthermore, associations for T1D are stronger at *HLA* class II (*HLA-DRB1*, *-DQA1*, and -*DQB1*) than *HLA* class I regions in Europeans [33]–[34], [38]–[41] (http://www.t1dbase.org). In African Americans, T1D individuals showed both shared and unique risk and protective *HLA* class II haplotypes as compared to European T1D individuals [42]–[43]. More importantly, these individuals also showed substantially stronger associations at *HLA* class II (*P*<1×10^−25^) than class I regions (*P*<1×10^−5^) [42], which is in contradiction with our finding of stronger associations at *HLA* class I than class II regions in T2D individuals (*HLA*-*B*, Figure S4). The observed *HLA-B* association may be due to LD with nearby causal gene(s) since there is long range LD in this region. Recently, rs3130501 near *POU5F1* and *TCF19* was reported for association with T2D in a trans-ancestry meta-analysis [8]. rs3130501 was located 211 kb upstream of rs2244020 and mapped to the same LD interval. However, the two SNPs were not correlated in both CEU (D′ = 0.57, r^2^ = 0.05) and ASW (D′ = 0.68, r^2^ = 0.16) from 1KGP nor strongly associated with T2D in the stage 1 meta-analysis (*P* = 0.04). Other potential non-*HLA* candidate genes may include *TNFA* which regulates immune and inflammatory response. It has been hypothesized that activated innate and adaptive immune cells stimulate release of cytokines such as TNFα and IL-1β, which promote both systemic insulin resistance and β-cell damage [44]. On the other hand, evidence has implicated T1D loci *HLA-DQ/DR*, *GLIS3* and *INS* in the susceptibility of latent autoimmune diabetes in adults (LADA) and/or T2D [7], [34], [45]–[46], while T2D loci such as *PPARG* and *TCF7L2* was associated with T1D [47] and LADA [46], [48], respectively. More comprehensive studies are needed to understand the shared and distinct genetic risks in different forms of diabetes which will facilitate diagnosis and personalized treatment.

Our results have several implications regarding the genetic architecture of T2D. First, fine-mapping suggests that currently known loci explain more of the risk than previously estimated. Second, the loci conferring the largest risk for T2D appear to act through regulatory rather than protein-coding changes. Third, many, but not all, of the previously identified T2D loci are shared across ancestries. The differential LD structure of African-ancestry populations at shared loci provides an opportunity for fine mapping in trans-ethnic meta-analysis. Fourth, the ∼2.6M MEDIA SNPs achieved only 43.3% coverage of the 1KGP ASW common SNPs, suggesting that risk loci that are specific to African-ancestry individuals are difficult to discover with the genotyping arrays being used. Large-scale sequencing studies, such as those focusing on whole genomes, exomes, and targeted resequencing for associated non-coding regions, will be necessary to further delineate the causal variants for T2D risk in African Americans.

## Materials and Methods

### Samples and clinical characterization

Stage 1 discovery samples included 17 T2D GWAS studies (ARIC, CARDIA, CFS, CHS, FamHS, GeneSTAR, GENOA, HANDLS, Health ABC, HUFS, JHS, MESA, MESA Family, SIGNET-REGARDS, WFSM, FIND, and WHI) with up to 23,827 African American subjects (8,284 cases and 15,543 controls). Stage 2 replication samples included up to 11,544 African American subjects (6,061 cases and 5,483 controls), using *in silico* replication of GWAS data from eMERGE and IPM Biobank and *de novo* genotyping in IRAS, IRASFS, SCCS, and WFSM. In general, T2D cases were defined as having at least one of the following: fasting plasma glucose ≥126 mg/dl, 2 hour glucose during oral glucose tolerance test (OGTT) ≥200 mg/dl, random glucose ≥200 mg/dl, oral hypoglycemic agent or insulin treatment, or physician-diagnosed diabetes. All cases were diagnosed at ≥25 years (or age at study ≥25 years if age at diagnosis was not available). For cohort studies, individuals who met the criteria at any of the visits were defined as cases. Controls with normal glucose tolerance (NGT) were defined by satisfying all the following criteria: fasting plasma glucose <100 mg/dl, 2 hour OGTT<140 mg/dl (if available), no treatment of diabetes, and age ≥25 years. For cohort studies, individuals who met the criteria at all visits were defined as controls. All study participants provided written informed consent, except for eMERGE that use an opt out program, and approval was obtained from the institutional review board (IRB) from the respective local institutions. Detailed descriptions of the participating studies are provided in Text S1.

### Genotyping, imputation and quality control

For stage 1 and 2 GWAS studies, genotyping was performed with Affymetrix or Illumina genome-wide SNP arrays. Imputation of missing genotypes was performed using MACH [49], IMPUTE2 [50] or BEAGLE [51] using HapMap reference haplotypes. For each study, samples reflecting duplicates, low call rate, gender mismatch, or population outliers were excluded. In general, SNPs were excluded by the following criteria: call rate <0.95, minor allele frequency (MAF)<0.01, minor allele count <10, Hardy-Weinberg P-value <1×10^−4^, or imputation quality score <0.5 (Table S3). For *de novo* replication studies, genotyping was performed using the Sequenom MassArray platform (Sequenom; San Diego, CA). Sample and SNP quality controls were performed as with GWAS data.

### Statistical analysis

Single SNP association was performed for each study by regressing T2D case/control status on genotypes. To account for uncertainty of genotype calls during imputation, genotype probabilities or dosage were used for association tests in imputed SNPs. The association tests assumed an additive genetic model and adjusted for age, sex, study centers, and principal components. Principal components were included to control for confounding effects of admixture proportion and population structure. Secondary analysis with additional adjustment for BMI was performed for SNPs with *P*<1×10^−5^ in stage 1 meta-analysis and index SNPs previously reported to be associated with T2D or glucose homeostasis traits. BMI adjustment allows increasing power to detect T2D loci independent of BMI effect and diminish associations at T2D loci with effects modulated through BMI. Logistic regression was used for samples of unrelated individuals. Generalized estimating equations [52] or SOLAR [53] were used for samples of related individuals. Association results with extreme values (absolute beta coefficient or standard error >10), primarily due to low cell counts resulting from small sample sizes and/or low minor allele frequencies, were excluded (Table S3).

### Meta-analysis

In stage 1, association results were combined by a fixed effect model with inverse variance weighted method using the METAL software [12]. Genomic control correction [11] was applied to each study before meta-analysis, and to the overall results after meta-analysis. Results from SNPs genotyped in <10,000 samples and those with allele frequency difference >0.3 among studies were excluded. A total of 2,579,389 SNPs were analyzed in the meta-analysis (Table S3). In stage 2a, association results from African American replication studies were also combined using a fixed effect inverse variance weighted method. To assess the overall effects in African Americans (stage 1+2a) and both African Americans and Europeans (stage 1+2a+2b), association results from studies in the respective stages were combined using a fixed effect inverse variance weighted method. Genome-wide significance is declared at *P*<5×10^−8^ from the meta-analysis result of all stages, which has better power than the replication-based strategy [54].

Among the 51 SNPs carried forward for replication, heterogeneity of effect sizes across studies within each stage was assessed using Cochran's Q statistic implemented in METAL. Meta-analysis results from stages 1 and 2a, stage 1+2a and 2b were used to assess heterogeneity of effect sizes between discovery and replication stages in African Americans, and between African Americans and Europeans, respectively. For SNPs with significant heterogeneous effect size after multiple comparison corrections (*P*_het_<0.001), meta-analysis results including studies of all stages assessed by the random effect model implemented in GWAMA [55] were reported. Heterogeneous associations may partly due to differences in ascertainment scheme across studies. For index SNPs reported in prior studies, assessment of heterogeneity using Cochran's Q statistic between prior studies and this study were also reported.

### Transferability analysis

Index SNPs associated with T2D or glucose homeostasis traits from prior GWAS and candidate gene studies were examined for association with T2D in African Americans (Table S5). For the index SNP association tests, a per-SNP *P* value <0.05 was defined as significant. In the locus-wide analysis, the boundaries of a locus were defined by the most distant markers (within ±500 kb) using the 1KGP CEU data with r^2^≥0.3 with the index SNP. All MEDIA SNPs within these bounds were examined for association analysis. All pairwise LD values within each locus were estimated using the 1KGP CEU and ASW data. To estimate the effective number of SNPs at a locus, we retrieved genotypes from the 1KGP ASW data for markers present in MEDIA, estimated the sample covariance matrix from those genotypes, and spectrally decomposed the covariance matrix [24]. The effective number of SNPs was estimated using the relationship 
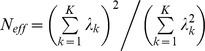
, in which *λ_k_* is the *k* ^th^ eigenvalue of the *K*×*K* covariance matrix for the *K* SNPs in the locus [24]. The per-locus significance level was defined as 0.05/effective number of SNPs (Table S6). By accounting for all SNPs within the bounds of LD, the per-locus significance level is corrected to account for markers in LD with the index SNP as well as markers not in LD with the index SNP, thereby potentially allowing for discovery of new associations at markers not tagged by the index SNP.

### Liability-scale variance explained

For each independent locus, we estimated the sibling relative risk using the most strongly associated SNP within that locus. Let *p_i_* and *ψ_i_* be the risk allele frequency and the corresponding odds ratio at the *i* ^th^ SNP, respectively. Assuming the additive genetic model and independence between SNPs, the contribution to the sibling relative risk *λ_s_* for a set of *N* SNPs is given by 
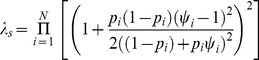
 [56]. Let *K* be the disease prevalence. The liability-scale variance 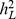 explained by the set of *N* SNPs is given by 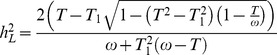, in which 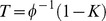, 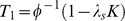, and 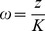, with 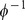 representing the standard normal quantile function and *z* representing the standard normal density at *T* [57].

### Coverage

The coverage of MEDIA SNPs to the human genome was estimated using HaploView [58] via pairwise tagging at the r^2^ = 0.8 threshold. We used all SNPs with minor allele frequencies ≥1% in both MEDIA and the 1KGP ASW sequence data. Coverage was estimated using non-overlapping bins of 1,000 SNPs.

### Power analysis

Study power was calculated using the genetic power calculator [59]. For SNPs with MAF≥0.3, our study had >80% power to detect odds ratios for T2D at OR≥1.06 and ≥1.13 at *P*<0.05 and *P*<5×10^−8^, respectively, in stage 1 samples under an additive model. The observed odds ratios among our stage 1 most significantly associated SNPs with *P*<1×10^−5^ ranged from 1.11 to 1.56 (Table S4). Given our African American sample size in stage 1+2a, our study had >80% power to detect OR≥1.1 at *P*<5×10^−8^ at MAF≥0.3, thus provided good power to detect genome-wide significance among the most significantly associated SNPs using all African American samples. For T2D SNPs reported from the literature, power was also calculated from the reported effect size using the risk allele frequency from this study for stage 1 samples at *P*<0.05 and *P*<5×10^−8^, respectively (Table S5).

### Gene expression analysis

The MuTHER resource (www.muther.ac.uk) includes lymphoblastoid cell lines (LCLs), skin, and adipose tissue derived simultaneously from a subset of well-phenotyped healthy female twins from the TwinsUK adult registry [60]. Whole-genome expression profiling of the samples, each with either two or three technical replicates, was performed using the Illumina Human HT-12 V3 BeadChips (Illumina Inc.) according to the protocol supplied by the manufacturer. Log_2_-transformed expression signals were normalized separately per tissue as follows: quantile normalization was performed across technical replicates of each individual followed by quantile normalization across all individuals. Genotyping was performed with a combination of Illumina arrays (HumanHap300, HumanHap610Q, 1M-Duo, and 1.2MDuo 1M). Untyped HapMap2 SNPs were imputed using the IMPUTE2 software package. In total, 776 adipose and 777 LCL samples had both expression profiles and imputed genotypes. Association between all SNPs (MAF>5%, IMPUTE info >0.8) within a gene or within 1 Mb of the gene transcription start or end site and normalized expression values were performed with the GenABEL/ProbABEL packages [61]–[62] using the polygenic linear model incorporating a kinship matrix in GenABEL followed by the ProbABEL mmscore score test with imputed genotypes. Age and experimental batch were included as cofactors.

Genotype and gene expression in LCL in HapMap samples were also available [63]. Association of genotypes and gene expression of transcripts within 1 MB of tested SNPs were analyzed separately for CEU and YRI populations. The variance components model implemented in SOLAR was used for association analysis which accounts for correlation among related individuals [53].

In this study, we examined the association of the most significantly associated SNPs from the six genome-wide significant loci and their proxies (r^2^≥0.8 in ASW) within 1 Mb of the associated SNPs with *cis*-expression quantitative trait loci (eQTLs) in peripheral blood leukocytes (LCL) and adipose tissue (Table S8).

### ENCODE data analysis

We examined putative function of non-coding genome-wide significant SNPs and their proxies within 1 Mb (r^2^≥0.8 in 1KGP ASW) using HaploReg [30] and RegulomeDB [64]. These databases interrogated multiple chromatin features from the Encyclopedia of DNA Elements (ENCODE) project [29]. High priority was given to variants annotated as protein-binding via ChIP-seq, and motif-changing via position weight matrices, with the respective transcription factors implicated in diabetes pathogenesis and related biological processes.

## Supporting Information

Figure S1 Forest plots of the most strongly associated SNPs at five previously and newly identified T2D loci in African Americans. Odds ratio and 95% CIs are presented for individual studies (black circle and line) and meta-analysis results (red diamond and line). At *KCNQ1*, two independent associated SNPs are shown.
(PDF)

Figure S2 (A) Distributions of risk allele frequencies for the previously reported index SNPs (in black) vs. the MEDIA most strongly associated SNPs (in red) in African Americans from stage 1 meta-analysis. (B) Distributions of odds ratios for risk alleles of the index SNPs (in black) vs. the most strongly associated MEDIA SNPs (in red) in African Americans from stage 1 meta-analysis.
(PDF)

Figure S3 Regional plots of stage 1 meta-analysis association results in African Americans for the most strongly associated SNPs from this study and the index SNPs from previous studies. (A–B) *INTS8-TP53INP1* region; (C–D) *KCNQ1* region; (E–F) *HMGA2* region. (A, C, E) The most strongly associated SNP in MEDIA is denoted by a purple circle and a red arrow with LD colored based on the HapMap 2 YRI data. (B, D, F) The index SNP is denoted by a purple circle and a blue arrow with LD colored based on the HapMap 2 CEU data.
(PDF)

Figure S4 Regional plots of *HLA-B* and *HLA-DQ/DR* regions for (A, C) stage 1 meta-analysis association results in African Americans and HapMap 2 YRI LD data, and (B, D) stage 3 DIAGRAMv2 results in Europeans using HapMap 2 CEU LD data. (A, B) The most strongly associated SNP rs2244020 at *HLA-B* region from this study is denoted by a purple circle and a red arrow. (C, D) The index SNP rs9272346 from Burton PR *et al* (2007) [65] is denoted by a purple circle and a blue arrow.
(PDF)

Table S1 Design of studies in stage 1 GWAS and stage 2a replication in African Americans.
(PDF)

Table S2 Clinical characteristics of study samples in stage 1 GWAS and stage 2a replication studies in African Americans.
(PDF)

Table S3 Genotyping methods, quality controls, imputation and statistical analysis in stage 1 GWAS and stage 2a replication studies in African Americans.
(PDF)

Table S4 SNPs with *P* value≤1×10^−5^ from stage 1 GWAS meta-analysis (BMI unadjusted) selected for stage 2 *in silico* and *de novo* replication in African Americans and *in silico* replication in individuals of European ancestry from DIAGRAMv2.
(PDF)

Table S5 Stage 1 GWAS meta-analysis results for index SNPs at established T2D or glucose homeostasis loci in African Americans.
(PDF)

Table S6 Locus-wide association at established T2D or glucose homeostasis loci in stage 1 GWAS meta-analysis in African Americans.
(PDF)

Table S7 BMI-adjusted association for SNPs from stage 1 GWAS meta-analysis selected for replication.
(PDF)

Table S8 Expression Quantitative Trait Loci (eQTL) analysis for the genome-wide significant SNPs for T2D. Results are shown for suggestive evidence of *cis*-association (*P*<0.05) between the genome-wide significant SNPs and their proxies with the genes within 1 Mb of the associated SNPs.
(PDF)

Table S9 Putative regulatory SNPs predicted from the ENCODE project for the genome-wide significant SNPs and their proxies at *TCF7L2*, *INS-IGF2*, *KCNQ1* and *HMGA2*.
(PDF)

Text S1 Description of GWAS and replication studies.
(PDF)
