# Supplementary figures and images for: Meta-Analysis of Genome-Wide Association Studies in African Americans Provides Insights into the Genetic Architecture of Type 2 Diabetes
Source: PLoS Genet. 2014 Aug 7;10(8):e1004517. doi: 10.1371/journal.pgen.1004517 (PMC4125087; doi:10.1371/journal.pgen.1004517)

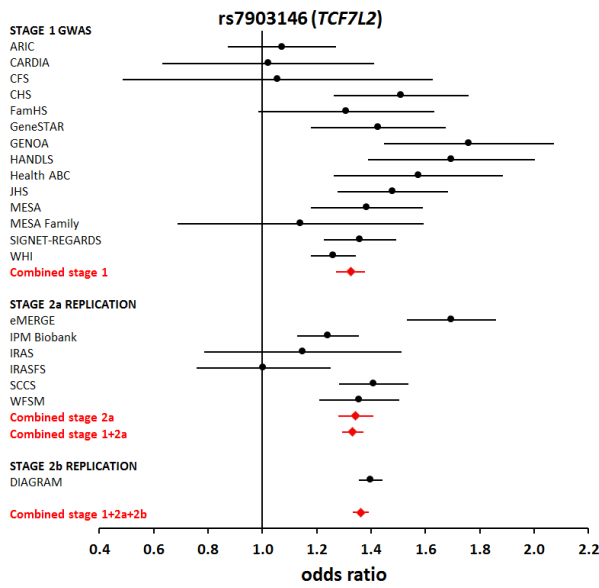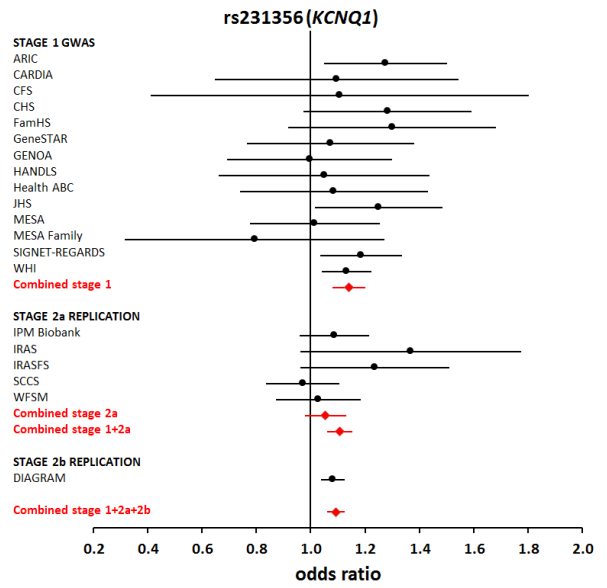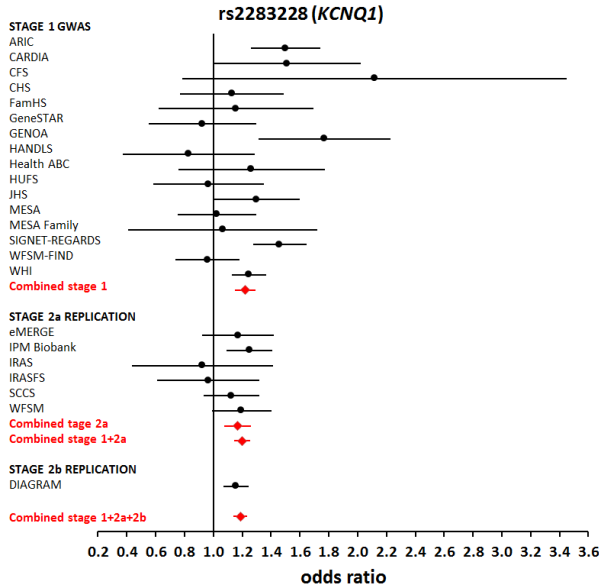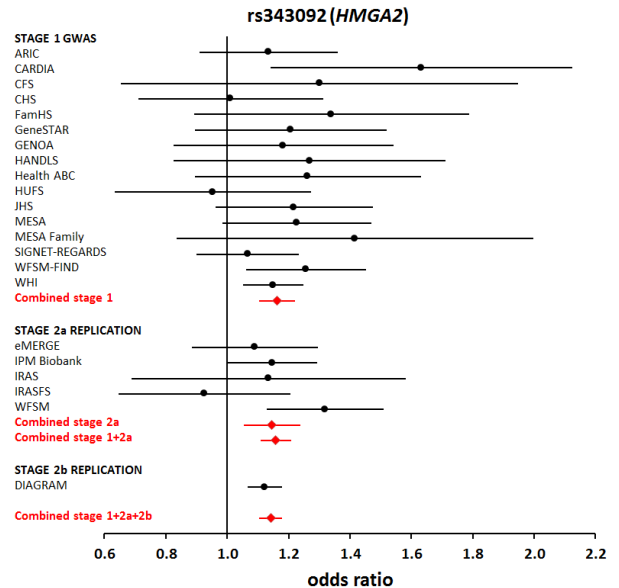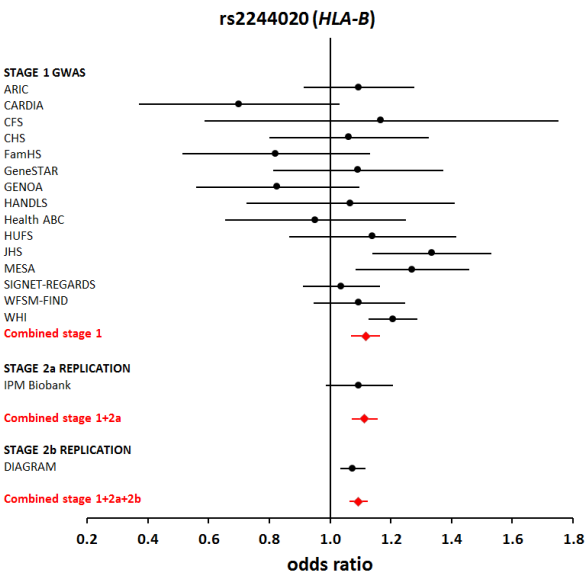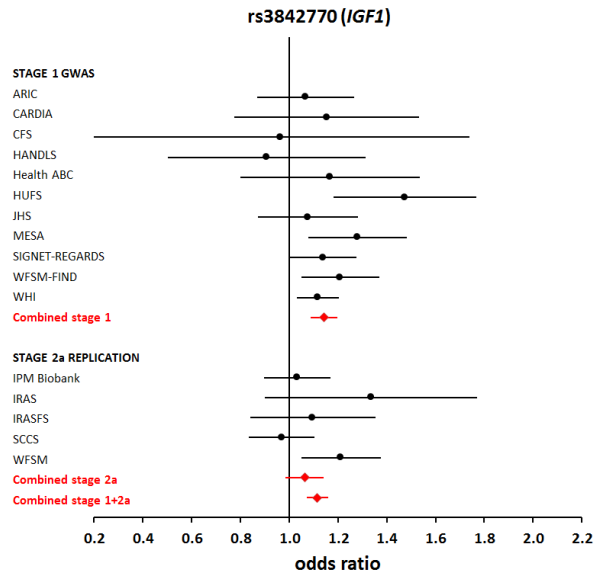

Supplement: Figure S1 — Forest plots of the most strongly associated SNPs at five previously and newly identified T2D loci in African Americans. Odds ratio and 95% CIs are presented for individual studies (black circle and line) and meta-analysis results (red diamond and line). At KCNQ1, two independent associated SNPs are shown. (PDF) [file pgen.1004517.s001.pdf]

**A**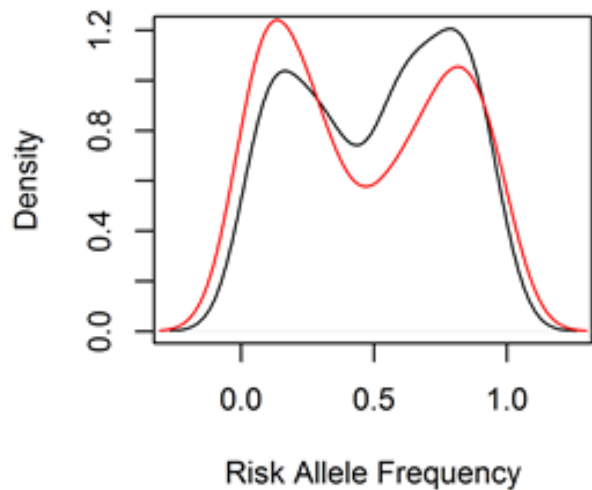**B**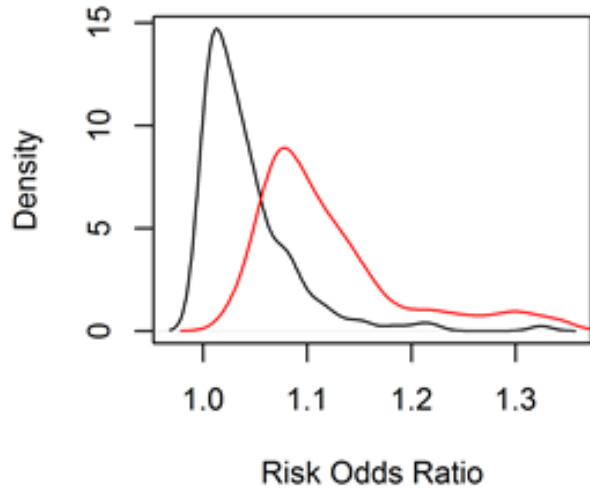

Supplement: Figure S2 — (A) Distributions of risk allele frequencies for the previously reported index SNPs (in black) vs. the MEDIA most strongly associated SNPs (in red) in African Americans from stage 1 meta-analysis. (B) Distributions of odds ratios for risk alleles of the index SNPs (in black) vs. the most strongly associated MEDIA SNPs (in red) in African Americans from stage 1 meta-analysis. (PDF) [file pgen.1004517.s002.pdf]

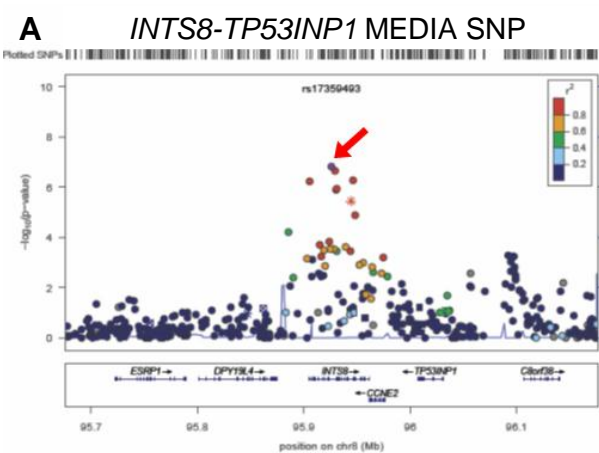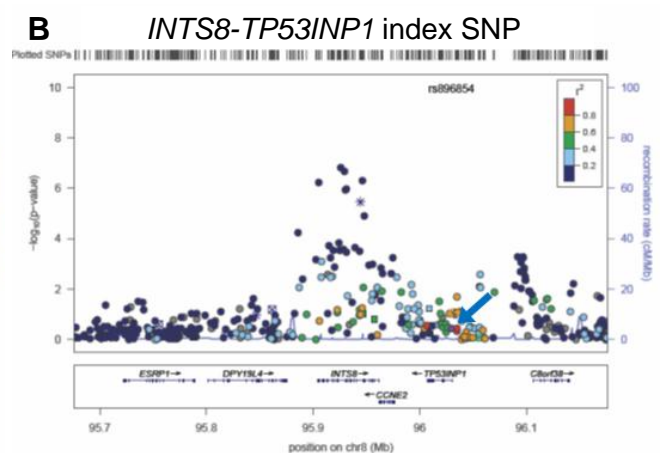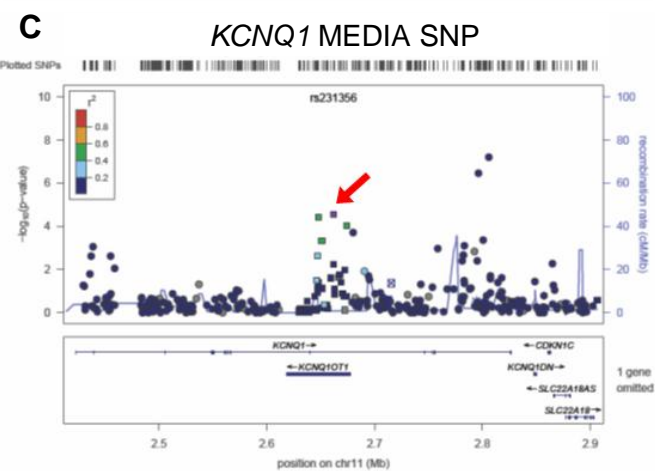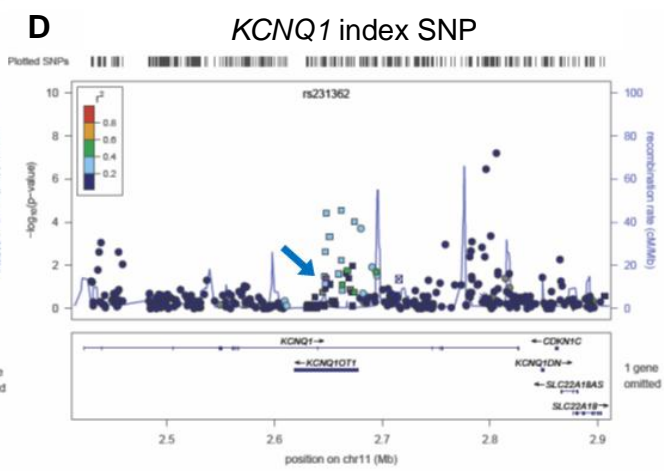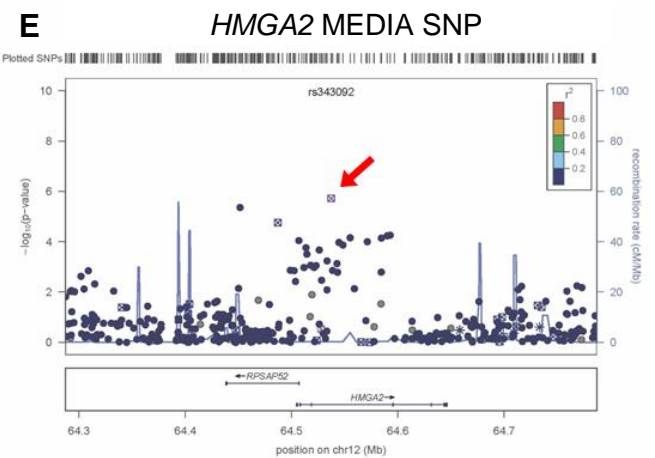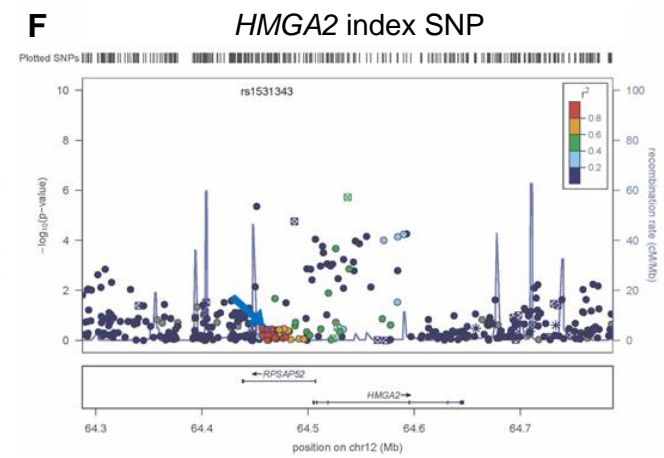

Supplement: Figure S3 — Regional plots of stage 1 meta-analysis association results in African Americans for the most strongly associated SNPs from this study and the index SNPs from previous studies. (A–B) INTS8-TP53INP1 region; (C–D) KCNQ1 region; (E–F) HMGA2 region. (A, C, E) The most strongly associated SNP in MEDIA is denoted by a purple circle and a red arrow with LD colored based on the HapMap 2 YRI data. (B, D, F) The index SNP is denoted by a purple circle and a blue arrow with LD colored based on the HapMap 2 CEU data. (PDF) [file pgen.1004517.s003.pdf]

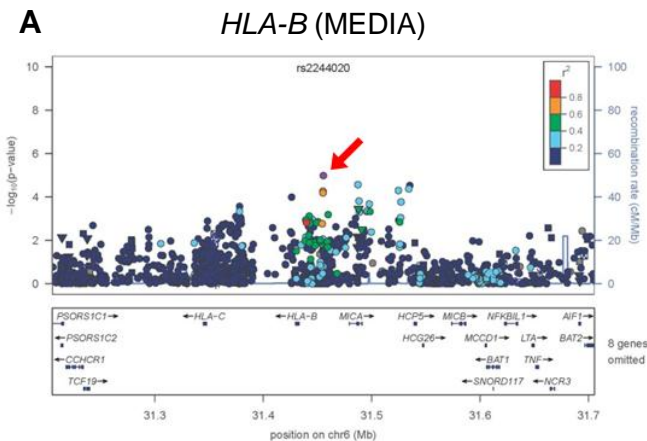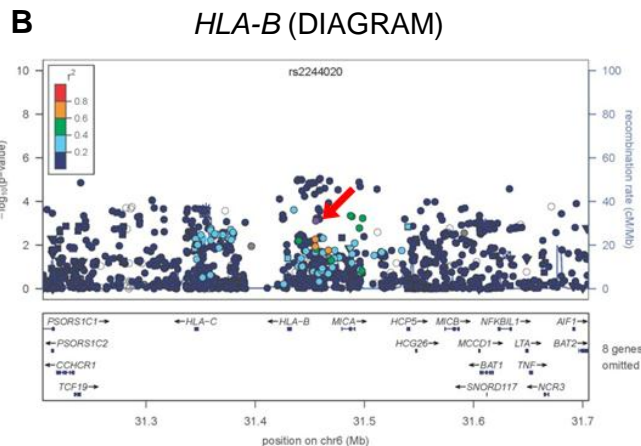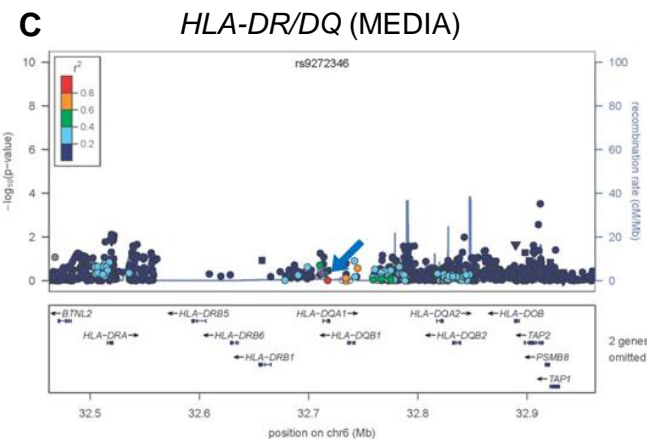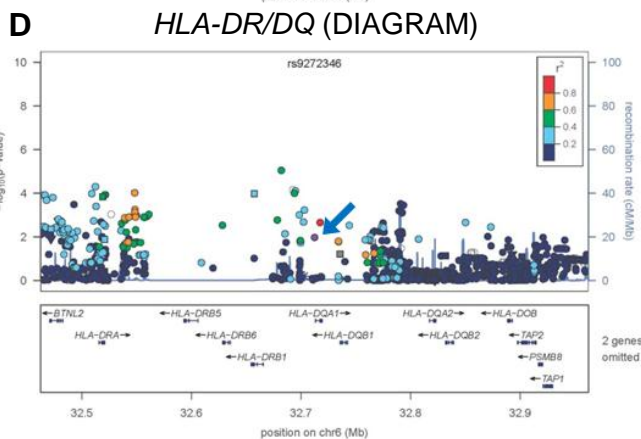

Supplement: Figure S4 — Regional plots of HLA-B and HLA-DQ/DR regions for (A, C) stage 1 meta-analysis association results in African Americans and HapMap 2 YRI LD data, and (B, D) stage 3 DIAGRAMv2 results in Europeans using HapMap 2 CEU LD data. (A, B) The most strongly associated SNP rs2244020 at HLA-B region from this study is denoted by a purple circle and a red arrow. (C, D) The index SNP rs9272346 from Burton PR et al (2007) [65] is denoted by a purple circle and a blue arrow. (PDF) [file pgen.1004517.s004.pdf]
