# Supplementary material for: Meta-Analysis of Genome-Wide Association Studies in African Americans Provides Insights into the Genetic Architecture of Type 2 Diabetes
Source: PLoS Genet. 2014 Aug 7;10(8):e1004517. doi: 10.1371/journal.pgen.1004517 (PMC4125087; doi:10.1371/journal.pgen.1004517)
Supplement: Table S1 — Design of studies in stage 1 GWAS and stage 2a replication in African Americans. (PDF) [file pgen.1004517.s005.pdf]

Table S1. Design of studies in stage 1 GWAS and stage 2a replication in African Americans.

Study abbeviation	Study name	Study design	Sample size in model 1 Case (N)	Control (N)	Total (N)	Sample size in model 2 Case (N)	Control (N)	Total (N)	References (PMID)
**Stage 1: meta-analysis of GWAS**									
ARIC	Atherosclerosis Risk in Communities Study	Population-based, prospective	955	414	1369	942	409	1351	2646917
CARDIA	Coronary Artery Risk Development in Young Adults	Population-based, prospective	94	654	748	90	648	738	3204420
CFS	Cleveland Family Study	Family-based, prospective	81	98	179	81	98	179	7881656
CHS	Cardiovascular Health Study	Population-based, prospective	226	474	700	225	474	699	1669507
FamHS	Family Heart Study	Family-based	146	322	468	146	322	468	8651220
GeneSTAR	Genetic Study of Atherosclerosis Risk	Family-based, prospective	228	620	848	228	620	848	21270820
GENOA	Genetic Epidemiology Network of Arteriopathy	Family-based	293	246	539	293	246	539	11799070
HANDLS	Healthy Aging in Neighborhoods of Diversity across the Life Span Study	Population-based	104	664	768	101	648	749	20828101
Health ABC	Health, Aging, and Body Composition Study	Population-based, prospective	382	232	614	382	232	614	10865790
HUFS	Howard University Family Study	Population-based	183	738	921	183	738	921	19609347
JHS	Jackson Heart Study	Population-based	333	1450	1783	332	1449	1781	16320381
MESA	Multi-Ethnic Study of Atherosclerosis	Population-based, prospective	411	793	1204	397	771	1168	12397006
MESA Family	MESA Family Study	Family-based	69	551	620	69	551	620	12397006
SIGNET-REGARDS	Sea Islands Genetic Network – Reasons for Geographic And Racial Differences in Stroke	Case-control	1141	1243	2384	1134	1240	2374	15990444
WFSM_FIND	Wake Forest School of Medicine, Family Investigation of Nephropathy in Diabetes	Case-control	1674	801	2475	967	796	1763	22238593, 15642484
WHI	Women's Health Initiative	Population-based	1964	6243	8207	1947	6199	8146	9492970
**Stage 2a: *In silico* replication studies**									
eMERGE	Electronic Medical Records and Genomics Network	Population-based	730	830	1560	616	592	1208	22101970 21269473
IPM Biobank	The Charles Bronfman Institute for Personalized Medicine BioBank Program	Population-based	1617	2163	3780	1617	2163	3780	21573225
**Stage 2a: *De novo* replication studies**									
IRAS	Insulin Resistance Atherosclerosis Study	Population-based, prospective	115	164	279	115	163	278	8680609
IRASFS	Insulin Resistance Atherosclerosis Family Study	Family-based, prospective	66	513	579	64	511	575	12684185
SCCS	Southern Community Cohort Study	Population-based	1130	1130	2260	1130	1130	2260	16080667
WFSM	Wake Forest School of Medicine	Case-control	2403	683	3086	2351	676	3027	22238593
