# Supplementary material for: Meta-Analysis of Genome-Wide Association Studies in African Americans Provides Insights into the Genetic Architecture of Type 2 Diabetes
Source: PLoS Genet. 2014 Aug 7;10(8):e1004517. doi: 10.1371/journal.pgen.1004517 (PMC4125087; doi:10.1371/journal.pgen.1004517)
Supplement: Table S2 — Clinical characteristics of study samples in stage 1 GWAS and stage 2a replication studies in African Americans. (PDF) [file pgen.1004517.s006.pdf]

Table S2. Clinical characteristics of study samples in stage 1 GWAS and stage 2a replication studies in African Americans.

Study	N		Age (year)		Age at diagnosis in Case (year)	Male (%)		BMI (kg/m^2)	
	Case	Control	Case	Control		Case	Control	Case	Control
**Stage 1: meta-analysis of GWAS**									
ARIC	955	414	61.3 ± 6.0	59.6 ± 6.3	50.9 ± 9.2	35.8	31.2	32.0 ± 6.7	27.6 ± 5.7
CARDIA	94	654	40.5 ± 3.8	38.2 ± 4.4	35.0 ± 5.5	19.2	38.2	33.8 ± 8.1	29.6 ± 6.8
CFS	81	98	57.8 ± 12.6	43.2 ± 11.8	50.6 ± 11.1	50.6	32.7	38.9 ± 9.4	31.8 ± 7.7
CHS	226	474	77.1 ± 5.6	77.7 ± 5.8	NA	38.1	37.3	30.1 ± 5.5	27.5 ± 5.4
FamHS	146	322	58.1 ± 10.2	50.8 ± 10.6	48.2 ± 10.7	36.3	30.1	34.9 ± 7.7	31.3 ± 7.2
GeneSTAR	228	620	53.2 ± 8.6	46.0 ± 11.5	51.4 ± 8.1	63.6	63.1	35.3 ± 6.9	30.8 ± 7.7
GENOA	293	246	62.3 ± 9.5	60.2 ± 11.2	51.5 ± 10.3	23.2	28	34.2 ± 6.9	29.6 ± 6.8
HANDLS	104	664	51.2 ± 8.7	47.1 ± 9.1	NA	46.5	43.4	34.4 ± 7.9	28.6 ± 7.7
Health ABC	382	232	73.9 ± 3.0	77.6 ± 3.0	NA	45.5	47	29.7 ± 5.1	26.9 ± 5.6
HUFS	183	738	54.1 ± 11.3	46.7 ± 11.0	NA	37.2	46.2	33.9 ± 9.3	29.8 ± 7.9
JHS	333	1450	55.5 ± 10.7	48.6 ± 11.3	46.2 ± 11.0	33.9	38.7	35.2 ± 7.5	31.4 ± 7.5
MESA	411	793	67.6 ± 9.2	65.3 ± 10.5	54.6 ± 10.9	47.2	42.4	31.7 ± 6.2	28.6 ± 5.8
MESA Family	69	551	60.1 ± 8.6	57.1 ± 8.5	48.8 ± 11.0	44.9	36.5	32.6 ± 7.6	29.5 ± 5.7
SIGNET-REGARDS	1141	1243	64.0 ± 8.7	63.1 ± 8.5	NA	36.5	33.5	33.1 ± 7.2	30.1 ± 6.4
WFSM_FIND	1674	801	60.9 ± 10.4	49.5 ± 11.4	37.4 ± 26.9	38.7	44.7	29.9 ± 7.2	30.0 ± 7.1
WHI	1964	6243	62.2 ± 6.8	61.4 ± 7.1	NA	0	0	33.1 ± 6.5	30.4 ± 6.2
**Stage 2a: *In silico* replication studies**									
eMERGE	730	830	57.0 ± 13.9	44.6 ± 15.9	53.4 ± 13.7	38	33.1	35.0 ± 9.0	29.8 ± 7.3
IPM Biobank	1617	2163	59.4 ± 13.1	49.5 ± 14.0	NA	34.8	36.6	32.2 ± 8.3	29.5 ± 7.6
**Stage 2a: *De novo* replication studies**									
IRAS	115	164	56.8 ± 8.0	54.5 ± 8.4	NA	46.1	39	32.1 ± 6	29.3 ± 5.8
IRASFS	66	513	55.3 ± 10.7	41.2 ± 13.5	NA	27.3	42.3	34.7 ± 6.6	29.4 ± 6.6
SCCS	1130	1130	59.7 ± 8.8	57.9 ± 9.4	51.2 ± 12.2	30.7	30.7	33.0 ± 6.9	30.0 ± 6.6
WFSM	2403	683	59.6 ± 11.5	48.4 ± 12.8	43.7±13.5	41	52	31.8 ± 7.7	29.3 ± 7.5

NA, not applicable
