# Supplementary material for: Meta-Analysis of Genome-Wide Association Studies in African Americans Provides Insights into the Genetic Architecture of Type 2 Diabetes
Source: PLoS Genet. 2014 Aug 7;10(8):e1004517. doi: 10.1371/journal.pgen.1004517 (PMC4125087; doi:10.1371/journal.pgen.1004517)
Supplement: Table S3 — Genotyping methods, quality controls, imputation and statistical analysis in stage 1 GWAS and stage 2a replication studies in African Americans. (PDF) [file pgen.1004517.s007.pdf]

Table S3. Genotyping methods, quality controls, imputation and statistical analysis in stage 1 GWAS and stage 2a replication studies in African Americans.

	Genotyping					Imputation				Association analysis					Sample QC	
**Study**	**Platform**	**Call rate**	**MAF**	**HWE *P* value**	**Additional exclusion criteria**	**Method**	**Reference panel**	**MAF**	**Imputation quality**	**No. of SNPs**	**λ_{GC}**	**Method**	**Covariates**	**SNP exclusion**	**Call rate**	**Cryptic relatedness**
**Stage 1: meta-analysis of GWAS**																
ARIC	Affymetrix 6.0	≥ 0.95	≥ 0.01	≥ 1E-4		MACH v1.0.16	HapMap II rel#22 CEU, YRI	≥ 0.01	Rsq ≥ 0.5	2702732	1.022	PLINK, MACH2 DAT	age, gender, center, PC1	MAC < 10; |beta| > 10; standard error > 10	≥ 0.95	
CARDIA	Affymetrix 6.0	≥ 0.95	≥ 0.01	≥ 1E-4		MACH v1.0.16	HapMap II rel#22 CEU, YRI	≥ 0.01	Rsq ≥ 0.5	2718487	1.02	PLINK, MACH2 DAT	age, gender, center, PC1	MAC < 10; |beta| > 10; standard error > 10	≥ 0.95	
CFS	Affymetrix 6.0	≥ 0.95	≥ 0.01	≥ 1E-4		MACH v1.0.16	HapMap II rel#22 CEU, YRI	≥ 0.01	Rsq ≥ 0.5	2332067	1.084	GWAF	age, gender, center, PC1	MAC < 10; |beta| > 10; standard error > 10	≥ 0.95	
CHS	Illumina HumanOmni1-Quad_v1	≥ 0.97	≥ 0.01	≥ 1E-4	≥ 1 duplicate error or Mendelian inconsistency in HapMap controls; heterozygote frequency = 0	BEAGLE v.3.2.1	HapMap II CEU, YRI; HapMap III CEU, YRI and ASW	≥ 0.01	oevar ≥ 0.5	2494079	1.046	R	age, gender, center, PC1-PC10	MAC < 10; |beta| > 10; standard error > 10	≥ 0.95	
FamHS	Illumina DUOv3, Illumina 1M	≥ 0.99	≥ 0.01	≥ 1E-4	variance < 0.02	MACH v1.0.16	HapMap II rel#21 CEU, YRI	≥ 0.01	Rsq ≥ 0.5	2171412	1.029	R	age, gender, center, PC1-PC10	MAC < 10; |beta| > 10; standard error > 10	≥ 0.95	
GeneSTAR	Illumina 1M	≥ 0.95	≥ 0.01	≥ 1E-4	>5% Mendelian inconsistency rate	MACH v1.0.16	HapMap II rel#21 CEU, YRI	≥ 0.01	Rsq ≥ 0.5	2200196	1.031	R (GEE,GEEGLM)	age, gender, PC1, PC6	MAC < 10; |beta| > 10; standard error > 10	≥ 0.95	
GENOA	Affymetrix 6.0, Illumina 1M	≥ 0.95	≥ 0.05	≥ 1E-4		MACH v1.0.16	HapMap II rel#24 CEU, YRI	≥ 0.01	Rsq ≥ 0.5	1978694	1.077	R (GEE)	age, gender, PC1-PC2	MAC < 10; |beta| > 10; standard error > 10	≥ 0.95	
HANDLS	Illumina 1M, Illumina 1M-Duo	≥ 0.95	≥ 0.1	≥ 1E-4		MACH v1.0.16	HapMap II rel#22 CEU, YRI	≥ 0.01	Rsq ≥ 0.5	2832540	1.023	MACH2 DAT	age, gender, PC1-PC10	MAC < 10; |beta| > 10; standard error > 10	≥ 0.95	removed
Health ABC	Illumina 1M-Duo	≥ 0.97	≥ 0.01	≥ 1E-4		MACH v1.0.16	HapMap II rel#22 CEU, YRI	≥ 0.01	Rsq ≥ 0.5	2936968	1.033	R	age, gender, PC1	MAC < 10; |beta| > 10; standard error > 10	≥ 0.95	removed
HUFS	Affymetrix 6.0	≥ 0.95	≥ 0.01	≥ 1E-3		MACH v1.0.16	HapMap II + III rel#27 CEU, YRI	≥ 0.01	Rsq ≥ 0.5	2078910	1.016	PLINK	age, gender, PC1	MAC < 10; |beta| > 10; standard error > 10	≥ 0.95	
JHS	Affymetrix 6.0	≥ 0.95	≥ 0.01	≥ 1E-4		MACH v1.0.16	HapMap II rel#22 CEU, YRI	≥ 0.01	Rsq ≥ 0.5	2730908	1.079	PLINK, MACH2 DAT	age, gender, center, PC1	MAC < 10; |beta| > 10; standard error > 10	≥ 0.95	
MESA	Affymetrix 6.0	≥ 0.95	≥ 0.01	≥ 1E-4		MACH v1.0.16	HapMap II rel#22 CEU, YRI	≥ 0.01	Rsq ≥ 0.5	2743725	1.009	PLINK, MACH2 DAT	age, gender, center, PC1	MAC < 10; |beta| > 10; standard error > 10	≥ 0.95	
MESA Family	Affymetrix 6.0	≥ 0.95	≥ 0.01	≥ 1E-4	Heterozygosity < 53%	IMPUTE2	HapMap I & II rel#22 all populations	≥ 0.01	proper_info ≥ 0.5	2466511	1.06	GWAF	age, gender, center, PC1-PC2	MAC < 10; |beta| > 10; standard error > 10	≥ 0.95	

SIGNET-REGARDS	Affymetrix 6.0	≥ 0.95	≥ 0.05	≥ 1E-4		MACH v1.0.16	HapMap II rel#22 CEU, YRI	≥ 0.01	Rsq ≥ 0.5	2484637	1.009	R	age, gender, PC1-PC10	MAC < 10; |beta| > 10; standard error > 10	≥ 0.90	
WFSM_ FIND	Affymetrix 6.0	≥ 0.95	≥ 0.01	≥ 1E-4	case-control differential missingness p > 0.001; homozygous minor allele count < 10	IMPUTE2	HapMap II & III all populations	≥ 0.01	proper_info ≥ 0.5	2039806	1.052	SNPGWA, SNPTEST	age, gender, PC1-PC2	MAC < 10; |beta| > 10; standard error > 10	≥ 0.95	
WHI	Affymetrix 6.0	≥ 0.95	≥ 0.01	≥ 1E-4	concordance rate < 0.98	MACH v1.0.16	HapMap II CEU, YRI	≥ 0.01	Rsq ≥ 0.5	2373847	1.021	SNPGWA, ProbABEL	age, geographic region, PC1-PC3	MAC < 10; |beta| > 10; standard error > 10	≥ 0.95	removed
Meta-analysis										2579389	1.056					
**Stage 2a: *In silico* replication studies**																
eMERGE	Illumina 1M	≥ 0.98	≥ 0.01	≥ 1E-4	≥ 1 duplicate error or Mendelian inconsistency in HapMap controls	BEAGLE v.3.2.1	HapMap II rel#28 CEU, YRI	≥ 0.01	oevar ≥ 0.5			SNPTEST	age, gender, PC1-PC2	MAC < 10; |beta| > 10; standard error > 10	≥ 0.98	removed
IPM Biobank	Affymetrix 6.0, Illumina Human Omni Express Exome	≥ 0.95	≥ 0.01	≥ 1E-3		MACH v1.0.16, IMPUTE2	HapMap II rel#22 CEU, YRI	≥ 0.01	Rsq or proper_info ≥ 0.5			SNPTEST, MACH2DAT	age, gender, PC1	MAC < 10; |beta| > 10; standard error > 10	≥ 0.95	removed
**Stage 2a: *De novo* replication studies**																
IRAS	Sequenom	≥ 0.95		≥ 1E-4								PLINK	age, gender, PC1	|beta| > 10; standard error > 10	≥ 0.95	
IRASFS	Sequenom	≥ 0.95		≥ 1E-4								SOLAR	age, gender, PC1	|beta| > 10; standard error > 10	≥ 0.95	
SCCS	Sequenom	≥ 0.95		≥ 1E-4								SAS	age, gender, PC1-PC5	|beta| > 10; standard error > 10	≥ 0.95	
WFSM	Sequenom	≥ 0.95		≥ 1E-4								PLINK	age, gender, PC1	|beta| > 10; standard error > 10	≥ 0.95	

Abbreviations: MAF, minor allele frequency; BMI, body mass index; MAC, minor allele count
