# Supplementary material for: Meta-Analysis of Genome-Wide Association Studies in African Americans Provides Insights into the Genetic Architecture of Type 2 Diabetes
Source: PLoS Genet. 2014 Aug 7;10(8):e1004517. doi: 10.1371/journal.pgen.1004517 (PMC4125087; doi:10.1371/journal.pgen.1004517)
Supplement: Table S4 — SNPs with P value≤1×10^−5^ from stage 1 GWAS meta-analysis (BMI unadjusted) selected for stage 2 in silico and de novo replication in African Americans and in silico replication in individuals of European ancestry from DIAGRAMv2. (PDF) [file pgen.1004517.s008.pdf]

**Table S4.** SNPs with $P$ value < $1\times10^{-5}$ from stage 1 GWAS meta-analysis selected for stage 2 *in silico* and *de novo* replication in African Americans and *in silico* replication in individuals of European ancestry from DIAGRAMv2.

| | | | | | Stage 1 African American GWAS meta-analysis | | | | | Stage 2a African American replication | | | | Stage 1+2a African American GWAS meta-analysis + replication | | | | Stage 2b European replication | | | Stage 1+2a+2b African American + European GWAS meta-analysis + replication | | | |
|---|---|---|---|---|---|---|---|---|---|---|---|---|---|---|---|---|---|---|---|---|---|---|---|---|
| **Chr** | **Position** | **SNP** | **Locus** | **Alleles[a]** | **RAF** | **OR (95% CI)[b]** | ***P*** | **$P_{het}$[c]** | **N** | **OR (95% CI)[b]** | ***P*** | **$P_{het}$[c]** | **N** | **OR (95% CI)[b]** | ***P*** | **$P_{het}$[c]** | **N** | **OR (95% CI)[b]** | ***P*** | **$N_{eff}$** | **OR (95% CI)[b]** | ***P*** | **$P_{het}$[c]** | **N** |
| 1 | 239254905 | rs679992 | | T/C | 0.50 | 1.12(1.07-1.17) | 1.24E-06 | 6.83E-02 | 22902 | 1.04(0.97-1.11) | 2.36E-01 | 6.82E-01 | 9178 | 1.09(1.05-1.13) | 1.13E-06 | 7.38E-02 | 32080 | 0.99(0.95-1.03) | 7.38E-01 | 22570 | 1.08(1.03-1.13) | 3.50E-03 | 6.55E-04 | 54650 |
| 2 | 30921523 | rs12613372 | *CAPN13-GALNT14* | G/C | 0.07 | 1.26(1.16-1.38) | 2.21E-07 | 9.44E-02 | 21925 | 1.02(0.77-1.34) | 9.17E-01 | 1.00E+00 | 3780 | 1.24(1.14-1.34) | 2.35E-07 | 1.47E-01 | 25705 | 1.03(0.97-1.08) | 3.49E-01 | 22570 | 1.16(1.06-1.28) | 1.26E-02 | 2.54E-04 | 48275 |
| 3 | 169527567 | rs9290337 | *LOC389174* | G/A | 0.27 | 1.16(1.09-1.24) | 6.38E-06 | 7.30E-01 | 14567 | 1.03(0.96-1.11) | 3.88E-01 | 1.66E-01 | 9205 | 1.11(1.05-1.16) | 3.86E-05 | 1.81E-02 | 23772 | | | | 1.11(1.05-1.16) | 3.86E-05 | 1.00E+00 | 23772 |
| 3 | 178198612 | rs1905499 | *TBL1XR1* | G/T | 0.59 | 1.11(1.06-1.16) | 8.16E-06 | 5.94E-01 | 23818 | 1.02(0.96-1.09) | 5.45E-01 | 9.71E-01 | 9185 | 1.08(1.04-1.12) | 2.34E-05 | 4.30E-02 | 33003 | 1(0.96-1.05) | 9.02E-01 | 22570 | 1.05(1.02-1.08) | 1.04E-03 | 9.63E-03 | 55573 |
| 3 | 178203474 | rs1905496 | *TBL1XR1* | A/G | 0.59 | 1.11(1.06-1.16) | 7.55E-06 | 6.18E-01 | 23740 | 0.97(0.91-1.04) | 4.64E-01 | 5.51E-02 | 7553 | 1.07(1.03-1.11) | 3.75E-04 | 1.84E-03 | 31293 | 1(0.96-1.04) | 9.47E-01 | 22570 | 1.04(1.01-1.07) | 6.68E-03 | 2.80E-02 | 53863 |
| 4 | 931518 | rs2290402 | *TMEM175* | T/C | 0.05 | 1.33(1.18-1.51) | 6.71E-06 | 7.61E-01 | 18865 | 0.95(0.82-1.11) | 5.26E-01 | 9.04E-01 | 10470 | 1.16(1.06-1.28) | 1.22E-02 | 7.25E-04 | 29335 | 1.06(1-1.13) | 5.89E-02 | 22570 | 1.14(1.05-1.23) | 1.07E-02 | 1.11E-01 | 51905 |
| 6 | 31455430 | rs2244020 | *HLA-B* | G/A | 0.69 | 1.12(1.06-1.17) | 1.02E-05 | 2.11E-02 | 23098 | 1.1(0.98-1.22) | 1.01E-01 | 1.00E+00 | 3780 | 1.11(1.07-1.16) | 1.14E-06 | 7.57E-01 | 26878 | 1.07(1.03-1.12) | 7.67E-04 | 22570 | 1.09(1.06-1.13) | 6.57E-09 | 2.55E-01 | 49448 |
| 6 | 121716077 | rs4946566 | *GJA1* | G/C | 0.03 | 1.38(1.2-1.59) | 6.29E-06 | 6.71E-01 | 18041 | 1.03(0.83-1.28) | 7.68E-01 | 3.96E-01 | 9226 | 1.27(1.14-1.43) | 3.63E-05 | 2.52E-02 | 27267 | 1(0.93-1.09) | 9.34E-01 | 22570 | 1.08(1.01-1.16) | 1.64E-02 | 1.19E-03 | 49837 |
| 6 | 169234624 | rs12175557 | *SMOC2* | A/G | 0.02 | 1.56(1.3-1.87) | 1.74E-06 | 9.81E-01 | 18783 | 1(0.75-1.34) | 9.76E-01 | 9.46E-01 | 9213 | 1.39(1.19-1.61) | 1.87E-05 | 1.11E-02 | 27996 | 1(0.9-1.12) | 9.30E-01 | 10934 | 1.12(1.03-1.23) | 9.40E-03 | 1.10E-03 | 38930 |
| 7 | 43286956 | rs9648079 | *HECW1* | G/A | 0.74 | 1.15(1.09-1.22) | 1.71E-06 | 9.78E-01 | 21321 | 0.97(0.87-1.07) | 4.87E-01 | 6.01E-01 | 5340 | 1.1(1.05-1.16) | 6.23E-05 | 2.43E-03 | 26661 | 0.99(0.94-1.05) | 8.40E-01 | 22570 | 1.05(1.02-1.09) | 4.90E-03 | 6.02E-03 | 49231 |
| 7 | 43287119 | rs10231619 | *HECW1* | T/C | 0.74 | 1.15(1.08-1.21) | 1.92E-06 | 9.80E-01 | 22353 | 1(0.87-1.15) | 9.84E-01 | 1.00E+00 | 3780 | 1.13(1.07-1.18) | 3.87E-06 | 7.32E-02 | 26133 | 0.99(0.94-1.05) | 8.12E-01 | 22570 | 1.07(1.03-1.11) | 8.94E-04 | 1.73E-03 | 48703 |
| 7 | 43292722 | rs4724195 | *HECW1* | T/C | 0.71 | 1.13(1.07-1.19) | 3.82E-06 | 7.68E-01 | 23737 | 1(0.93-1.07) | 9.56E-01 | 8.81E-01 | 11400 | 1.08(1.04-1.12) | 1.33E-04 | 3.48E-03 | 35137 | 0.99(0.94-1.05) | 7.76E-01 | 22570 | 1.05(1.01-1.08) | 4.12E-03 | 1.44E-02 | 57707 |
| 7 | 43293128 | rs10272268 | *HECW1* | C/T | 0.71 | 1.13(1.07-1.18) | 6.90E-06 | 6.59E-01 | 23796 | 1(0.93-1.08) | 9.28E-01 | 6.41E-01 | 9165 | 1.09(1.04-1.13) | 7.78E-05 | 1.31E-02 | 32961 | 0.99(0.94-1.05) | 8.20E-01 | 22570 | 1.05(1.02-1.09) | 3.02E-03 | 1.09E-02 | 55531 |
| 7 | 43293209 | rs10951720 | *HECW1* | G/C | 0.71 | 1.12(1.07-1.18) | 7.88E-06 | 5.12E-01 | 23818 | 0.99(0.9-1.09) | 8.74E-01 | 7.15E-01 | 5340 | 1.09(1.05-1.14) | 4.96E-05 | 2.33E-02 | 29158 | 0.98(0.93-1.04) | 5.83E-01 | 22570 | 1.05(1.02-1.09) | 4.63E-03 | 4.02E-03 | 51728 |
| 7 | 43293220 | rs10951721 | *HECW1* | C/T | 0.71 | 1.13(1.07-1.18) | 5.91E-06 | 5.48E-01 | 23792 | 1(0.92-1.07) | 9.10E-01 | 7.65E-01 | 9175 | 1.08(1.04-1.13) | 1.05E-04 | 7.87E-03 | 32967 | 0.98(0.93-1.04) | 5.83E-01 | 22570 | 1.05(1.01-1.08) | 5.54E-03 | 7.24E-03 | 55537 |
| 7 | 43294434 | rs10242062 | *HECW1* | T/C | 0.69 | 1.13(1.07-1.19) | 2.13E-06 | 6.07E-01 | 23820 | 0.98(0.9-1.06) | 6.32E-01 | 7.69E-01 | 7625 | 1.09(1.04-1.13) | 5.80E-05 | 3.75E-03 | 31445 | 0.99(0.94-1.05) | 7.84E-01 | 22570 | 1.05(1.02-1.09) | 2.75E-03 | 9.20E-03 | 54015 |
| 7 | 43296714 | rs7794383 | *HECW1* | T/C | 0.74 | 1.14(1.08- | 7.10E- | 9.80E- | 21340 | 0.97(0.87- | 4.95E- | 6.04E- | 5340 | 1.1(1.05- | 1.56E- | 4.49E- | 26680 | 0.99(0.94- | 8.02E- | 22570 | 1.05(1.01- | 8.51E- | 8.86E- | 49250 |

| | | | | | | | | | | | | | | | | | | | | | | | | | |
|---|---|---|---|---|---|---|---|---|---|---|---|---|---|---|---|---|---|---|---|---|---|---|---|---|---|
| | | | | | | 1.21) | 06 | 01 | | 1.07) | 01 | 01 | | 1.15) | 04 | 03 | | 1.05) | 01 | | 1.09) | 03 | 03 | |
| 8 | 68775944 | rs7003257 | *CPA6* | T/C | 0.73 | 1.15(1.09-1.22) | 1.17E-06 | 7.94E-01 | 21351 | 1.03(0.96-1.1) | 4.52E-01 | 1.51E-01 | 9839 | 1.1(1.06-1.15) | 8.79E-06 | 1.20E-02 | 31190 | 1.04(0.95-1.14) | 3.77E-01 | 22570 | 1.09(1.05-1.13) | 1.04E-05 | 3.13E-01 | 53760 |
| 8 | 95905316 | rs11786088 | *INTS8* | T/C | 0.22 | 1.15(1.09-1.21) | 5.83E-07 | 8.75E-01 | 22888 | 1.04(0.94-1.15) | 4.68E-01 | 7.02E-02 | 5340 | 1.13(1.07-1.18) | 6.35E-07 | 9.04E-02 | 28228 | 1.06(1-1.11) | 3.36E-02 | 22570 | 1.09(1.06-1.13) | 3.72E-07 | 7.17E-02 | 50798 |
| 8 | 95926087 | rs17359493 | *INTS8* | G/A | 0.22 | 1.15(1.09-1.22) | 1.49E-07 | 8.97E-01 | 23799 | 1.04(0.94-1.16) | 4.17E-01 | 7.93E-02 | 5340 | 1.13(1.08-1.18) | 1.39E-07 | 8.39E-02 | 29139 | 1.06(1-1.11) | 3.20E-02 | 22570 | 1.1(1.06-1.13) | 1.01E-07 | 5.47E-02 | 51709 |
| 8 | 95929347 | rs16917079 | *INTS8* | G/A | 0.21 | 1.15(1.09-1.21) | 2.10E-07 | 9.24E-01 | 23807 | 1.01(0.94-1.08) | 8.02E-01 | 5.37E-02 | 11388 | 1.1(1.06-1.15) | 5.59E-06 | 2.94E-03 | 35195 | 1.06(1-1.11) | 3.13E-02 | 22570 | 1.08(1.05-1.12) | 1.15E-06 | 2.41E-01 | 57765 |
| 8 | 95930461 | rs16917081 | *INTS8* | G/A | 0.25 | 1.13(1.08-1.19) | 1.18E-06 | 5.54E-01 | 23768 | 0.98(0.89-1.07) | 6.53E-01 | 7.71E-01 | 5340 | 1.1(1.05-1.15) | 1.62E-05 | 5.98E-03 | 29108 | 1.05(1.01-1.1) | 2.91E-02 | 22570 | 1.08(1.04-1.11) | 3.34E-06 | 2.22E-01 | 51678 |
| 8 | 95931079 | rs6986418 | *INTS8* | G/A | 0.25 | 1.13(1.08-1.19) | 1.09E-06 | 6.99E-01 | 23824 | 1(0.93-1.08) | 9.48E-01 | 3.69E-01 | 9222 | 1.09(1.05-1.14) | 2.14E-05 | 4.63E-03 | 33046 | 1.05(1.01-1.1) | 2.85E-02 | 22570 | 1.08(1.04-1.11) | 3.45E-06 | 2.96E-01 | 55616 |
| 8 | 95944398 | rs11782617 | *INTS8* | T/G | 0.21 | 1.14(1.08-1.2) | 3.56E-06 | 9.15E-01 | 23671 | 1.04(0.95-1.12) | 4.01E-01 | 1.71E-01 | 9211 | 1.11(1.06-1.16) | 6.07E-06 | 5.79E-02 | 32882 | 1.06(1.01-1.11) | 2.82E-02 | 22570 | 1.08(1.05-1.12) | 1.28E-06 | 1.98E-01 | 55452 |
| 8 | 95946461 | rs12056517 | *INTS8* | T/C | 0.22 | 1.14(1.09-1.21) | 4.99E-07 | 8.33E-01 | 23791 | 1.04(0.94-1.15) | 4.20E-01 | 7.89E-02 | 5340 | 1.12(1.07-1.18) | 4.52E-07 | 1.07E-01 | 29131 | 1.06(1.01-1.11) | 3.09E-02 | 22570 | 1.09(1.06-1.13) | 2.25E-07 | 7.90E-02 | 51701 |
| 8 | 135126458 | rs2168707 | *ZFAT* | G/A | 0.11 | 1.24(1.13-1.36) | 4.55E-06 | 3.20E-01 | 14536 | 0.96(0.86-1.07) | 4.52E-01 | 9.92E-01 | 9187 | 1.13(1.04-1.22) | 1.22E-02 | 2.82E-04 | 23723 | | | | 1.13(1.04-1.22) | 1.22E-02 | 1.00E+00 | 23723 |
| 9 | 129871150 | rs10739706 | *SLC25A25* | G/A | 0.62 | 1.12(1.07-1.17) | 3.64E-06 | 3.85E-01 | 23776 | 1.02(0.96-1.08) | 5.81E-01 | 8.28E-01 | 11417 | 1.08(1.04-1.12) | 2.82E-05 | 1.49E-02 | 35193 | 1.01(0.96-1.06) | 7.38E-01 | 22570 | 1.06(1.03-1.09) | 2.59E-04 | 4.42E-02 | 57763 |
| 9 | 130111543 | rs2231645 | *TRUB2* | A/T | 0.66 | 1.15(1.08-1.23) | 6.36E-06 | 4.00E-01 | 16238 | 0.99(0.86-1.13) | 8.38E-01 | 8.74E-01 | 5339 | 1.13(1.07-1.19) | 2.38E-05 | 3.69E-02 | 21577 | 1.01(0.93-1.09) | 8.51E-01 | 13971 | 1.08(1.04-1.13) | 3.95E-04 | 2.48E-02 | 35548 |
| 9 | 130111544 | rs2231644 | *TRUB2* | C/T | 0.66 | 1.15(1.09-1.23) | 5.82E-06 | 3.78E-01 | 16939 | 0.98(0.85-1.12) | 7.17E-01 | 6.84E-01 | 5339 | 1.12(1.06-1.19) | 2.83E-05 | 2.52E-02 | 22278 | 1.01(0.93-1.09) | 8.52E-01 | 13971 | 1.08(1.04-1.13) | 4.44E-04 | 2.67E-02 | 36249 |
| 10 | 114744078 | rs7901695 | *TCF7L2* | C/T | 0.46 | 1.2(1.14-1.25) | 3.18E-15 | 2.28E-01 | 23817 | 1.26(1.16-1.37) | 1.33E-07 | 1.22E-01 | 5340 | 1.21(1.16-1.26) | 2.03E-22 | 2.97E-01 | 29157 | 1.36(1.3-1.42) | 1.38E-41 | 22570 | 1.24(1.17-1.30) | 1.11E-14 | 1.56E-04 | 51727 |
| 10 | 114745486 | rs4132115 | *TCF7L2* | T/G | 0.15 | 1.23(1.13-1.34) | 4.39E-04 | 5.68E-04 | 23609 | 1.37(1.22-1.54) | 1.00E-07 | 1.15E-01 | 5340 | 1.23(1.17-1.3) | 4.47E-15 | 4.66E-02 | 28949 | | | | 1.26(1.16-1.36) | 1.13E-05 | 1.00E+00 | 28949 |
| 10 | 114746031 | rs4506565 | *TCF7L2* | T/A | 0.45 | 1.15(1.07-1.23) | 1.70E-03 | 3.97E-04 | 23114 | 1.26(1.16-1.37) | 1.22E-07 | 1.24E-01 | 5340 | 1.17(1.12-1.21) | 8.14E-15 | 4.66E-02 | 28454 | 1.36(1.31-1.43) | 1.68E-44 | 22570 | 1.19(1.11-1.26) | 1.27E-05 | 3.36E-07 | 51024 |
| 10 | 114746248 | rs7068741 | *TCF7L2* | T/C | 0.15 | 1.26(1.18-1.35) | 1.71E-12 | 3.12E-01 | 21337 | 1.36(1.21-1.53) | 2.84E-07 | 9.56E-02 | 5340 | 1.28(1.22-1.36) | 4.92E-19 | 2.69E-01 | 26677 | | | | 1.28(1.22-1.36) | 4.92E-19 | 1.00E+00 | 26677 |
| 10 | 114746275 | rs7069007 | *TCF7L2* | C/G | 0.11 | 1.28(1.2-1.38) | 2.28E-12 | 2.40E-01 | 23820 | 1.31(1.11-1.54) | 1.61E-03 | 1.00E+00 | 3780 | 1.29(1.21-1.37) | 1.26E-15 | 8.57E-01 | 27600 | | | | 1.29(1.21-1.37) | 1.26E-15 | 1.00E+00 | 27600 |
| 10 | 114748339 | rs7903146 | *TCF7L2* | T/C | 0.30 | 1.32(1.25-1.4) | 6.62E-24 | 1.81E-01 | 20431 | 1.34(1.26-1.43) | 8.38E-20 | 6.01E-03 | 10909 | 1.33(1.28-1.39) | 4.78E-44 | 7.34E-01 | 31340 | 1.4(1.34-1.46) | 2.21E-51 | 22570 | 1.36(1.32-1.4) | 4.15E-94 | 1.16E-01 | 53910 |
| 10 | 114749435 | rs11196187 | *TCF7L2* | A/G | 0.07 | 1.35(1.2-1.52) | 5.45E-07 | 2.79E-01 | 15960 | 1.15(0.93-1.43) | 2.06E-01 | 1.00E+00 | 3780 | 1.31(1.18-1.45) | 1.60E-07 | 1.84E-01 | 19740 | 1.24(1.13-1.37) | 3.96E-06 | 18677 | 1.27(1.19-1.36) | 3.69E-12 | 5.26E-01 | 38417 |
| 10 | 114751709 | rs12098651 | *TCF7L2* | A/G | 0.19 | 1.23(1.15-1.31) | 8.17E-10 | 7.51E-01 | 20431 | 1.29(1.12-1.48) | 2.88E-04 | 1.00E+00 | 3780 | 1.24(1.17-1.31) | 2.10E-13 | 5.30E-01 | 24211 | | | | 1.24(1.17-1.31) | 2.10E-13 | 1.00E+00 | 24211 |

| | | | | | | | | | | | | | | | | | | | | | | | | |
|---|---|---|---|---|---|---|---|---|---|---|---|---|---|---|---|---|---|---|---|---|---|---|---|---|
| 10 | 114759396 | rs4319449 | *TCF7L2* | G/T | 0.09 | 1.28(1.17-1.41) | 2.46E-07 | 3.35E-01 | 19892 | 1.32(1.09-1.59) | 4.54E-03 | 1.00E+00 | 3780 | 1.29(1.19-1.4) | 1.11E-09 | 8.11E-01 | 23672 | | | | 1.29(1.19-1.4) | 1.11E-09 | 1.00E+00 | 23672 |
| 10 | 114789081 | rs7081912 | *TCF7L2* | A/G | 0.16 | 1.18(1.1-1.26) | 1.70E-06 | 7.01E-01 | 20431 | 1.2(1.05-1.38) | 8.67E-03 | 1.00E+00 | 3780 | 1.18(1.12-1.26) | 1.67E-08 | 8.08E-01 | 24211 | | | | 1.18(1.12-1.26) | 1.67E-08 | 1.00E+00 | 24211 |
| 10 | 114807226 | rs7907632 | *TCF7L2* | G/A | 0.16 | 1.18(1.11-1.27) | 1.49E-06 | 8.13E-01 | 20431 | 1.28(1.15-1.44) | 1.56E-05 | 2.16E-02 | 5340 | 1.21(1.14-1.28) | 6.60E-11 | 2.32E-01 | 25771 | | | | 1.21(1.14-1.28) | 6.60E-11 | 1.00E+00 | 25771 |
| 11 | 2135246 | rs3842770 | *INS-IGF2* | A/G | 0.23 | 1.14(1.08-1.21) | 3.41E-06 | 8.03E-01 | 21191 | 1.06(0.98-1.15) | 1.19E-01 | 2.33E-01 | 9423 | 1.12(1.07-1.17) | 1.20E-06 | 1.35E-01 | 30614 | | | | 1.12(1.07-1.17) | 1.20E-06 | 1.00E+00 | 30614 |
| 11 | 2160864 | rs11043007 | *TH-ASCL2* | G/T | 0.81 | 1.22(1.12-1.33) | 1.92E-06 | 5.42E-01 | 20431 | 1.13(1.02-1.25) | 2.48E-02 | 3.45E-01 | 7202 | 1.19(1.11-1.26) | 1.26E-07 | 2.42E-01 | 27633 | 1.02(0.96-1.08) | 4.98E-01 | 13971 | 1.14(1.06-1.23) | 7.05E-04 | 6.18E-04 | 41604 |
| 11 | 2162468 | rs7396243 | *TH-ASCL2* | G/T | 0.80 | 1.21(1.12-1.32) | 2.95E-06 | 4.72E-01 | 20431 | 1.05(0.9-1.21) | 5.49E-01 | 1.00E+00 | 3780 | 1.17(1.1-1.26) | 5.41E-06 | 7.80E-02 | 24211 | 1.02(0.97-1.08) | 4.56E-01 | 13971 | 1.08(1.03-1.13) | 5.54E-04 | 3.00E-03 | 38182 |
| 11 | 2163785 | rs11564703 | *TH-ASCL2* | G/A | 0.80 | 1.22(1.13-1.33) | 1.67E-06 | 4.17E-01 | 20431 | 1.09(0.99-1.19) | 7.01E-02 | 3.56E-01 | 9357 | 1.16(1.1-1.24) | 7.59E-07 | 6.46E-02 | 29788 | 1.02(0.97-1.08) | 4.42E-01 | 13971 | 1.09(1.04-1.13) | 8.02E-05 | 2.62E-03 | 43759 |
| 11 | 2661919 | rs231356 | *KCNQ1* | T/A | 0.27 | 1.14(1.07-1.21) | 2.84E-05 | 9.11E-01 | 20431 | 1.05(0.98-1.14) | 1.68E-01 | 3.26E-01 | 9433 | 1.11(1.06-1.16) | 1.94E-05 | 1.08E-01 | 29864 | 1.08(1.04-1.13) | 4.37E-04 | 22570 | 1.09(1.06-1.13) | 3.93E-08 | 5.27E-01 | 52434 |
| 11 | 2796327 | rs2237892 | *KCNQ1* | C/T | 0.89 | 1.22(1.13-1.32) | 3.44E-07 | 1.76E-01 | 23679 | 1.2(1.07-1.34) | 1.48E-03 | 6.86E-01 | 7580 | 1.21(1.14-1.29) | 6.26E-10 | 7.85E-01 | 31259 | 1.14(1.05-1.24) | 2.70E-03 | 22570 | 1.19(1.13-1.25) | 1.20E-11 | 2.62E-01 | 53829 |
| 11 | 2806106 | rs2283228 | *KCNQ1* | A/C | 0.89 | 1.22(1.14-1.31) | 6.10E-08 | 9.48E-02 | 23819 | 1.17(1.06-1.28) | 1.04E-03 | 7.10E-01 | 11397 | 1.2(1.14-1.27) | 9.90E-11 | 4.34E-01 | 35216 | 1.16(1.06-1.26) | 9.73E-04 | 22570 | 1.19(1.13-1.24) | 4.87E-13 | 4.90E-01 | 57786 |
| 12 | 64451469 | rs12049974 | *HMGA2* | T/A | 0.42 | 1.13(1.07-1.19) | 4.31E-06 | 3.84E-01 | 20431 | 1.05(0.97-1.13) | 2.24E-01 | 2.54E-01 | 7638 | 1.11(1.06-1.15) | 2.78E-06 | 1.01E-01 | 28069 | 0.98(0.88-1.09) | 6.64E-01 | 22570 | 1.09(1.05-1.13) | 2.71E-05 | 3.65E-02 | 50639 |
| 12 | 64537207 | rs343092 | *HMGA2* | T/G | 0.81 | 1.16(1.09-1.24) | 1.91E-06 | 9.48E-01 | 23780 | 1.15(1.04-1.26) | 3.99E-03 | 3.37E-01 | 8820 | 1.16(1.1-1.22) | 8.79E-09 | 7.93E-01 | 32600 | 1.12(1.06-1.19) | 5.43E-05 | 22570 | 1.14(1.1-1.19) | 2.75E-12 | 4.41E-01 | 55170 |
| 15 | 58155498 | rs335810 | *ANXA2* | A/C | 0.69 | 1.12(1.07-1.18) | 6.05E-06 | 7.54E-01 | 21966 | 1.04(0.96-1.13) | 3.49E-01 | 9.68E-01 | 7652 | 1.1(1.06-1.15) | 6.77E-06 | 9.92E-02 | 29618 | | | | 1.1(1.06-1.15) | 6.77E-06 | 1.00E+00 | 29618 |
| 16 | 59250689 | rs1423882 | *GNPATP* | A/G | 0.34 | 1.12(1.07-1.18) | 8.21E-06 | 7.84E-01 | 21325 | 0.99(0.93-1.05) | 7.51E-01 | 9.45E-01 | 11400 | 1.07(1.03-1.11) | 6.16E-04 | 1.34E-03 | 32725 | 0.98(0.93-1.03) | 3.76E-01 | 22570 | 1.04(1-1.07) | 2.30E-02 | 9.40E-03 | 55295 |

Abbreviations: Chr, chromosome; RAF, risk allele frequency; OR, odds ratio; CI, confidence interval; $P_{het}$, heterogeneity *P* value; N $_{eff}$, effective sample size

[a]Alleles are ordered as risk allele/other allele aligned to the forward strand of NCBI Build 36

[b]Odds ratio are reported with respect to the risk allele

[c]For SNPs showing significant heterogeneous effect sizes in studies within a stage or between stages ($P_{het}$<0.001), association results were presented using random effect model. In addition, association results for the meta-analysis including studies from all stages were presented using random model effect.
