# Supplementary material for: Meta-Analysis of Genome-Wide Association Studies in African Americans Provides Insights into the Genetic Architecture of Type 2 Diabetes
Source: PLoS Genet. 2014 Aug 7;10(8):e1004517. doi: 10.1371/journal.pgen.1004517 (PMC4125087; doi:10.1371/journal.pgen.1004517)
Supplement: Table S5 — Stage 1 GWAS meta-analysis results for index SNPs at established T2D or glucose homeostasis loci in African Americans. (PDF) [file pgen.1004517.s009.pdf]

Table S5. Stage 1 GWAS meta-analysis results for index SNPs at established T2D or glucose homeostasis loci in African Americans.

					Literature				MEDIA											
										BMI unadjusted			BMI adjusted			Power	Power			
Locus	Reported locus	Chr	Position	SNP	Risk allele[a]	RAF	OR (95% CI)[b]	*P*	RAF	OR (95% CI)[c]	*P*	P_{het}	OR (95% CI)[d]	*P*	P_{het}	at P = 0.05[e]	at P = 5×10^{-8} [e]	GWAS locus	Reference (PMID)	Associated Trait(s)
1		1	54409755	rs625643	C	0.75		9.00E-03	0.64	0.97 (0.92-1.01)	1.61E-01		0.96 (0.92-1.01)	1.20E-01				Y	17848626	Fasting insulin
2	*PDE4B*	1	66464473	rs952635	T	0.69		4.00E-04	0.67	1 (0.94-1.05)	8.90E-01		0.99 (0.93-1.04)	6.19E-01				Y	17848626	Fasting glucose
3	*ABCD3, F3, SLC44A3*	1	94842629	rs7542900	C	0.54	1.16 (1.09-1.25)	6.00E-06	0.58	0.93 (0.89-0.98)	3.45E-03	1.27E-06	0.92 (0.88-0.97)	9.30E-04	4.16E-07	1.00	1.00	Y	22238593	Type 2 diabetes
4	*SLC44A3*	1	95110679	rs7531174	G	0.21		6.00E-03	0.28	0.99 (0.94-1.04)	5.80E-01		0.98 (0.93-1.04)	5.43E-01				Y	17848626	Fasting glucose
5	*NOTCH2*	1	120319482	rs10923931	T	0.11	1.13 (1.08-1.17)	4.10E-08	0.32	1.04 (1.00-1.10)	7.08E-02	8.98E-03	1.05 (1.00-1.11)	3.56E-02	2.45E-02	1.00	0.95	Y	18372903	Type 2 diabetes
6	*CR2*	1	205718799	rs17045328	G	0.3	1.38 (1.20-1.59)	6.92E-06	0.03	1 (0.85-1.17)	9.65E-01	2.49E-03	0.99 (0.84-1.16)	8.92E-01	2.44E-03	1.00	0.99	Y	21490949	Type 2 diabetes
7	*PROX1*	1	212225879	rs340874	C	0.52		6.60E-12	0.18	1.03 (0.97-1.10)	3.39E-01		1.04 (0.97-1.11)	3.12E-01				Y	20081858	Fasting glucose-related traits
8	*LYPLAL1*	1	217707303	rs2820436	C	0.67		4.40E-09	0.52	1 (0.95-1.04)	8.72E-01		1.01 (0.97-1.06)	6.32E-01				Y	22885924	Fasting insulin
9	*PCNXL2*	1	231406777	rs12027542	A	0.61	1.41 (1.23-1.61)	4.33E-07	0.96	1.01 (0.89-1.14)	9.01E-01	3.01E-04	0.99 (0.87-1.12)	8.27E-01	1.55E-04	1.00	0.68	Y	21490949	Type 2 diabetes
10	*MTR, RYR2*	1	235212541	rs4659485	T	0.9	1.3 (1.15-1.47)	1.90E-05	0.89	0.97 (0.90-1.05)	5.17E-01	1.09E-04	0.97 (0.89-1.05)	4.37E-01	9.24E-05	1.00	0.99	Y	22238593	Type 2 diabetes
11	*DCDC2C*	2	3819295	rs11677370	T	0.4	1.35 (1.19-1.53)	3.39E-06	0.69	1.03 (0.98-1.08)	2.78E-01	7.07E-05	1.04 (0.98-1.09)	1.79E-01	1.29E-04	1.00	1.00	Y	21490949	Type 2 diabetes
12	*GCKR*	2	27594741	rs780094	C	0.62		6.00E-38	0.82	1.05 (0.99-1.11)	1.38E-01		1.02 (0.96-1.08)	6.06E-01				Y	20081858	Fasting glucose-related traits, Fasting insulin-related traits
13	*THADA*	2	43586327	rs7578597	T	0.9	1.15 (1.10-1.20)	1.10E-09	0.74	1.05 (1.00-1.11)	6.03E-02	8.35E-03	1.06 (1.01-1.12)	2.96E-02	2.27E-02	1.00	0.93	Y	18372903	Type 2 diabetes
14	*BCL11A*	2	60422249	rs243088	T	0.45	1.07 (1.04-1.09)	1.80E-08	0.54	1.09 (1.04-1.15)	5.09E-04	4.49E-01	1.09 (1.03-1.15)	1.26E-03	5.40E-01	0.99	0.10	Y	22885922	Type 2 diabetes
15	*GPR45*	2	105204030	rs6712932	T	0.64	1.52 (1.27-1.82)	6.25E-06	0.72	1 (0.95-1.06)	8.93E-01	1.37E-05	1.01 (0.96-1.07)	6.65E-01	2.15E-05	1.00	1.00	Y	17668382	Type 2 diabetes
16	*YSK4*	2	135472099	rs1530559	A	0.52		3.40E-08	0.23	1 (0.95-1.06)	9.14E-01		1 (0.94-1.06)	9.71E-01				Y	22885924	Fasting insulin
17	*RND3*	2	151346182	rs7560163	C	0.87	1.33 (1.19-1.49)	7.00E-09	0.89	0.97 (0.90-1.05)	4.93E-01	1.03E-05	0.95 (0.87-1.05)	3.07E-01	8.57E-06	1.00	1.00	Y	22238593	Type 2 diabetes
18	*RBMS1*	2	160879700	rs7593730	C	0.77	1.11 (1.08-1.16)	3.70E-08	0.63	1.01 (0.96-1.06)	7.44E-01	3.18E-03	1.03 (0.98-1.08)	2.93E-01	1.84E-02	1.00	0.70	Y	20418489	Type 2 diabetes
19	*GRB14*	2	165237122	rs13389219	C	0.6	1.07 (1.05-1.10)	1.00E-08	0.29	1.06 (1.01-1.11)	3.05E-02	6.93E-01	1.09 (1.03-1.15)	1.88E-03	5.92E-01	0.97	0.06	Y	22885922	Type 2 diabetes
20	*G6PC2*	2	169471394	rs560887	C	0.7		9.00E-218	0.93	0.97 (0.88-1.07)	5.56E-01		0.95 (0.86-1.05)	3.40E-01				Y	20081858	Fasting glucose-related traits
21	*TMEFF2*	2	192622607	rs10497721				7.00E-07	0.07	1.01 (0.92-1.11)	8.62E-01		1.01 (0.91-1.11)	9.21E-01				Y	17903298	Diabetes (incident)
22	*IRS1*	2	226728897	rs7578326	A	0.64	1.11 (1.08-1.13)	5.40E-20	0.56	1.03 (0.97-1.08)	3.45E-01	5.43E-03	1.02 (0.97-1.08)	4.36E-01	4.72E-03	1.00	0.78	Y	20581827	Type 2 diabetes
23	*PPARG*	3	12368125	rs1801282	C	0.86	1.14 (1.08-1.20)	1.70E-06	0.98	1.08 (0.90-1.30)	4.23E-01	5.79E-01	1.09 (0.90-1.33)	3.69E-01	6.84E-01	0.51	0.00	Y	17463246	Type 2 diabetes, Fasting insulin-related traits

24	*AMT*	3	49430334	rs11715915	C	0.68		4.90E-08	0.76	0.98 (0.93-1.03)	4.15E-01		0.98 (0.93-1.03)	4.22E-01				Y	22885924	Fasting glucose
25	*NISCH*	3	52481466	rs6784615	T	0.94	1.19	1.50E-02	0.98	0.83 (0.70-0.98)	3.46E-02		0.81 (0.68-0.97)	2.27E-02		0.71	0.00	Y	20935629	Type 2 diabetes
26	*WNT5A*	3	55288440	rs358806	A	0.2	1.16 (1.03-1.33)	3.05E-06	0.10	1.03 (0.96-1.12)	4.11E-01	1.52E-01	1.01 (0.93-1.09)	8.62E-01	7.91E-02	1.00	0.69	Y	17554300	Type 2 diabetes
27	*PSMD6*	3	64023337	rs831571	C		1.09 (1.06-1.12)	8.41E-11	0.80	0.98 (0.93-1.04)	4.71E-01	6.61E-04	0.98 (0.92-1.04)	4.44E-01	8.26E-04	0.98	0.08	Y	22158537	Type 2 diabetes
28	*ADAMTS9*	3	64686944	rs4607103	C	0.76	1.09 (1.06-1.12)	1.20E-08	0.70	1.01 (0.96-1.06)	7.74E-01	4.23E-03	1.01 (0.96-1.06)	7.96E-01	6.81E-03	1.00	0.23	Y	18372903	Type 2 diabetes
29	*ZNF717*	3	76215254	rs1489100	T	0.59		7.00E-04	0.60	0.98 (0.93-1.02)	3.08E-01		0.97 (0.93-1.02)	2.69E-01				Y	17848626	A1c
30	*ZPLD1*	3	103685735	rs2063640	A	0.17	1.23 (1.13-1.34)	3.47E-06	0.06	0.97 (0.82-1.14)	6.94E-01	1.04E-02	0.98 (0.83-1.15)	7.68E-01	1.53E-02	1.00	0.89	Y	21490949	Type 2 diabetes
31	*MORC1*	3	110164908	rs2715755	A	0.61		3.00E-03	0.56	0.98 (0.94-1.02)	4.07E-01		0.98 (0.93-1.02)	3.02E-01				Y	17848626	Fasting insulin
32	*ADCY5*	3	124548468	rs11708067	A	0.78	1.12 (1.09-1.15)	7.00E-22	0.85	1.13 (1.06-1.21)	1.09E-04	7.35E-01	1.14 (1.07-1.22)	1.08E-04	6.29E-01	1.00	0.19	Y	20081858	Fasting glucose-related traits, Type 2 diabetes
33	*PLS1*	3	143913690	rs3773506	C	0.06	1.81 (1.39-2.35)	8.51E-06	0.16	1 (0.94-1.06)	9.09E-01	1.29E-05	0.99 (0.93-1.06)	8.08E-01	1.17E-05	1.00	1.00	Y	21490949	Type 2 diabetes
34	*SLC9A9*	3	144538397	rs729511	G	0.56		2.00E-03	0.26	0.98 (0.93-1.03)	3.94E-01		0.98 (0.93-1.03)	4.21E-01				Y	17848626	Fasting insulin
35	*SLC2A2*	3	172200215	rs11920090	T	0.87		8.00E-13	0.65	1 (0.96-1.05)	8.63E-01		1.01 (0.96-1.06)	7.89E-01				Y	20081858	Fasting glucose-related traits
36	*PEX5L*	3	181144012	rs7630877	A	0.17	1.32 (1.17-1.49)	7.25E-06	0.35	0.99 (0.94-1.04)	6.65E-01	1.57E-05	0.99 (0.94-1.04)	7.24E-01	1.94E-05	1.00	1.00	Y	21490949	Type 2 diabetes
37	*IGF2BP2*	3	187011774	rs1470579	C	0.3	1.14 (1.09-1.19)	2.17E-09	0.76	1.06 (1.00-1.14)	6.85E-02	8.17E-02	1.09 (1.01-1.16)	2.13E-02	2.35E-01	1.00	0.81	Y	20581827	Type 2 diabetes, Fasting glucose-related traits
38	*ST6GAL1*	3	188149155	rs16861329	C	0.75	1.08 (1.05-1.11)	1.30E-07	0.91	1 (0.90-1.11)	9.27E-01	1.39E-01	1.01 (0.90-1.12)	9.04E-01	2.20E-01	0.73	0.00	Y	21874001	Type 2 diabetes
39	*MAEA*	4	1299901	rs6815464	C		1.13 (1.10-1.16)	1.57E-20										Y	22158537	Type 2 diabetes
40	*WFS1*	4	6353923	rs1801214	T	0.59	1.13 (1.08-1.18)	3.16E-08	0.66	0.99 (0.94-1.05)	7.66E-01	1.83E-04	0.99 (0.94-1.05)	7.56E-01	2.34E-04	1.00	0.91	Y	20581827	Type 2 diabetes
41	*FAM13A*	4	89960292	rs3822072	A	0.48		1.80E-08	0.58	1.06 (1.00-1.11)	3.41E-02		1.07 (1.01-1.13)	1.17E-02				Y	22885924	Fasting insulin (BMI adjusted)
42	*TET2*	4	106301085	rs9884482	C	0.39		1.40E-11	0.12	1.02 (0.95-1.09)	6.62E-01		1.02 (0.94-1.10)	6.41E-01				Y	22885924	Fasting insulin
43	*PRDM5*	4	122021240	rs180730				5.00E-06	0.36	1.02 (0.97-1.07)	4.04E-01		1.01 (0.96-1.06)	7.10E-01				Y	17903298	Fasting glucose
44	*TMEM155*	4	122884964	rs7659604	T	0.4	1.35 (1.19-1.54)	9.42E-06	0.67	0.99 (0.94-1.04)	6.92E-01	1.48E-05	0.98 (0.93-1.03)	4.72E-01	9.42E-06	1.00	1.00	Y	17554300	Type 2 diabetes
45	*PDGFC*	4	157954125	rs6822892	A	0.68		2.60E-10	0.26	1.01 (0.96-1.07)	6.51E-01		1.02 (0.97-1.08)	4.53E-01				Y	22885924	Fasting insulin (BMI adjusted)
46	*MARCH1*	4	164752251	rs3792615	T	0.95	1.93 (1.45-2.59)	9.38E-06	0.87	0.99 (0.93-1.06)	7.74E-01	1.46E-05	0.99 (0.92-1.06)	7.18E-01	1.38E-05	1.00	1.00	Y	21490949	Type 2 diabetes
47	*ARL15*	5	53308421	rs4865796	A	0.67		2.20E-12	0.75	1.05 (1.00-1.11)	4.59E-02		1.06 (1.00-1.11)	4.94E-02				Y	22885924	Fasting insulin (BMI adjusted)
48	*ANKRD55, MAP3K1*	5	55842508	rs459193	G	0.7	1.08 (1.05-1.11)	6.00E-09	0.60	1.08 (1.02-1.13)	3.24E-03	8.78E-01	1.08 (1.03-1.14)	1.60E-03	9.06E-01	1.00	0.19	Y	22885922	Type 2 diabetes, Fasting insulin (BMI adjusted)
49	*ZBED3*	5	76460705	rs4457053	G	0.26	1.08 (1.06-1.11)	2.80E-12										Y	20581827	Type 2 diabetes
50	*CETN3*	5	89581865	rs12518099	G	0.227	1.16 (1.10-1.22)	6.89E-07	0.23	1.04 (0.98-1.10)	2.30E-01	4.95E-03	1.04 (0.97-1.10)	2.62E-01	5.33E-03	1.00	0.99	Y	19734900	Type 2 diabetes
51	*PCSK1*	5	95565204	rs4869272	T	0.69		1.00E-15	0.78	1.03 (0.98-	2.40E-01		1.04 (0.99-	1.43E-01				Y	22885924	Fasting glucose

										1.09)			1.11)							
52	*ERAP2*	5	96280573	rs1019503	A	0.48		8.90E-09	0.57	1.04 (0.99-1.09)	1.37E-01		1.04 (0.99-1.10)	8.42E-02				Y	22885924	Two-hour glucose challenge
53	*TENM2*	5	165446786	rs2545523	T	0.2		2.00E-03	0.51	1 (0.96-1.05)	9.48E-01		1 (0.96-1.05)	8.50E-01				Y	17848626	Fasting insulin
54	*RREB1*	6	7158199	rs17762454	T	0.26		9.60E-09	0.15	1 (0.93-1.07)	9.68E-01		1 (0.93-1.07)	9.45E-01				Y	22885924	Fasting glucose (BMI adjusted)
55	*CDKAL1*	6	20796100	rs10440833	A	0.29	1.25 (1.20-1.31)	1.84E-22	0.22	1.06 (1.00-1.12)	4.28E-02	5.85E-06	1.06 (1.00-1.13)	3.59E-02	1.68E-05	1.00	1.00	Y	20581827	Type 2 diabetes
56	*UHRF1BP1*	6	34872900	rs6912327	T	0.8		2.30E-08	0.34	0.96 (0.92-1.01)	1.23E-01		0.96 (0.91-1.01)	8.47E-02				Y	22885924	Fasting insulin (BMI adjusted)
57	*ZFAND3*	6	38214822	rs9470794	C		1.12 (1.08-1.16)	2.06E-10	0.14	0.99 (0.93-1.06)	7.89E-01	1.31E-03	1.01 (0.95-1.08)	7.10E-01	9.94E-03	1.00	0.37	Y	22158537	Type 2 diabetes
58	*KCNK16*	6	39292028	rs1535500	T		1.08 (1.05-1.11)	2.30E-08	0.83	1.01 (0.95-1.08)	7.84E-01	5.96E-02	1.01 (0.95-1.08)	7.29E-01	7.90E-02	0.93	0.02	Y	22158537	Type 2 diabetes
59	*VEGFA*	6	43919740	rs9472138	T	0.28	1.06 (1.04-1.09)	4.00E-06	0.19	0.98 (0.93-1.04)	6.00E-01	2.35E-02	0.98 (0.93-1.04)	5.48E-01	2.24E-02	0.83	0.01	Y	18372903	Type 2 diabetes
60	*C6orf57*	6	71345910	rs1048886	G	0.18	1.26 (1.14-1.39)	3.48E-08	0.29	1.01 (0.96-1.06)	7.42E-01	6.76E-05	1.02 (0.97-1.07)	5.50E-01	1.29E-04	1.00	1.00	Y	21490949	Type 2 diabetes
61	*RSPO3*	6	127494628	rs2745353	T	0.51		5.50E-09	0.60	0.96 (0.91-1.00)	5.11E-02		0.97 (0.92-1.01)	1.69E-01				Y	22885924	Fasting insulin
62	*ENPP1*	6	132192132	rs2021966	A	0.61	1.27 (1.13-1.43)	2.60E-04	0.68	1 (0.96-1.05)	8.80E-01	3.17E-04	1 (0.95-1.05)	9.69E-01	2.91E-04	1.00	1.00	N	18678618	Type 2 diabetes
63	*LOC100132735*	6	140315340	rs642858	A	0.4	1.35 (1.19-1.53)	2.15E-06	0.15	0.98 (0.92-1.05)	6.13E-01	9.62E-06	0.99 (0.93-1.06)	7.70E-01	1.67E-05	1.00	1.00	Y	21490949	Type 2 diabetes
64	*DGKB*	7	14864807	rs17168486	T	0.19	1.11 (1.07-1.14)	5.90E-11	0.11	1.06 (0.98-1.15)	1.44E-01	3.27E-01	1.05 (0.97-1.15)	2.33E-01	2.47E-01	0.99	0.12	Y	22885922	Type 2 diabetes
65	*DGKB*	7	15030834	rs2191349	T	0.52		3.00E-44	0.60	1.06 (1.01-1.11)	2.78E-02		1.06 (1.00-1.11)	3.40E-02				Y	20081858	Fasting glucose-related traits
66	*JAZF1*	7	28162747	rs849134	A	0.51	1.13 (1.09-1.18)	2.80E-09	0.74	1.06 (1.01-1.12)	2.56E-02	6.30E-02	1.07 (1.01-1.13)	1.93E-02	9.93E-02	1.00	0.77	Y	20581827	Type 2 diabetes
67	*CPVL*	7	29151014	rs10486607				8.00E-06	0.08	1.01 (0.92-1.11)	8.01E-01		1.01 (0.92-1.11)	8.16E-01				Y	17903298	Diabetes related insulin traits
68	*GCK*	7	44202193	rs4607517	A	0.16		7.00E-92	0.11	1.02 (0.93-1.11)	7.28E-01		1.03 (0.94-1.12)	5.87E-01				Y	20081858	Fasting glucose-related traits
69	*ZPBP*	7	50063624	rs1355037	C	0.23		2.00E-02	0.21	1 (0.95-1.06)	9.26E-01		1.01 (0.95-1.06)	8.38E-01				Y	17848626	Fasting insulin
70	*GRB10*	7	50759073	rs6943153	T	0.34		1.60E-12	0.68	1.01 (0.96-1.06)	6.32E-01		1.01 (0.96-1.06)	8.22E-01				Y	22885924	Fasting glucose
71	*HIP1*	7	75014132	rs1167800	A	0.54		2.60E-09	0.85	1.04 (0.96-1.13)	3.12E-01		1.04 (0.96-1.13)	3.06E-01				Y	22885924	Fasting insulin
72	*ACHE*	7	100328013	rs7636	A	0.06	1.85 (1.42-2.41)	4.99E-06	0.25	0.97 (0.91-1.03)	2.60E-01	2.66E-06	0.97 (0.91-1.03)	2.67E-01	2.69E-06	1.00	1.00	Y	21490949	Type 2 diabetes
73	*GCC1, PAX4*	7	126952194	rs6467136	G		1.11 (1.07-1.14)	4.96E-11	0.65	1.01 (0.96-1.06)	8.04E-01	7.84E-04	1.01 (0.96-1.06)	7.28E-01	9.92E-04	1.00	0.67	Y	22158537	Type 2 diabetes
74	*NRF1*	7	129184880	rs1882095	T	0.38	1.24 (1.10-1.40)	9.60E-04	0.50	1.05 (1.00-1.11)	3.57E-02	1.43E-02	1.05 (1.00-1.10)	6.28E-02	1.24E-02	1.00	1.00	N	18678618	Type 2 diabetes
75	*KLF14*	7	130117394	rs972283	G	0.55	1.07 (1.05-1.10)	2.20E-10	0.84	1.02 (0.95-1.10)	6.05E-01	2.17E-01	1.02 (0.95-1.10)	5.55E-01	2.66E-01	0.84	0.01	Y	20581827	Type 2 diabetes
76	*PPP1R3B*	8	9215142	rs983309	T	0.12		6.30E-15	0.28	1.02 (0.96-1.08)	5.16E-01		1.03 (0.97-1.09)	2.89E-01				Y	22885924	Fasting glucose, Fasting insulin
77	*ZMAT4*	8	40603396	rs2722425				9.00E-06	0.37	1.03 (0.98-1.08)	2.14E-01		1.02 (0.97-1.07)	4.03E-01				Y	17903298	Fasting glucose
78	*ANK1*	8	41638405	rs516946	C	0.76	1.09 (1.06-1.12)	2.50E-10	0.77	0.95 (0.90-1.01)	7.65E-02	9.38E-06	0.96 (0.90-1.01)	1.06E-01	2.57E-05	0.99	0.12	Y	22885922	Type 2 diabetes
79	*TP53INP1*	8	96029687	rs896854	T	0.48	1.06 (1.04-1.09)	9.90E-10	0.69	1.02 (0.97-1.07)	3.63E-01	2.14E-01	1.01 (0.96-1.06)	6.38E-01	1.19E-01	0.91	0.01	Y	20581827	Type 2 diabetes
80	*SLC30A8*	8	118254206	rs3802177	G	0.5	1.15 (1.10-	1.23E-06	0.90	1.15 (1.05-	1.50E-03	9.77E-01	1.17 (1.07-	5.40E-04	7.26E-01	1.00	0.19	Y	20581827	Type 2 diabetes

							1.21)			1.25)			1.28)							
81	*GLIS3*	9	4277466	rs7041847	A		1.1 (1.07-1.13)	1.99E-14	0.87	1.08 (1.00-1.15)	3.97E-02	5.60E-01	1.08 (1.00-1.16)	4.55E-02	5.98E-01	0.96	0.04	Y	22158537	Type 2 diabetes
82	*PTPRD*	9	8869118	rs17584499	T	0.07	1.57 (1.36-1.82)	3.07E-09										Y	20174558	Type 2 diabetes
83	*PTPRD*	9	10420602	rs649891	C	0.41	1.4	5.80E-06	0.63	0.99 (0.93-1.05)	6.78E-01		1 (0.94-1.06)	9.30E-01		1.00	1.00	Y	21647700	Type 2 diabetes
84	*CDKN2B-AS*	9	22019547	rs564398	T		1.12 (1.07-1.17)	1.20E-07	0.92	1 (0.92-1.10)	9.18E-01	3.17E-02	1.03 (0.94-1.13)	4.83E-01	1.26E-01	0.93	0.02	Y	17463249	Type 2 diabetes
85	*CDKN2B-AS*	9	22123284	rs10965250	G	0.83	1.2 (1.13-1.27)	1.23E-10										Y	20581827	Type 2 diabetes
86	*TLE4*	9	81141948	rs13292136	C	0.93	1.11 (1.07-1.15)	2.80E-08	0.91	1.07 (0.99-1.16)	9.73E-02	3.92E-01	1.08 (0.99-1.17)	8.24E-02	4.89E-01	0.92	0.02	Y	20581827	Type 2 diabetes
87	*TLE1*	9	83498768	rs2796441	G	0.57	1.07 (1.05-1.10)	5.40E-09	0.83	1.01 (0.95-1.07)	7.42E-01	8.23E-02	1.02 (0.96-1.08)	5.47E-01	1.59E-01	0.86	0.01	Y	22885922	Type 2 diabetes
88	*IKBKAP*	9	110720180	rs16913693	T	0.97		3.50E-11										Y	22885924	Fasting glucose
89	*SLC31A2*	9	114933365	rs4457406	T	0.35	1.35 (1.18-1.54)	9.95E-06	0.25	0.99 (0.94-1.04)	6.71E-01	1.58E-05	0.99 (0.94-1.05)	8.53E-01	2.59E-05	1.00	1.00	Y	21490949	Type 2 diabetes
90	*RAPGEF1*	9	133438117	rs4740283	G	0.1	3.12 (1.73-5.63)	1.30E-04	0.07	0.96 (0.87-1.05)	3.43E-01	1.05E-04	0.94 (0.86-1.04)	2.19E-01	8.65E-05	1.00	1.00	N	18678618	Type 2 diabetes
91	*DNLZ*	9	138376587	rs3829109	G	0.71		1.10E-10	0.81	1.07 (0.99-1.16)	7.64E-02		1.08 (1.00-1.17)	6.56E-02				Y	22885924	Fasting glucose
92	*CDC123*	10	12368016	rs12779790	G	0.18	1.11 (1.07-1.14)	1.20E-10	0.13	1.09 (1.01-1.17)	2.69E-02	6.11E-01	1.08 (1.00-1.17)	4.58E-02	5.41E-01	1.00	0.19	Y	18372903	Type 2 diabetes
93	*VPS26A*	10	70601480	rs1802295	T	0.26	1.07 (1.05-1.10)	2.10E-08	0.06	1 (0.91-1.11)	9.30E-01	2.23E-01	1.01 (0.91-1.12)	9.09E-01	2.58E-01	0.55	0.00	Y	21874001	Type 2 diabetes
94	*ZMIZ1*	10	80612637	rs12571751	A	0.52	1.08 (1.05-1.10)	1.00E-10	0.54	1.03 (0.98-1.07)	2.19E-01	4.45E-02	1.02 (0.98-1.07)	3.48E-01	3.01E-02	1.00	0.23	Y	22885922	Type 2 diabetes
95	*HHEX*	10	94452862	rs1111875	C	0.53	1.13 (1.08-1.17)	5.70E-10	0.77	1.07 (1.02-1.13)	8.46E-03	1.08E-01	1.08 (1.02-1.14)	5.54E-03	1.80E-01	1.00	0.66	Y	17463246	Type 2 diabetes
96	*SORCS1*	10	109034861	rs1416406	A	0.27		3.00E-03	0.49	1.01 (0.96-1.05)	7.96E-01		1.02 (0.97-1.06)	5.29E-01				Y	17848626	Fasting insulin
97	*ADRA2A*	10	113032083	rs10885122	G	0.87		3.00E-16	0.33	1.03 (0.98-1.08)	3.07E-01		1.03 (0.97-1.08)	3.38E-01				Y	20081858	Fasting glucose-related traits
98	*TCF7L2*	10	114748339	rs7903146	T	0.27	1.4 (1.34-1.46)	2.21E-51	0.30	1.32 (1.25-1.40)	6.62E-24	1.12E-01	1.36 (1.28-1.44)	3.01E-26	3.97E-01	1.00	1.00	Y	20581827	Type 2 diabetes, Fasting glucose-related traits, Fasting insulin-related traits
99	*TCERG1L*	10	132837952	rs10741243	G	0.93	1.75 (1.38-2.23)	5.33E-06	0.60	0.98 (0.94-1.03)	4.88E-01	4.76E-06	0.99 (0.94-1.04)	6.77E-01	6.25E-06	1.00	1.00	Y	21490949	Type 2 diabetes
100	*TH, INS*	11	2150416	rs10770141	A	0.39	1.07 (1.04-1.10)	1.57E-06										N	22325160	Type 2 diabetes
101	*KCNQ1*	11	2648047	rs231362	G	0.52	1.08 (1.06-1.10)	2.80E-13	0.78	1.07 (1.00-1.14)	3.96E-02	8.07E-01	1.09 (1.02-1.17)	1.03E-02	7.30E-01	0.97	0.05	Y	20581827	Type 2 diabetes
102	*KCNQ1*	11	2803645	rs163184	G	0.5	1.09 (1.06-1.11)	1.20E-11	0.20	1.04 (0.98-1.11)	1.67E-01	2.03E-01	1.05 (0.98-1.12)	1.72E-01	2.39E-01	0.99	0.15	Y	22885922	Type 2 diabetes
103	*NLRP14*	11	7112939	rs10500679	G	0.28		2.00E-03	0.20	0.96 (0.90-1.01)	1.35E-01		0.95 (0.89-1.01)	8.80E-02				Y	17848626	Fasting insulin
104	*GALNTL4*	11	11184950	rs2722769	C	0.9	1.35 (1.19-1.54)	1.70E-06	0.91	0.99 (0.91-1.08)	8.37E-01	1.00E-04	1.01 (0.92-1.10)	9.00E-01	2.63E-04	1.00	1.00	Y	22238593	Type 2 diabetes
105	*KCNJ11*	11	17365206	rs5215	C		1.16 (1.09-1.23)	4.10E-07	0.09	1.08 (1.00-1.17)	3.75E-02	1.69E-01	1.11 (1.03-1.21)	8.25E-03	4.29E-01	1.00	0.57	Y	18372903	Type 2 diabetes
106	*LOC100507205*	11	41871942	rs9300039	C	0.89	1.25 (1.15-1.37)	4.30E-07	0.89	1.02 (0.95-1.10)	5.28E-01	6.09E-04	1.03 (0.96-1.11)	4.44E-01	1.11E-03	1.00	0.94	Y	17463248	Type 2 diabetes
107	*CRY2*	11	45829667	rs11605924	A	0.49		1.00E-14	0.86	1.01 (0.95-1.08)	7.00E-01		1 (0.94-1.08)	9.00E-01				Y	20081858	Fasting glucose-related traits
108	*MADD*	11	47292896	rs7944584	A	0.75		2.00E-18	0.95	0.9 (0.81-	7.57E-02		0.89 (0.79-	5.79E-02				Y	20081858	Fasting glucose-

										1.01)			1.00)							related traits
109	*FADS1*	11	61328054	rs174550	T	0.64		2.00E-15	0.91	1.05 (0.96-1.14)	2.62E-01		1.03 (0.94-1.13)	5.17E-01				Y	20081858	Fasting glucose-related traits
110	*ARAP1*	11	72110746	rs1552224	A	0.88	1.14 (1.11-1.17)	1.40E-22	0.97	0.9 (0.72-1.11)	3.22E-01	3.11E-02				0.68	0.00	Y	20581827	Type 2 diabetes
111	*MTNR1B*	11	92313476	rs1387153	T	0.29	1.09 (1.06-1.11)	2.00E-36	0.37	1.04 (0.99-1.09)	1.45E-01	9.02E-02	1.05 (1.00-1.11)	6.92E-02	2.21E-01	1.00	0.40	Y	20581827	Type 2 diabetes, Fasting glucose
112	*BARX2*	11	128978900	rs7107217	C	0.52	1.18 (1.10-1.27)	3.20E-07	0.55	1.05 (1.00-1.10)	4.48E-02	7.51E-03	1.04 (0.99-1.09)	9.64E-02	5.56E-03	1.00	1.00	Y	22238593	Type 2 diabetes
113	*CCND2*	12	4244634	rs11063069	G	0.21	1.08 (1.05-1.11)	3.30E-07	0.19	1.02 (0.97-1.08)	4.34E-01	9.74E-02	1.04 (0.98-1.10)	2.23E-01	2.45E-01	0.97	0.06	Y	22885922	Type 2 diabetes
114	*ITPR2, SSPN*	12	26344550	rs718314			1.08	4.10E-02	0.81	0.98 (0.93-1.04)	5.55E-01		0.99 (0.93-1.05)	6.84E-01		0.95	0.03	Y	20935629	Type 2 diabetes
115	*KLHDC5*	12	27856417	rs10842994	C	0.8	1.1 (1.06-1.13)	6.10E-10	0.95	0.99 (0.88-1.11)	8.91E-01	9.06E-02	0.99 (0.87-1.12)	8.51E-01	9.40E-02	0.66	0.00	Y	22885922	Type 2 diabetes
116	*HIGD1C*	12	49643809	rs12304921	G	0.16	2.5 (1.53-4.09)	7.07E-06	0.18	1 (0.94-1.06)	9.66E-01	2.86E-04	1.01 (0.95-1.07)	7.77E-01	3.37E-04	1.00	1.00	Y	17554300	Type 2 diabetes
117	*DCD*	12	53385263	rs1153188	A	0.73	1.08 (1.05-1.11)	1.80E-07	0.76	1 (0.95-1.05)	8.69E-01	6.78E-03	1 (0.95-1.06)	9.20E-01	1.72E-02	0.97	0.06	Y	18372903	Type 2 diabetes
118	*GLS2*	12	55151605	rs2657879	G	0.18		3.90E-08	0.07	0.95 (0.87-1.04)	2.86E-01		0.94 (0.85-1.03)	1.88E-01				Y	22885924	Fasting glucose (BMI adjusted)
119	*HMGA2*	12	64461161	rs1531343	C	0.1	1.1 (1.07-1.14)	3.60E-09	0.38	0.98 (0.93-1.02)	3.52E-01	8.85E-05	0.99 (0.94-1.04)	7.08E-01	6.98E-04	1.00	0.62	Y	20581827	Type 2 diabetes
120	*TSPAN8*	12	69863368	rs1495377	G	0.55	1.28 (1.11-1.49)	6.52E-06	0.25	1.02 (0.97-1.08)	4.00E-01	6.07E-03	1.03 (0.98-1.09)	2.86E-01	8.25E-03	1.00	1.00	Y	17554300	Type 2 diabetes
121	*TSPAN8*	12	69921061	rs4760790	A	0.26	1.11 (1.06-1.16)	3.56E-06	0.17	0.96 (0.90-1.02)	1.77E-01	1.79E-04	0.98 (0.92-1.05)	5.82E-01	1.95E-03	1.00	0.33	Y	20581827	Type 2 diabetes
122	*IGF1*	12	101399699	rs35767	G	0.85		3.30E-08	0.58	1 (0.95-1.05)	9.75E-01		1.01 (0.96-1.07)	6.55E-01				Y	20081858	Fasting glucose-related traits, Fasting insulin-related traits
123	*HNF1A*	12	119945069	rs7957197	T	0.85	1.07 (1.05-1.10)	2.40E-08	0.86	0.98 (0.90-1.05)	5.36E-01	2.66E-02	0.99 (0.91-1.07)	7.85E-01	6.54E-02	0.80	0.00	Y	20581827	Type 2 diabetes
124	*P2RX2*	12	131551691	rs10747083	A	0.66		7.60E-09										Y	22885924	Fasting glucose
125	*PDX1*	13	27385599	rs11619319	G	0.23		1.30E-15	0.17	1.04 (0.97-1.10)	2.65E-01		1.02 (0.96-1.09)	4.72E-01				Y	22885924	Fasting glucose
126	*KL*	13	32452302	rs576674	G	0.15		2.30E-08	0.59	1.04 (0.99-1.09)	1.22E-01		1.05 (1.00-1.11)	4.59E-02				Y	22885924	Fasting glucose
127	*OLFM4*	13	53199780	rs2806739	T	0.22		6.00E-03	0.71	1.04 (0.99-1.10)	9.42E-02		1.05 (0.99-1.11)	8.05E-02				Y	17848626	Fasting glucose
128	*MIR548H4*	13	68428665	rs2066219				9.00E-06	0.65	1 (0.96-1.05)	9.50E-01		1 (0.95-1.05)	9.20E-01				Y	17903298	Diabetes related insulin traits
129	*SPRY2*	13	79615157	rs1359790	G	0.71	1.15 (1.10-1.20)	6.49E-09	0.89	1.05 (0.98-1.13)	1.86E-01	3.55E-02	1.05 (0.98-1.14)	1.76E-01	5.07E-02	1.00	0.25	Y	20862305	Type 2 diabetes
130	*LOC100505967*	14	26870017	rs2877832				2.00E-06	0.04	1 (0.88-1.14)	9.63E-01		0.99 (0.86-1.13)	8.48E-01				Y	17903298	Diabetes related insulin traits
131	*TSHR*	14	80628718	rs2241119	A	0.88		5.00E-04	0.47	1 (0.95-1.04)	8.86E-01		1 (0.96-1.05)	9.10E-01				Y	17848626	Fasting glucose
132	*WARS*	14	99909014	rs3783347	G	0.79		1.30E-10										Y	22885924	Fasting glucose
133	*DLK1*	14	100212643	rs730570	G		1.21 (1.12-1.31)	4.00E-06	0.66	1.05 (1.00-1.10)	6.94E-02	2.89E-03	1.05 (1.00-1.11)	5.57E-02	4.53E-03	1.00	1.00	Y	21573907	Type 2 diabetes
134	*ATP8B4*	15	47980845	rs2009833	A	0.35		5.00E-03	0.33	0.96 (0.91-1.01)	1.17E-01		0.97 (0.92-1.02)	2.83E-01				Y	17848626	Fasting glucose
135	*C2CD4A, C2CD4B*	15	60183681	rs7172432	A	0.56	1.14 (1.09-1.20)	7.48E-08	0.32	1.01 (0.96-1.06)	6.65E-01	7.81E-04	1.01 (0.96-1.06)	6.77E-01	9.81E-04	1.00	0.99	Y	20818381	Type 2 diabetes
136	*C2CD4A, C2CD4B*	15	60201306	rs1436953	C	0.64	1.14	7.75E-06	0.29	1 (0.95-1.05)	9.33E-01		1 (0.95-1.05)	9.67E-01		1.00	0.98	Y	21799836	Type 2 diabetes
137	*HMG20A*	15	75619817	rs7177055	A	0.68	1.08 (1.05-	4.60E-09	0.37	1 (0.95-	9.15E-01	1.62E-03	1 (0.96-1.05)	9.41E-01	3.90E-03	1.00	0.21	Y	22885922	Type 2 diabetes

							1.10)			1.04)										
138	*ZFAND6*	15	78219277	rs11634397	G	0.6	1.06 (1.04-1.08)	2.40E-09	0.44	0.98 (0.93-1.03)	3.74E-01	1.84E-03	0.98 (0.94-1.03)	5.29E-01	5.43E-03	0.95	0.03	Y	20581827	Type 2 diabetes
139	*AP3S2*	15	88175261	rs2028299	C	0.31	1.08 (1.06-1.11)	1.20E-11	0.31	1.1 (1.04-1.15)	4.58E-04	6.17E-01	1.11 (1.06-1.17)	7.51E-05	3.18E-01	0.99	0.17	Y	21874001	Type 2 diabetes
140	*PRC1*	15	89322341	rs8042680	A	0.22	1.07 (1.05-1.09)	2.40E-10	0.85	1 (0.91-1.09)	9.77E-01	1.35E-01	1 (0.91-1.10)	9.40E-01	1.90E-01	0.82	0.01	Y	20581827	Type 2 diabetes
141	*FTO*	16	52402988	rs11642841	A	0.42	1.13 (1.08-1.18)	3.40E-08	0.13	1.08 (1.00-1.17)	5.64E-02	3.48E-01	1.05 (0.97-1.14)	2.26E-01	1.40E-01	1.00	0.43	Y	20581827	Type 2 diabetes
142	*BCAR1*	16	73804746	rs7202877	T	0.89	1.12 (1.07-1.16)	3.50E-08	0.83	1 (0.94-1.07)	9.06E-01	4.95E-03	1 (0.93-1.07)	9.56E-01	4.04E-03	1.00	0.26	Y	22885922	Type 2 diabetes
143	*SRR*	17	2163008	rs391300	C	0.63	1.28 (1.18-1.39)	5.52E-08	0.49	0.99 (0.94-1.04)	7.38E-01	2.98E-07	0.99 (0.94-1.05)	7.39E-01	3.88E-07	1.00	1.00	Y	20174558	Type 2 diabetes
144	*TP53*	17	7520197	rs1042522	G	0.26	1.18 (1.08-1.30)	8.60E-04	0.56	1.03 (0.97-1.09)	2.96E-01	1.94E-02	1.04 (0.98-1.10)	1.94E-01	3.05E-02	1.00	1.00	N	18678618	Type 2 diabetes
145	*SREBF1*	17	17662182	rs4925115	A	0.38	1.07 (1.04-1.10)	2.62E-07	0.32	1 (0.94-1.06)	9.27E-01	5.36E-02	1.01 (0.95-1.07)	8.08E-01	8.32E-02	0.98	0.07	N	22325160	Type 2 diabetes
146	*HNF1B*	17	33172153	rs4430796	G	0.58	1.14 (1.08-1.20)	1.52E-06										Y	20581827	Type 2 diabetes
147	*LPIN2*	18	2938029	rs10460009	C	0.6	1.35 (1.18-1.54)	8.69E-06	0.94	1.02 (0.93-1.11)	7.47E-01	5.21E-04	1 (0.91-1.10)	9.53E-01	2.69E-04	1.00	0.86	Y	21490949	Type 2 diabetes
148	*MC4R*	18	56035730	rs12970134	A	0.27	1.08 (1.05-1.11)	1.20E-08	0.14	1.04 (0.97-1.10)	2.86E-01	2.29E-01	1.02 (0.96-1.09)	4.73E-01	1.48E-01	0.93	0.03	Y	22885922	Type 2 diabetes
149	*BCL2*	18	58996864	rs12454712	T	0.63	1.08 (1.04-1.11)	2.29E-06	0.75	1.03 (0.96-1.11)	3.97E-01	2.55E-01	1.03 (0.95-1.11)	4.48E-01	2.48E-01	0.98	0.07	N	22325160	Type 2 diabetes
150	*CILP2*	19	19268718	rs10401969	C	0.08	1.13 (1.09-1.18)	7.00E-09	0.17	1.04 (0.98-1.10)	2.55E-01	1.97E-02	1.03 (0.97-1.10)	2.99E-01	2.09E-02	1.00	0.68	Y	22885922	Type 2 diabetes
151	*PEPD*	19	38584848	rs3786897	A		1.1 (1.07-1.14)	1.30E-08	0.44	1.02 (0.97-1.08)	3.61E-01	2.01E-02	1.03 (0.98-1.08)	2.47E-01	4.08E-02	1.00	0.65	Y	22158537	Type 2 diabetes
152	*PAPL*	19	44272577	rs472265	G	0.22	1.39 (1.20-1.61)	9.19E-06	0.22	1 (0.94-1.07)	9.67E-01	5.49E-05	0.99 (0.93-1.05)	7.40E-01	3.22E-05	1.00	1.00	Y	21490949	Type 2 diabetes
153	*GIPR*	19	50850353	rs8108269	G	0.31	1.07 (1.04-1.10)	4.40E-07	0.42	0.97 (0.93-1.02)	2.65E-01	6.16E-04	0.99 (0.94-1.04)	6.43E-01	5.31E-03	0.99	0.10	Y	22885922	Type 2 diabetes
154	*GIPR*	19	50888474	rs2302593	C	0.5		9.30E-10	0.71	0.94 (0.87-1.03)	1.72E-01		0.95 (0.87-1.03)	2.31E-01				Y	22885924	Fasting glucose
155	*FOXA2*	20	22505099	rs6113722	G	0.96		2.50E-11	0.84	1.03 (0.97-1.09)	3.68E-01		1.04 (0.97-1.11)	2.83E-01				Y	22885924	Fasting glucose
156	*TOP1*	20	39177319	rs6072275	A	0.16		1.70E-08	0.08	1.04 (0.96-1.13)	3.33E-01		1.04 (0.96-1.14)	3.41E-01				Y	22885924	Fasting glucose
157	*HNF4A*	20	42422681	rs4812829	A	0.29	1.09 (1.06-1.12)	8.20E-12	0.11	1.12 (1.04-1.21)	1.89E-03	4.45E-01	1.15 (1.06-1.24)	4.86E-04	2.23E-01	0.94	0.02	Y	21874001	Type 2 diabetes
158	*HUNK*	21	32307057	rs2833610	A	0.57	1.17 (1.09-1.24)	3.90E-06	0.37	1 (0.95-1.05)	8.94E-01	4.98E-05	1.01 (0.96-1.06)	7.18E-01	2.48E-04	1.00	1.00	Y	21490949	Type 2 diabetes

Abbreviations: BMI, body mass index; Chr, chromosome; RAF, risk allele frequency; OR, odds ratio; CI, confidence interval; P_{het}, heterogeneity *P* value

[a] Risk allele refers to the forward strand of NCBI Build 36

[b] Odds ratio and *P* value are reported with respect to the risk allele from the literature

[c] Associations were performed with adjustment for age, sex, study sites, and study-specific principal components

[d] Associations were performed with adjustment for age, sex, study sites, study-specific principal components and body mass index

[e] Power is calculated for T2D associated SNPs at the reported effect sizes from the literature and allele frequency of risk alleles from this study at type 1 error rate of 0.05 and 5×10^{-8}, respectively.
