# Supplementary material for: Meta-Analysis of Genome-Wide Association Studies in African Americans Provides Insights into the Genetic Architecture of Type 2 Diabetes
Source: PLoS Genet. 2014 Aug 7;10(8):e1004517. doi: 10.1371/journal.pgen.1004517 (PMC4125087; doi:10.1371/journal.pgen.1004517)
Supplement: Table S6 — Locus-wide association at established T2D or glucose homeostasis loci in stage 1 GWAS meta-analysis in African Americans. (PDF) [file pgen.1004517.s010.pdf]

Table S6. Locus-wide association at established T2D or glucose homeostasis loci in stage 1 GWAS meta-analysis in African Americans.

Locus	Index SNP[a]	Chr	Position	No. of SNPs[b]	df	Regional SNP[c]	Position	Gene	P^d	P_{locus}^e	r^2 in ASW[f]	r^2 in CEU[f]
1	rs625643	1	54409755	49	4.42	rs10493175	54409565	*CYB5RL*	6.72E-03	2.97E-02	0.49	0.81
2	rs952635	1	66464473	117	3.64	rs10493400	66468812	*PDE4B*	6.46E-03	2.35E-02	0.02	
2				117	3.64	rs543589	66509648	*PDE4B*	1.22E-02	4.44E-02	0.05	0.42
2				117	3.64	rs502958	66503770	*PDE4B*	1.24E-02	4.50E-02	0.06	0.56
3	rs7542900	1	94842629	63	6.52	rs1098726	94837608	*ABCD3, F3, SLC44A3*	1.29E-03	8.41E-03	0.33	0.96
3				63	6.52	rs7542900	94842629	*ABCD3, F3, SLC44A3*	3.50E-03	2.28E-02	1.00	1.00
3				63	6.52	rs841708	94838221	*ABCD3, F3*	6.43E-03	4.19E-02	0.10	0.23
4	rs7531174	1	95110679	21	2.68	rs17112592	95109720	*SLC44A3*	6.01E-02	1.61E-01	0.08	0.08
5	rs10923931	1	120319482	168	5.45	rs12075171	120161765	*REG4*	8.21E-03	4.48E-02	0.00	
6	rs17045328	1	205718799	242	4.99	rs11584921	205508598	*CD55*	1.13E-02	5.64E-02	0.01	0.01
7	rs340874	1	212225879	44	4.96	rs7548778	212200091	*PROX1*	2.58E-02	1.28E-01	0.06	0.15
8	rs2820436	1	217707303	281	7.38	rs7541594	217856058	*SLC30A10*	9.01E-03	6.65E-02	0.01	0.14
9	rs12027542	1	231406777	63	4.98	rs12034881	231375185	*PCNXL2*	2.85E-03	1.42E-02	0.42	0.92
10	rs4659485	1	235212541	46	4.58	rs1252171	235211136	*MTR, RYR2*	4.48E-02	2.05E-01	0.00	0.16
11	rs11677370	2	3819295	116	5.21	rs13431608	3814133	*DCDC2C*	9.62E-03	5.01E-02	0.00	
12	rs780094	2	27594741	283	5.92	rs1260341	27516719	*NRBP1*	1.68E-03	9.93E-03	0.08	0.43
12				283	5.92	rs1260333	27602128	*GCKR*	2.18E-03	1.29E-02	0.24	0.81
12				283	5.92	rs2911711	27604050	*GCKR*	2.19E-03	1.30E-02	0.23	0.73
12				283	5.92	rs780117	27551847	*IFT172*	3.44E-03	2.04E-02	0.08	0.43
12				283	5.92	rs1260345	27556999	*IFT172*	4.06E-03	2.40E-02	0.08	0.44
12				283	5.92	rs780100	27505657	*NRBP1*	4.23E-03	2.50E-02	0.11	0.43
12				283	5.92	rs1647276	27542105	*IFT172*	4.58E-03	2.71E-02	0.08	0.43
12				283	5.92	rs4803	27520801	*IFT172, KRTCAP3*	4.70E-03	2.78E-02	0.08	0.43
12				283	5.92	rs780107	27538238	*IFT172*	4.73E-03	2.80E-02	0.08	0.43
12				283	5.92	rs780106	27535102	*IFT172*	4.76E-03	2.81E-02	0.08	0.43
12				283	5.92	rs1260342	27516920	*NRBP1*	5.01E-03	2.96E-02	0.08	0.43
12				283	5.92	rs10208529	27639692	*C2orf16, ZNF512*	6.74E-03	3.99E-02	0.02	0.43
12				283	5.92	rs4425043	27586956	*GCKR*	7.08E-03	4.19E-02	0.01	0.43
12				283	5.92	rs1647266	27546989	*IFT172*	8.02E-03	4.75E-02	0.08	0.43
12				283	5.92	rs813592	27575475	*GCKR*	8.09E-03	4.79E-02	0.07	0.45
12				283	5.92	rs780104	27531195	*IFT172*	8.32E-03	4.92E-02	0.08	0.43
13	rs7578597	2	43586327	525	6.56	rs6544643	43413576	*THADA*	2.81E-04	1.84E-03	0.36	0.46
13				525	6.56	rs6760797	43413116	*THADA*	3.23E-04	2.12E-03	0.39	0.46
13				525	6.56	rs10166994	43411481	*THADA*	4.38E-04	2.87E-03	0.39	0.46
13				525	6.56	rs13431070	43575458	*THADA*	4.51E-04	2.96E-03	0.45	1.00
13				525	6.56	rs10167415	43405964	*THADA*	4.80E-04	3.15E-03	0.30	0.60
13				525	6.56	rs6544639	43363601	*THADA*	5.41E-04	3.55E-03	0.38	0.50
13				525	6.56	rs13390381	43387918	*THADA*	5.54E-04	3.64E-03	0.37	0.39
13				525	6.56	rs4608575	43510428	*THADA*	5.91E-04	3.88E-03	0.26	0.11
13				525	6.56	rs7603255	43347681	*THADA*	7.41E-04	4.86E-03	0.38	0.53
13				525	6.56	rs1322	43374671	*THADA*	8.10E-04	5.32E-03	0.43	0.48
13				525	6.56	rs7588221	43507820	*THADA*	9.52E-04	6.25E-03	0.26	0.24
13				525	6.56	rs9309097	43407901	*THADA*	1.25E-03	8.20E-03	0.36	0.46
13				525	6.56	rs4128209	43408233	*THADA*	1.31E-03	8.59E-03	0.37	0.37
13				525	6.56	rs6730757	43395102	*THADA*	1.36E-03	8.96E-03	0.11	0.19
13				525	6.56	rs11690012	43395984	*THADA*	1.38E-03	9.03E-03	0.35	0.36
13				525	6.56	rs9309098	43408476	*THADA*	1.61E-03	1.06E-02	0.36	0.46
13				525	6.56	rs13414381	43420722	*THADA*	1.81E-03	1.19E-02	0.70	0.60
13				525	6.56	rs12712886	43552920	*THADA*	2.04E-03	1.34E-02	0.32	0.22
13				525	6.56	rs6716620	43488426	*THADA*	2.16E-03	1.42E-02	0.23	0.09
13				525	6.56	rs6544650	43470316	*THADA*	2.25E-03	1.48E-02	0.25	0.14
13				525	6.56	rs9309101	43483116	*THADA*	2.79E-03	1.83E-02	0.15	0.26
13				525	6.56	rs7557989	43484161	*THADA*	3.84E-03	2.52E-02	0.16	0.25
13				525	6.56	rs6732337	43316326	*THADA*	3.88E-03	2.55E-02	0.20	0.44
13				525	6.56	rs7599564	43363121	*THADA*	4.00E-03	2.62E-02	0.07	0.18
13				525	6.56	rs7605661	43478582	*THADA*	4.54E-03	2.98E-02	0.21	0.23
13				525	6.56	rs13414140	43524680	*THADA*	5.08E-03	3.34E-02	0.45	1.00
13				525	6.56	rs6726917	43607417	*THADA*	5.25E-03	3.44E-02	0.79	1.00
13				525	6.56	rs6761129	43589217	*THADA*	5.31E-03	3.48E-02	0.58	1.00
13				525	6.56	rs6722694	43423302	*THADA*	5.62E-03	3.69E-02	0.38	0.36
13				525	6.56	rs13026309	43355981	*THADA*	6.02E-03	3.95E-02	0.14	0.15
13				525	6.56	rs11690918	43327616	*THADA*	6.16E-03	4.04E-02	0.44	0.44
13				525	6.56	rs10196095	43425389	*THADA*	6.93E-03	4.55E-02	0.44	0.46
13				525	6.56	rs17031173	43732646	*PLEKHH2*	6.93E-03	4.55E-02	0.16	
13				525	6.56	rs6714067	43485704	*THADA*	7.15E-03	4.69E-02	0.18	0.24
14	rs243088	2	60422249	64	6.32	rs7594830	60430306	*BCL11A*	1.95E-04	1.23E-03	0.00	
14				64	6.32	rs243088	60422249	*BCL11A*	5.08E-04	3.20E-03	1.00	1.00
14				64	6.32	rs243018	60440211	*BCL11A*	2.68E-03	1.69E-02	0.36	0.84

14				64	6.32	rs7595905	60395357	*BCL11A*	5.33E-03	3.37E-02	0.01	0.25
14				64	6.32	rs243083	60427374	*BCL11A*	5.61E-03	3.54E-02	0.62	1.00
14				64	6.32	rs243090	60419483	*BCL11A*	5.73E-03	3.62E-02	0.62	1.00
14				64	6.32	rs10174120	60433784	*BCL11A*	6.37E-03	4.02E-02	0.27	0.09
14				64	6.32	rs243085	60426175	*BCL11A*	6.39E-03	4.03E-02	0.65	1.00
15	rs6712932	2	105204030	44	4.11	rs1545122	105219264	*GPR45*	4.30E-02	1.77E-01	0.06	0.08
16	rs1530559	2	135472099	558	9.82	rs16831351	135596535	*RAB3GAP1*	4.66E-04	4.57E-03	0.06	
16				558	9.82	rs16831005	135253802	*TMEM163, ACMSD*	1.87E-03	1.84E-02	0.03	
16				558	9.82	rs7561578	135776896	*ZRAN3B*	2.57E-03	2.52E-02	0.08	
16				558	9.82	rs16831046	135271460	*ACMSD*	3.64E-03	3.58E-02	0.02	
16				558	9.82	rs16831056	135288186	*ACMSD*	4.54E-03	4.46E-02	0.02	
17	rs7560163	2	151346182	222	6.73	rs6716109	151381845	*RND3*	2.08E-03	1.40E-02	0.12	
17				222	6.73	rs1519755	151409638	*RND3*	2.91E-03	1.96E-02	0.13	
17				222	6.73	rs12998083	151384420	*RND3*	3.34E-03	2.25E-02	0.12	
17				222	6.73	rs1850879	151406403	*RND3*	3.51E-03	2.37E-02	0.06	
17				222	6.73	rs1949525	151378138	*RND3*	5.79E-03	3.90E-02	0.04	
17				222	6.73	rs1949524	151372776	*RND3*	6.60E-03	4.45E-02	0.03	
18	rs7593730	2	160879700	211	4.69	rs13001911	160728949	*ITGB6*	3.50E-03	1.64E-02	0.08	0.17
19	rs13389219	2	165237122	205	5.23	rs12692738	165266498	*COBLL1*	1.99E-03	1.04E-02	0.65	0.33
19				205	5.23	rs355849	165334076	*COBLL1*	5.17E-03	2.71E-02	0.00	0.07
19				205	5.23	rs427059	165315200	*COBLL1*	8.75E-03	4.58E-02	0.00	0.07
20	rs560887	2	169471394	101	5.35	rs16856196	169479507	*G6PC2*	1.57E-02	8.38E-02	0.03	0.00
21	rs10497721	2	192622607	122	8.49	rs13418161	192672827	*TMEFF2*	3.76E-02	3.19E-01	0.02	
22	rs7578326	2	226728897	365	8.30	rs11684232	226630137	*IRS1*	1.50E-04	1.24E-03	0.00	0.05
22				365	8.30	rs1879946	226644683	*IRS1*	2.32E-04	1.93E-03	0.00	0.05
22				365	8.30	rs4503980	226623946	*IRS1*	2.65E-04	2.20E-03	0.00	0.05
22				365	8.30	rs6732584	226692506	*IRS1*	3.51E-04	2.91E-03	0.03	0.05
22				365	8.30	rs4675051	226699865	*IRS1*	3.78E-04	3.13E-03	0.00	0.05
22				365	8.30	rs16866837	226665382	*IRS1*	3.96E-04	3.28E-03	0.00	0.03
22				365	8.30	rs4673157	226641531	*IRS1*	5.25E-04	4.35E-03	0.00	0.05
22				365	8.30	rs13006806	226606872	*IRS1*	6.08E-04	5.05E-03	0.00	0.09
22				365	8.30	rs13018599	226610806	*IRS1*	9.47E-04	7.85E-03	0.00	0.09
22				365	8.30	rs16866804	226642252	*IRS1*	1.02E-03	8.50E-03	0.03	0.05
22				365	8.30	rs11688065	226666713	*IRS1*	1.28E-03	1.07E-02	0.05	0.03
22				365	8.30	rs1522813	226653426	*IRS1*	1.45E-03	1.20E-02	0.05	0.05
22				365	8.30	rs4675052	226700070	*IRS1*	1.46E-03	1.21E-02	0.02	0.05
22				365	8.30	rs4675041	226654679	*IRS1*	1.49E-03	1.24E-02	0.05	0.05
22				365	8.30	rs7606219	226679330	*IRS1*	1.75E-03	1.45E-02	0.05	0.03
22				365	8.30	rs1522822	226679810	*IRS1*	1.84E-03	1.52E-02	0.05	0.03
22				365	8.30	rs6713510	226742743	*IRS1*	2.18E-03	1.81E-02	0.34	0.43
22				365	8.30	rs2943644	226754586	*IRS1*	2.21E-03	1.84E-02	0.31	0.43
22				365	8.30	rs4645008	226747062	*IRS1*	2.27E-03	1.88E-02	0.34	0.43
22				365	8.30	rs4072096	226744847	*IRS1*	2.29E-03	1.90E-02	0.34	0.43
22				365	8.30	rs2943633	226763125	*IRS1*	2.64E-03	2.19E-02	0.31	0.43
22				365	8.30	rs7599280	226644498	*IRS1*	2.86E-03	2.37E-02	0.01	0.02
22				365	8.30	rs7587260	226644727	*IRS1*	2.86E-03	2.38E-02	0.00	0.05
22				365	8.30	rs2894586	226736977	*IRS1*	3.36E-03	2.79E-02	0.35	0.43
22				365	8.30	rs16866816	226649213	*IRS1*	3.65E-03	3.03E-02	0.01	0.02
22				365	8.30	rs4675039	226639570	*IRS1*	4.02E-03	3.33E-02	0.03	0.05
23	rs1801282	3	12368125	728	13.20	rs1186464	12462511	*PPARG*	1.35E-03	1.78E-02	0.00	0.00
23				728	13.20	rs12631819	12317861	*PPARG*	1.82E-03	2.40E-02	0.00	0.00
23				728	13.20	rs9809905	12475651	*PPARG*	2.21E-03	2.91E-02	0.20	0.02
23				728	13.20	rs2292101	12409901	*PPARG*	2.78E-03	3.67E-02	0.00	0.00
24	rs11715915	3	49430334	198	4.07	rs1865741	49337896	*USP4*	1.89E-02	7.70E-02	0.05	0.01
25	rs6784615	3	52481466	173	4.58	rs1011062	52481943	*NISCH*	1.68E-03	7.68E-03	1.00	1.00
25				173	4.58	rs11717574	52243286	*TWF2*	2.47E-03	1.13E-02	0.01	0.00
25				173	4.58	rs6793317	52274026	*WDR82*	6.80E-03	3.11E-02	0.00	0.00
25				173	4.58	rs4687614	52467125	*NISCH*	6.81E-03	3.12E-02	0.47	1.00
25				173	4.58	rs6445486	52481531	*NISCH*	6.82E-03	3.12E-02	1.00	1.00
25				173	4.58	rs9855470	52468315	*NISCH*	7.94E-03	3.64E-02	1.00	1.00
25				173	4.58	rs17052053	52309633	*GLYCTK*	9.13E-03	4.19E-02	0.00	0.00
26	rs358806	3	55288440	55	6.10	rs1373633	55280840	*CACNA2D3 , WNT5A*	8.99E-03	5.49E-02	0.01	
27	rs831571	3	64023337	260	8.78	rs7644517	64066005	*PRICKLE2*	3.57E-03	3.13E-02	0.01	0.01
28	rs4607103	3	64686944	122	5.90	rs4616635	64677315	*ADAMTS9*	1.22E-02	7.18E-02	0.12	0.71
29	rs1489100	3	76215254	82	2.67	rs4856003	76180584	*ZNF717*	5.75E-03	1.54E-02	0.27	0.33
29				82	2.67	rs1601982	76219398	*ZNF717*	1.35E-02	3.61E-02	0.30	0.52
29				82	2.67	rs7632524	76187309	*ZNF717*	1.40E-02	3.73E-02	0.29	0.45
29				82	2.67	rs10511039	76184447	*ZNF717*	1.66E-02	4.44E-02	0.29	0.45
30	rs2063640	3	103685735	635	13.06	rs9836986	103701039	*ZPLD1*	2.97E-02	3.88E-01	0.00	0.01
31	rs2715755	3	110164908	321	4.27	rs9849179	110147469	*GUCA1C*	2.10E-02	8.95E-02	0.35	0.40
32	rs11708067	3	124548468	186	5.57	rs11708067	124548468	*ADCY5*	1.12E-04	6.23E-04	1.00	1.00
32				186	5.57	rs9861425	124555573	*ADCY5*	4.04E-04	2.25E-03	0.09	0.24
32				186	5.57	rs2055168	124566288	*ADCY5*	4.42E-04	2.46E-03	0.03	
32				186	5.57	rs7613951	124553207	*ADCY5*	5.13E-04	2.85E-03	0.91	0.88
32				186	5.57	rs11717195	124565088	*ADCY5*	6.87E-04	3.82E-03	0.91	0.88
32				186	5.57	rs6807089	124546779	*ADCY5*	8.39E-04	4.67E-03	0.65	0.25
32				186	5.57	rs1456116	124567069	*ADCY5*	8.75E-04	4.87E-03	0.48	0.25

32				186	5.57	rs10934647	124613374	*ADCY5*	1.33E-03	7.38E-03	0.45	0.61
32				186	5.57	rs4630884	124551433	*ADCY5*	1.56E-03	8.66E-03	0.12	0.25
32				186	5.57	rs6798189	124578002	*ADCY5*	1.94E-03	1.08E-02	0.91	0.80
32				186	5.57	rs11708903	124548700	*ADCY5*	2.46E-03	1.37E-02	0.63	0.25
32				186	5.57	rs4470442	124550714	*ADCY5*	2.74E-03	1.53E-02	0.39	0.24
32				186	5.57	rs17361324	124613944	*ADCY5*	4.99E-03	2.78E-02	0.45	0.61
33	rs3773506	3	143913690	316	9.84	rs3821647	144006039	*TRPC1*	2.18E-03	2.15E-02	0.04	0.23
34	rs729511	3	144538397	101	7.16	rs12631011	144556168	*SLC9A9*	7.80E-03	5.58E-02	0.11	0.06
35	rs11920090	3	172200215	141	4.52	rs12488694	172227055	*SLC2A2*	9.40E-03	4.25E-02	0.14	0.03
35				141	4.52	rs12495549	172180254	*SLC2A2*	9.77E-03	4.42E-02	0.08	0.19
36	rs7630877	3	181144012	226	12.31	rs9832389	180999084	*PEX5L*	8.90E-04	1.10E-02	0.03	0.00
36				226	12.31	rs9847180	180998291	*PEX5L*	2.05E-03	2.52E-02	0.03	0.00
36				226	12.31	rs9850496	180997404	*PEX5L*	2.17E-03	2.67E-02	0.03	0.00
36				226	12.31	rs2339912	181049874	*PEX5L*	2.96E-03	3.64E-02	0.12	0.17
37	rs1470579	3	187011774	68	3.31	rs11705729	186989993	*IGF2BP2*	4.29E-04	1.42E-03	0.97	1.00
37				68	3.31	rs11711477	187009384	*IGF2BP2*	5.01E-04	1.66E-03	0.97	1.00
37				68	3.31	rs9859406	187017176	*IGF2BP2*	1.31E-03	4.34E-03	0.97	1.00
37				68	3.31	rs4481184	186988481	*IGF2BP2*	1.46E-03	4.85E-03	0.57	1.00
37				68	3.31	rs6769511	187012984	*IGF2BP2*	1.54E-03	5.09E-03	1.00	1.00
37				68	3.31	rs6808808	186983932	*IGF2BP2*	1.75E-03	5.79E-03	0.10	0.10
37				68	3.31	rs1470580	187011868	*IGF2BP2*	2.48E-03	8.21E-03	1.00	1.00
37				68	3.31	rs7633675	186993307	*IGF2BP2*	3.28E-03	1.09E-02	0.97	1.00
37				68	3.31	rs6444082	187018917	*IGF2BP2*	3.71E-03	1.23E-02	0.67	0.73
38	rs16861329	3	188149155	62	8.47	rs13100451	188135912	*ST6GAL1*	1.02E-02	8.68E-02	0.00	0.05
39	rs6815464	4	1299901	420	9.12	rs2290402	931518	*TMEM175*	6.70E-06	6.11E-05	0.00	0.01
39				420	9.12	rs3796610	893694	*GAK*	5.87E-04	5.35E-03	0.07	0.00
39				420	9.12	rs3775127	876061	*GAK*	6.23E-04	5.68E-03	0.02	0.02
39				420	9.12	rs3775119	883712	*GAK*	1.18E-03	1.08E-02	0.02	0.03
39				420	9.12	rs17165130	865339	*GAK*	1.40E-03	1.28E-02	0.02	0.02
39				420	9.12	rs3775129	865155	*GAK*	1.93E-03	1.76E-02	0.02	0.02
39				420	9.12	rs4974609	1346924	*UVSSA*	2.11E-03	1.92E-02	0.03	0.07
39				420	9.12	rs3736087	866318	*GAK*	2.70E-03	2.46E-02	0.02	0.03
39				420	9.12	rs2279184	872340	*GAK*	2.95E-03	2.69E-02	0.03	0.00
39				420	9.12	rs3733344	941040	*TMEM175*	3.18E-03	2.90E-02	0.00	0.06
39				420	9.12	rs6822424	909765	*GAK*	3.99E-03	3.64E-02	0.09	0.00
39				420	9.12	rs4690336	857242	*GAK*	4.07E-03	3.71E-02	0.02	0.02
39				420	9.12	rs3755959	891672	*GAK*	5.19E-03	4.73E-02	0.05	0.00
40	rs1801214	4	6353923	78	2.64	rs6446490	6375548	*PPP2R2C*	1.62E-01	4.28E-01	0.18	0.31
41	rs3822072	4	89960292	33	2.09	rs17014589	89945116	*FAM13A*	1.97E-02	4.12E-02	0.11	
42	rs9884482	4	106301085	164	7.95	rs3796927	106377665	*TET2*	2.48E-04	1.97E-03	0.02	
42				164	7.95	rs6825684	106304092	*TET2*	2.89E-04	2.30E-03	0.07	0.07
42				164	7.95	rs6843141	106375200	*TET2*	4.30E-04	3.42E-03	0.02	0.02
42				164	7.95	rs2133084	106433436	*TET2*	5.70E-04	4.53E-03	0.00	0.05
42				164	7.95	rs11728350	106297546	*TET2*	6.20E-04	4.93E-03	0.05	0.07
42				164	7.95	rs763480	106291046	*TET2*	9.43E-04	7.49E-03	0.06	0.07
42				164	7.95	rs7700148	106364879	*TET2*	1.14E-03	9.08E-03	0.02	
42				164	7.95	rs6533186	106433452	*TET2*	1.30E-03	1.03E-02	0.01	0.00
42				164	7.95	rs6533185	106433323	*TET2*	1.66E-03	1.32E-02	0.01	0.00
42				164	7.95	rs17035311	106285742	*TET2*	2.57E-03	2.04E-02	0.06	0.07
42				164	7.95	rs7679673	106280983	*TET2*	2.64E-03	2.10E-02	0.01	0.02
42				164	7.95	rs10488913	106268865	*TET2*	3.74E-03	2.97E-02	0.08	0.02
42				164	7.95	rs10017522	106343654	*TET2*	3.83E-03	3.04E-02	0.01	
42				164	7.95	rs10008189	106371014	*TET2*	3.84E-03	3.06E-02	0.01	
42				164	7.95	rs7698522	106338205	*TET2*	4.11E-03	3.27E-02	0.04	0.07
42				164	7.95	rs2189234	106294947	*TET2*	4.70E-03	3.74E-02	0.06	0.46
43	rs180730	4	122021240	163	5.73	rs1376108	122057654	*PRDM5*	2.33E-04	1.33E-03	0.02	
43				163	5.73	rs1450469	122074663	*PRDM5*	2.40E-04	1.38E-03	0.04	
43				163	5.73	rs6845189	122081149	*PRDM5*	7.57E-04	4.34E-03	0.04	
43				163	5.73	rs4639096	122042826	*PRDM5*	2.70E-03	1.55E-02	0.32	0.57
43				163	5.73	rs1868716	122083411	*PRDM5*	4.10E-03	2.35E-02	0.04	
43				163	5.73	rs11935242	122042005	*PRDM5*	8.71E-03	4.99E-02	0.01	0.39
44	rs7659604	4	122884964	123	5.99	rs4370153	122902457	*TMEM155*	9.71E-03	5.82E-02	0.04	0.20
45	rs6822892	4	157954125	169	4.76	rs7687696	157971696	*PDGFC*	1.13E-02	5.38E-02	0.03	0.15
46	rs3792615	4	164752251	749	20.82	rs13103455	164964345	*MARCH1*	5.47E-03	1.14E-01	0.02	0.01
47	rs4865796	5	53308421	91	4.55	rs6898870	53328864	*ARL15*	5.64E-03	2.57E-02	0.10	0.09
47				91	4.55	rs10805455	53338059	*ARL15*	6.87E-03	3.13E-02	0.14	0.07
47				91	4.55	rs3776726	53325809	*ARL15*	7.00E-03	3.18E-02	0.10	0.09
47				91	4.55	rs2448	53338111	*ARL15*	7.80E-03	3.55E-02	0.14	0.08
47				91	4.55	rs12659966	53328649	*ARL15*	8.29E-03	3.77E-02	0.10	0.08
47				91	4.55	rs12521454	53333061	*ARL15*	8.54E-03	3.89E-02	0.14	0.08
48	rs459193	5	55842508	65	6.38	rs459193	55842508	*ANKRD55-MAP3K1*	3.25E-03	2.07E-02	1.00	1.00
49	rs4457053	5	76460705	31	6.03	rs6861809	76481141	*ZBED3-AS1*	4.29E-02	2.58E-01	0.00	0.03
50	rs12518099	5	89581865	69	2.84	rs13355744	89566287	*CETN3*	9.04E-03	2.56E-02	0.00	
51	rs4869272	5	95565204	283	8.15	rs13177695	95433475	*GLRX*	3.64E-03	2.97E-02	0.04	0.00
52	rs1019503	5	96280573	220	2.53	rs2911132	96212744	*ERAP2*	6.14E-03	1.55E-02	0.01	0.01
52				220	2.53	rs2549803	96200685	*ERAP2*	6.19E-03	1.57E-02	0.01	0.01
52				220	2.53	rs2927614	96224235	*ERAP2*	1.77E-02	4.49E-02	0.05	0.08
53	rs2545523	5	165446786	168	8.58	rs10515916	165389114	*TENM2*	1.42E-02	1.22E-01	0.00	0.03

54	rs17762454	6	7158199	153	7.78	rs9502560	7122139	*RREB1*	1.57E-03	1.22E-02	0.06	0.05
54				153	7.78	rs9505057	7116406	*RREB1*	1.79E-03	1.39E-02	0.05	0.06
54				153	7.78	rs9405328	7111237	*RREB1*	2.43E-03	1.89E-02	0.05	0.06
54				153	7.78	rs6935691	7123538	*RREB1*	2.51E-03	1.95E-02	0.03	0.05
54				153	7.78	rs1285882	7101900	*RREB1*	4.29E-03	3.34E-02	0.05	0.08
55	rs10440833	6	20796100	315	10.34	rs4712523	20765543	*CDKAL1*	1.78E-03	1.84E-02	0.08	0.82
55				315	10.34	rs9460550	20827540	*CDKAL1*	3.14E-03	3.25E-02	0.07	0.60
55				315	10.34	rs9368224	20827211	*CDKAL1*	3.15E-03	3.26E-02	0.07	0.60
55				315	10.34	rs9358357	20827124	*CDKAL1*	3.40E-03	3.52E-02	0.07	0.60
55				315	10.34	rs6456368	20767785	*CDKAL1*	3.49E-03	3.61E-02	0.09	0.82
55				315	10.34	rs9460546	20771611	*CDKAL1*	3.77E-03	3.90E-02	0.09	0.82
55				315	10.34	rs4712522	20764779	*CDKAL1*	3.91E-03	4.05E-02	0.09	0.82
55				315	10.34	rs4712525	20770945	*CDKAL1*	4.07E-03	4.21E-02	0.09	0.82
55				315	10.34	rs9460544	20769508	*CDKAL1*	4.13E-03	4.27E-02	0.09	0.82
55				315	10.34	rs9368222	20794975	*CDKAL1*	4.26E-03	4.40E-02	0.94	0.97
56	rs6912327	6	34872900	319	4.09	rs13219307	35108641	*ANKS1A*	1.26E-02	5.14E-02	0.10	0.06
57	rs9470794	6	38214822	622	10.16	rs16890616	38462000	*BTBD9*	1.87E-02	1.90E-01	0.03	0.00
58	rs1535500	6	39292028	28	3.23	rs1544050	39387109	*KCNK17*	7.04E-02	2.27E-01	0.13	0.65
59	rs9472138	6	43919740	27	6.27	rs2274346	43927619	*VEGFA*	3.96E-02	2.48E-01	0.01	0.00
60	rs1048886	6	71345910	248	4.74	rs12111206	71304293	*FAM135A*	3.52E-02	1.67E-01	0.00	
61	rs2745353	6	127494628	134	2.42	rs2503112	127511737	*RSPO3*	6.50E-03	1.57E-02	0.03	0.00
61				134	2.42	rs6925791	127534330	*RSPO3*	1.30E-02	3.15E-02	0.02	0.00
61				134	2.42	rs11970168	127508939	*RSPO3*	1.39E-02	3.36E-02	0.02	0.00
61				134	2.42	rs1936808	127519134	*RSPO3*	1.79E-02	4.34E-02	0.01	0.00
62	rs2021966	6	132192132	39	5.52	rs6569759	132174809	*ENPP1*	1.01E-01	5.57E-01	0.01	0.48
63	rs642858	6	140315340	353	8.35	rs12662747	140403500	*LOC100132735*	2.71E-02	2.26E-01	0.02	0.00
64	rs17168486	7	14864807	111	4.34	rs978175	14837929	*DGKB*	2.18E-02	9.47E-02	0.00	0.06
65	rs2191349	7	15030834	99	6.75	rs7798129	15029610	*DGKB*	1.13E-03	7.64E-03	0.10	
65				99	6.75	rs2191348	15030780	*DGKB*	4.41E-03	2.98E-02	1.00	1.00
65				99	6.75	rs10267263	15020092	*DGKB*	5.84E-03	3.95E-02	0.10	0.00
65				99	6.75	rs6972880	15018689	*DGKB*	6.14E-03	4.15E-02	0.10	0.00
65				99	6.75	rs6972083	15018279	*DGKB*	6.32E-03	4.27E-02	0.10	0.00
65				99	6.75	rs10258074	15030741	*DGKB*	6.37E-03	4.30E-02	1.00	1.00
65				99	6.75	rs6978688	15019693	*DGKB*	6.50E-03	4.39E-02	0.10	0.00
65				99	6.75	rs10266967	15016709	*DGKB*	7.16E-03	4.84E-02	0.06	0.00
66	rs849134	7	28162747	95	5.65	rs498475	28222765	*JAZF1*	9.68E-04	5.47E-03	0.61	0.40
66				95	5.65	rs849327	28198982	*JAZF1*	2.77E-03	1.57E-02	0.61	0.54
66				95	5.65	rs849335	28190515	*JAZF1*	2.91E-03	1.65E-02	0.61	0.55
66				95	5.65	rs864745	28147081	*JAZF1*	5.54E-03	3.13E-02	1.00	1.00
66				95	5.65	rs849336	28190578	*JAZF1*	5.56E-03	3.14E-02	0.61	0.55
66				95	5.65	rs849142	28152416	*JAZF1*	5.60E-03	3.16E-02	0.80	1.00
66				95	5.65	rs1635852	28155936	*JAZF1*	7.39E-03	4.17E-02	1.00	0.95
66				95	5.65	rs849135	28162938	*JAZF1*	8.21E-03	4.64E-02	1.00	1.00
67	rs10486607	7	29151014	55	5.37	rs9639594	29145711	*CPVL*	2.18E-02	1.17E-01	0.04	0.51
68	rs4607517	7	44202193	24	3.61	rs12702070	44195757	*GCK*	1.25E-02	4.50E-02	0.00	0.03
69	rs1355037	7	50063624	338	5.01	rs12536329	49854295	*VWC2*	6.46E-03	3.24E-02	0.05	0.46
70	rs6943153	7	50759073	330	8.73	rs2237477	50712801	*GRB10*	2.55E-02	2.23E-01	0.05	0.08
71	rs1167800	7	75014132	39	6.16	rs1823616	74882566	*NSUN5P1*	4.52E-04	2.79E-03	0.04	
71				39	6.16	rs1668935	75014792	*HIP1*	5.75E-04	3.54E-03	0.17	0.75
71				39	6.16	rs6944634	74899705	*POM121C*	9.25E-04	5.70E-03	0.02	0.10
71				39	6.16	rs7805602	74997359	*PMS2P3*	1.44E-03	8.89E-03	NA	0.02
71				39	6.16	rs3973227	74973153	*SPDYE5, PMS2P3*	7.13E-03	4.39E-02	0.01	0.05
72	rs7636	7	100328013	68	4.19	rs7808329	100350168	*ACHE*	9.27E-02	3.88E-01	0.21	1.00
73	rs6467136	7	126952194	572	9.78	rs10276292	126990579	*GCC1*	1.19E-02	1.16E-01	0.12	0.11
74	rs1882095	7	129184880	151	6.86	rs692036	128987738	*NRF1*	3.03E-02	2.07E-01	0.08	0.29
75	rs972283	7	130117394	39	3.63	rs13234269	130079726	*KLF14*	7.79E-03	2.83E-02	0.27	0.98
76	rs983309	8	9215142	37	3.10	rs4841132	9221006	*LOC157273*	1.10E-03	3.40E-03	0.27	0.63
77	rs2722425	8	40603396	120	4.23	rs7812539	40597353	*ZMAT4*	8.92E-03	3.78E-02	0.03	0.00
77				120	4.23	rs4737158	40621557	*ZMAT4*	9.07E-03	3.84E-02	0.03	0.01
78	rs516946	8	41638405	169	8.61	rs10093583	41667160	*ANK1*	1.52E-03	1.31E-02	0.32	0.10
78				169	8.61	rs10504041	41584028	*AGPAT6*	2.51E-03	2.16E-02	0.00	0.32
78				169	8.61	rs17600159	41564666	*AGPAT6*	4.22E-03	3.64E-02	0.00	0.01
78				169	8.61	rs890220	41597518	*AGPAT6*	5.54E-03	4.77E-02	0.00	0.41
78				169	8.61	rs3802315	41647335	*ANK1*	5.68E-03	4.89E-02	0.53	1.00
79	rs896854	8	96029687	235	6.78	rs17359493	95926087	*INTS8*	1.49E-07	1.01E-06	0.10	0.21
79				235	6.78	rs16917079	95929347	*INTS8*	2.02E-07	1.37E-06	0.10	0.21
79				235	6.78	rs12056517	95946461	*INTS8*	5.27E-07	3.58E-06	0.12	0.21
79				235	6.78	rs11786088	95905316	*INTS8*	6.03E-07	4.09E-06	0.10	0.21
79				235	6.78	rs6986418	95931079	*INTS8*	1.05E-06	7.13E-06	0.14	0.11
79				235	6.78	rs16917081	95930461	*INTS8*	1.13E-06	7.64E-06	0.12	0.11
79				235	6.78	rs11782617	95944398	*INTS8*	3.68E-06	2.50E-05	0.12	0.21
79				235	6.78	rs16917102	95948211	*INTS8*	1.32E-05	8.98E-05	0.14	0.11
79				235	6.78	rs6471496	95885614	*INTS8*	5.65E-05	3.83E-04	0.13	0.10
79				235	6.78	rs11786389	95923996	*INTS8*	1.45E-04	9.82E-04	0.10	0.21
79				235	6.78	rs11783589	95915140	*INTS8*	1.86E-04	1.26E-03	0.10	0.21
79				235	6.78	rs16917093	95939296	*INTS8*	2.34E-04	1.59E-03	0.10	0.21

79				235	6.78	rs11781429	95925110	*INTS8*	2.78E-04	1.89E-03	0.10	0.21
79				235	6.78	rs11777353	95928359	*INTS8*	2.79E-04	1.89E-03	0.14	0.21
79				235	6.78	rs11784299	95919099	*INTS8*	2.91E-04	1.97E-03	0.10	0.21
79				235	6.78	rs6989591	95943756	*INTS8*	3.28E-04	2.22E-03	0.12	0.21
79				235	6.78	rs16917082	95931001	*INTS8*	3.45E-04	2.34E-03	0.14	0.21
79				235	6.78	rs4323442	96092162	*NDUFAF6*	5.07E-04	3.44E-03	0.00	0.20
79				235	6.78	rs7002940	96096568	*NDUFAF6*	5.40E-04	3.66E-03	0.00	0.17
79				235	6.78	rs2678829	96097356	*NDUFAF6*	5.52E-04	3.74E-03	0.11	0.33
79				235	6.78	rs7834966	95916372	*INTS8*	5.66E-04	3.84E-03	0.10	0.21
79				235	6.78	rs11775475	95974665	*CCNE2*	5.89E-04	4.00E-03	0.10	0.24
79				235	6.78	rs4075834	95902923	*INTS8*	6.44E-04	4.37E-03	0.10	0.21
79				235	6.78	rs12677346	95908045	*INTS8*	7.63E-04	5.18E-03	0.09	0.40
79				235	6.78	rs9297950	96094835	*NDUFAF6*	8.77E-04	5.95E-03	0.00	0.20
79				235	6.78	rs11783522	95955800	*INTS8*	1.02E-03	6.92E-03	0.08	0.24
79				235	6.78	rs7844932	95952408	*INTS8*	1.15E-03	7.79E-03	0.10	0.10
79				235	6.78	rs10956927	95920119	*INTS8*	1.32E-03	8.98E-03	0.10	0.21
79				235	6.78	rs7009940	95963976	*CCNE2*	1.48E-03	1.00E-02	0.12	0.11
79				235	6.78	rs7826196	96098178	*NDUFAF6*	1.48E-03	1.00E-02	0.00	0.20
79				235	6.78	rs6471505	96097801	*NDUFAF6*	1.83E-03	1.24E-02	0.00	0.20
79				235	6.78	rs10111300	96094807	*NDUFAF6*	2.06E-03	1.40E-02	0.00	0.20
79				235	6.78	rs11783941	95973371	*CCNE2*	2.43E-03	1.65E-02	0.12	0.13
79				235	6.78	rs16893774	95965152	*CCNE2*	2.43E-03	1.65E-02	0.11	0.24
79				235	6.78	rs7817415	95913920	*INTS8*	2.58E-03	1.75E-02	0.02	
79				235	6.78	rs3802191	96056760	*TP53INP1*	2.61E-03	1.77E-02	0.03	0.27
79				235	6.78	rs10098453	96098932	*NDUFAF6*	3.02E-03	2.05E-02	0.00	0.20
79				235	6.78	rs6471497	95918209	*INTS8*	3.28E-03	2.22E-02	0.09	0.34
79				235	6.78	rs4735328	95907614	*INTS8*	3.29E-03	2.23E-02	0.09	0.40
79				235	6.78	rs7005317	95916326	*INTS8*	3.41E-03	2.31E-02	0.09	0.39
79				235	6.78	rs16917117	95978674	*CCNE2*	3.43E-03	2.33E-02	0.11	0.24
79				235	6.78	rs11786021	95890145	*INTS8*	3.95E-03	2.68E-02	0.04	0.08
79				235	6.78	rs10113545	96098883	*NDUFAF6*	4.21E-03	2.86E-02	0.00	0.20
79				235	6.78	rs16917224	96099286	*NDUFAF6*	4.38E-03	2.97E-02	0.00	0.20
79				235	6.78	rs11779506	96144078	*NDUFAF6*	6.31E-03	4.28E-02	0.00	0.11
79				235	6.78	rs477653	95992208	*CCNE2, TP53INP1*	6.74E-03	4.57E-02	0.18	0.40
79				235	6.78	rs7812312	96089576	*NDUFAF6*	7.12E-03	4.83E-02	0.00	0.20
80	rs3802177	8	118254206	41	4.06	rs11558471	118254914	*SLC30A8*	1.15E-03	4.64E-03	1.00	0.90
80				41	4.06	rs3802177	118254206	*SLC30A8*	1.52E-03	6.16E-03	1.00	1.00
80				41	4.06	rs13266634	118253964	*SLC30A8*	5.54E-03	2.25E-02	1.00	1.00
81	rs7041847	9	4277466	107	5.96	rs4474077	4284674	*GLIS3*	4.43E-04	2.64E-03	0.02	0.00
81				107	5.96	rs10974436	4274369	*GLIS3*	5.29E-04	3.16E-03	0.08	0.10
81				107	5.96	rs4534176	4284807	*GLIS3*	7.56E-04	4.51E-03	0.03	0.00
81				107	5.96	rs6476840	4280897	*GLIS3*	9.83E-04	5.86E-03	0.04	
81				107	5.96	rs16921003	4264026	*GLIS3*	1.23E-03	7.31E-03	0.00	
81				107	5.96	rs2068980	4278367	*GLIS3*	1.52E-03	9.05E-03	0.02	
81				107	5.96	rs6476838	4277190	*GLIS3*	2.40E-03	1.43E-02	0.05	
81				107	5.96	rs10814908	4262449	*GLIS3*	3.62E-03	2.16E-02	0.05	0.34
81				107	5.96	rs6476837	4277179	*GLIS3*	3.77E-03	2.25E-02	0.02	
81				107	5.96	rs10117653	4281759	*GLIS3*	6.14E-03	3.66E-02	0.03	
82	rs17584499	9	8869118	17	2.35	rs7865131	8869086	*PTPRD*	1.30E-01	3.06E-01	0.05	0.02
83	rs649891	9	10420602	111	6.95	rs2382205	10372888	*PTPRD*	6.44E-02	4.48E-01	0.00	0.01
84	rs564398	9	22019547	156	7.32	rs16905599	22059144	*CDKN2B-AS*	8.15E-04	5.97E-03	0.02	0.06
84				156	7.32	rs7855660	22068305	*CDKN2B-AS*	4.44E-03	3.25E-02	0.00	
85	rs10965250	9	22123284	22	5.02	rs10757282	22123984	*CDKN2B-AS*	6.18E-03	3.10E-02	0.11	0.30
85				22	5.02	rs10217762	22123645	*CDKN2B-AS*	8.61E-03	4.32E-02	0.17	0.30
86	rs13292136	9	81141948	185	8.75	rs12380408	81119337	*TLE4*	1.14E-02	9.99E-02	0.03	0.05
87	rs2796441	9	83498768	66	5.30	rs1385041	83495482	*TLE1*	6.56E-02	3.47E-01	0.00	0.09
88	rs16913693	9	110720180	730	14.98	rs12335822	110640915	*IKBKAP*	3.67E-03	5.49E-02	0.02	0.00
89	rs4457406	9	114933365	21	2.78	rs10981630	114906094	*LINC00256B*	6.42E-03	1.79E-02	0.03	0.02
90	rs4740283	9	133438117	361	9.65	rs877373	133600060	*RAPGEF1*	7.84E-03	7.57E-02	0.00	0.03
91	rs3829109	9	138376587	72	2.30	rs3812550	138372700	*GPSM1*	3.90E-02	8.96E-02	0.47	0.37
92	rs12779790	10	12368016	132	6.16	rs12221133	12293603	*CDC123*	2.26E-03	1.39E-02	0.22	0.73
92				132	6.16	rs7896600	12295181	*CDC123*	2.47E-03	1.52E-02	0.22	0.73
92				132	6.16	rs11257655	12347900	*CDC123*	6.79E-03	4.18E-02	0.53	0.87
93	rs1802295	10	70601480	169	6.70	rs2855022	70519965	*SRGN*	2.11E-02	1.41E-01	0.30	0.25
94	rs12571751	10	80612637	119	9.80	rs10128172	80630316	*ZMIZ1*	4.43E-03	4.34E-02	0.00	0.00
95	rs1111875	10	94452862	175	5.92	rs1111875	94452862	*HHEX*	8.56E-03	5.07E-02	1.00	1.00
96	rs1416406	10	109034861	360	7.29	rs1416404	109068382	*SORCS1*	4.57E-03	3.33E-02	0.03	0.61
96				360	7.29	rs12219216	109068483	*SORCS1*	5.18E-03	3.77E-02	0.03	0.61
97	rs10885122	10	113032083	125	5.39	rs4244287	113013156	*ADRA2A*	1.99E-02	1.07E-01	0.12	0.20
98	rs7903146	10	114748339	46	3.54	rs7903146	114748339	*TCF7L2*	6.20E-24	2.20E-23	1.00	1.00
98				46	3.54	rs7901695	114744078	*TCF7L2*	2.83E-15	1.00E-14	0.59	0.94
98				46	3.54	rs7068741	114746248	*TCF7L2*	1.81E-12	6.40E-12	0.56	0.03
98				46	3.54	rs7069007	114746275	*TCF7L2*	2.24E-12	7.92E-12	0.38	0.03

98				46	3.54	rs12098651	114751709	*TCF7L2*	8.24E-10	2.92E-09	0.34	0.03
98				46	3.54	rs4132115	114745486	*TCF7L2*	4.88E-09	1.73E-08	0.56	0.03
98				46	3.54	rs4506565	114746031	*TCF7L2*	6.63E-09	2.35E-08	0.59	0.96
98				46	3.54	rs4319449	114759396	*TCF7L2*	2.52E-07	8.94E-07	0.13	0.04
98				46	3.54	rs11196187	114749435	*TCF7L2*	5.56E-07	1.97E-06	0.17	0.31
98				46	3.54	rs7907632	114807226	*TCF7L2*	1.44E-06	5.12E-06	0.47	0.03
98				46	3.54	rs7081912	114789081	*TCF7L2*	1.74E-06	6.17E-06	0.44	0.03
98				46	3.54	rs11196201	114793297	*TCF7L2*	1.27E-05	4.49E-05	0.07	0.24
98				46	3.54	rs11196192	114772277	*TCF7L2*	2.14E-05	7.59E-05	0.07	0.24
98				46	3.54	rs7092484	114750923	*TCF7L2*	2.22E-05	7.88E-05	0.21	0.03
98				46	3.54	rs4132670	114757761	*TCF7L2*	5.59E-05	1.98E-04	0.16	0.96
98				46	3.54	rs11196203	114795850	*TCF7L2*	6.84E-05	2.43E-04	0.08	0.05
98				46	3.54	rs17747324	114742493	*TCF7L2*	5.70E-04	2.02E-03	0.14	0.58
98				46	3.54	rs4074720	114738487	*TCF7L2*	3.08E-03	1.09E-02	0.10	0.50
98				46	3.54	rs7896811	114756707	*TCF7L2*	3.21E-03	1.14E-02	0.07	0.13
98				46	3.54	rs12266632	114754949	*TCF7L2*	9.91E-03	3.51E-02	0.08	0.31
98				46	3.54	rs4074718	114738607	*TCF7L2*	1.08E-02	3.83E-02	0.11	0.50
98				46	3.54	rs11196199	114786107	*TCF7L2*	1.31E-02	4.64E-02	0.06	0.05
99	rs10741243	10	132837952	85	5.52	rs4751326	132825482	*TCERG1L*	1.92E-02	1.06E-01	0.00	0.06
100	rs10770141	11	2150416	51	2.77	rs11564703	2163785	*TH, INS*	1.70E-06	4.71E-06	0.10	0.27
100				51	2.77	rs11043007	2160864	*TH, INS*	1.97E-06	5.46E-06	0.20	0.27
100				51	2.77	rs7396243	2162468	*TH, INS*	2.92E-06	8.10E-06	0.10	0.27
100				51	2.77	rs3922756	2176538	*TH, INS*	2.24E-04	6.21E-04	0.05	0.33
100				51	2.77	rs11564710	2156905	*TH, INS*	3.82E-04	1.06E-03	0.20	0.23
100				51	2.77	rs11043016	2162603	*TH, INS*	3.76E-03	1.04E-02	0.07	0.63
100				51	2.77	rs11564705	2163622	*TH, INS*	3.78E-03	1.05E-02	0.07	0.63
101	rs231362	11	2648047	37	5.87	rs231356	2661919	*KCNQ1*	2.91E-05	1.71E-04	0.17	0.24
101				37	5.87	rs231361	2648076	*KCNQ1*	3.97E-05	2.33E-04	0.16	0.36
101				37	5.87	rs463924	2674256	*KCNQ1*	9.41E-05	5.52E-04	0.18	0.21
101				37	5.87	rs231841	2680180	*KCNQ1*	1.90E-04	1.11E-03	0.22	0.27
101				37	5.87	rs231359	2651182	*KCNQ1*	4.82E-04	2.83E-03	0.17	0.31
101				37	5.87	rs231357	2661751	*KCNQ1*	5.65E-03	3.32E-02	0.16	0.46
102	rs163184	11	2803645	22	4.89	rs2283228	2806106	*KCNQ1*	6.17E-08	3.02E-07	0.03	0.03
102				22	4.89	rs2237892	2796327	*KCNQ1*	3.36E-07	1.65E-06	0.04	0.03
102				22	4.89	rs163182	2800792	*KCNQ1*	4.52E-04	2.21E-03	0.38	0.95
102				22	4.89	rs163177	2794989	*KCNQ1*	4.01E-03	1.96E-02	0.49	0.68
103	rs10500679	11	7112939	239	3.16	rs7924566	6999740	*NLRP14*	7.25E-04	2.29E-03	0.01	
103				239	3.16	rs1491831	7033922	*NLRP14*	4.91E-03	1.55E-02	0.01	
103				239	3.16	rs10732506	6988020	*ZNF214*	9.63E-03	3.04E-02	0.00	
103				239	3.16	rs7126582	6992474	*ZNF214*	9.71E-03	3.06E-02	0.00	
103				239	3.16	rs2638088	6976711	*ZNF214*	1.09E-02	3.43E-02	0.00	
104	rs2722769	11	11184950	53	5.27	rs2655084	11161857	*GALNTL4*	6.80E-02	3.59E-01	0.23	0.50
105	rs5215	11	17365206	168	3.14	rs11024158	17081744	*PIK3C2A*	1.67E-02	5.23E-02	0.00	0.26
106	rs9300039	11	41871942	682	9.99	rs7105457	41889169	*LRRC4C*	3.98E-04	3.97E-03	0.09	0.76
106				682	9.99	rs7120152	41880405	*LRRC4C*	5.81E-04	5.80E-03	0.31	0.80
106				682	9.99	rs10160319	41872304	*LRRC4C*	6.68E-04	6.67E-03	0.31	0.80
106				682	9.99	rs1385379	41776112	*LRRC4C*	3.83E-03	3.83E-02	0.01	0.02
106				682	9.99	rs1843246	41771426	*LRRC4C*	3.87E-03	3.86E-02	0.01	0.02
107	rs11605924	11	45829667	52	5.35	rs4756026	45790536	*SLC35C1*	8.88E-04	4.75E-03	0.01	0.15
108	rs7944584	11	47292896	436	8.80	rs11570097	47315519	*MYBPC3*	9.71E-03	8.55E-02	0.04	
109	rs174550	11	61328054	64	5.78	rs17156469	61383432	*FADS2*	1.07E-02	6.18E-02	0.02	
110	rs1552224	11	72110746	211	9.19	rs7121935	72173796	*STARD10*	4.97E-04	4.57E-03	0.01	0.07
111	rs1387153	11	92313476	53	3.76	rs10830963	92348358	*MTNR1B*	1.95E-02	7.31E-02	0.00	0.71
112	rs7107217	11	128978900	18	1.95	rs7107217	128978900	*BARX2*	4.55E-02	8.87E-02	1.00	1.00
113	rs11063069	12	4244634	11	2.02	rs4766227	4233611	*CCND2*	2.43E-01	4.90E-01	0.00	0.24
114	rs718314	12	26344550	89	4.62	rs1872994	26368311	*ITPR2*	1.60E-02	7.41E-02	0.02	0.12
115	rs10842994	12	27856417	108	8.49	rs11049111	27776072	*MRPS35*	1.55E-03	1.32E-02	0.03	0.42
115				108	8.49	rs11049103	27753348	*MRPS35*	2.97E-03	2.52E-02	0.05	0.42
116	rs12304921	12	49643809	208	5.84	rs12366756	49689473	*SLC11A2*	5.99E-04	3.50E-03	0.03	0.00
116				208	5.84	rs1048230	49672333	*SLC11A2*	6.44E-04	3.76E-03	0.03	0.00
116				208	5.84	rs11169654	49667102	*SLC11A2*	6.88E-04	4.02E-03	0.03	0.00
116				208	5.84	rs2285228	49669610	*SLC11A2*	7.02E-04	4.10E-03	0.03	0.00
116				208	5.84	rs10506298	49659425	*SLC11A2*	1.11E-03	6.49E-03	0.03	0.00
116				208	5.84	rs11169652	49665651	*SLC11A2*	4.12E-03	2.41E-02	0.04	0.00
116				208	5.84	rs10506299	49659281	*SLC11A2*	4.70E-03	2.75E-02	0.04	0.00
116				208	5.84	rs11169644	49652802	*SLC11A2*	4.85E-03	2.84E-02	0.02	0.00
117	rs1153188	12	53385263	118	7.70	rs10783639	53351381	*DCD*	1.60E-02	1.23E-01	0.12	0.07
118	rs2657879	12	55151605	44	3.77	rs2657880	55150037	*SPRYD4*	3.67E-03	1.38E-02	1.00	1.00
118				44	3.77	rs774211	55207206	*RBMS2*	4.58E-03	1.73E-02	0.66	0.81
118				44	3.77	rs2694917	55199131	*RBMS2*	6.62E-03	2.50E-02	0.34	0.75
119	rs1531343	12	64461161	116	5.05	rs343092	64537207	*HMGA2*	1.86E-06	9.38E-06	0.32	0.60
119				116	5.05	rs7953554	64487045	*HMGA2*	1.72E-05	8.71E-05	0.28	0.00
119				116	5.05	rs12298541	64592708	*HMGA2*	5.53E-05	2.79E-04	0.01	0.03
119				116	5.05	rs10878344	64590002	*HMGA2*	5.84E-05	2.95E-04	0.00	0.18
119				116	5.05	rs9668162	64555049	*HMGA2*	6.95E-05	3.51E-04	0.18	0.00
119				116	5.05	rs7133074	64584446	*HMGA2*	7.01E-05	3.54E-04	0.04	0.18
119				116	5.05	rs2293120	64506801	*HMGA2*	9.18E-05	4.64E-04	0.33	0.00
119				116	5.05	rs17101853	64571140	*HMGA2*	9.69E-05	4.89E-04	0.04	0.28
119				116	5.05	rs1480475	64544503	*HMGA2*	1.04E-04	5.24E-04	0.14	0.08

119				116	5.05	rs1460126	64548244	*HMGA2*	1.32E-04	6.67E-04	0.11	0.08
119				116	5.05	rs7979673	64513524	*HMGA2*	1.74E-04	8.80E-04	0.28	0.00
119				116	5.05	rs2446768	64525984	*HMGA2*	2.15E-04	1.08E-03	0.20	0.60
119				116	5.05	rs11175936	64514326	*HMGA2*	3.10E-04	1.56E-03	0.26	0.02
119				116	5.05	rs11834900	64533318	*HMGA2*	5.61E-04	2.83E-03	0.07	0.08
119				116	5.05	rs11175944	64542662	*HMGA2*	7.31E-04	3.69E-03	0.14	0.08
119				116	5.05	rs10878337	64517893	*HMGA2*	8.39E-04	4.24E-03	0.05	0.01
119				116	5.05	rs2272047	64523002	*HMGA2*	8.94E-04	4.52E-03	0.05	0.01
119				116	5.05	rs12310312	64521025	*HMGA2*	1.05E-03	5.32E-03	0.05	0.01
119				116	5.05	rs7964535	64511600	*HMGA2*	1.07E-03	5.38E-03	0.28	0.00
119				116	5.05	rs6581657	64498833	*HMGA2*	1.33E-03	6.73E-03	0.33	0.00
119				116	5.05	rs189339	64538561	*HMGA2*	1.33E-03	6.73E-03	0.00	0.55
119				116	5.05	rs7977687	64502340	*HMGA2*	1.42E-03	7.16E-03	0.32	0.00
119				116	5.05	rs7959396	64543214	*HMGA2*	1.59E-03	8.02E-03	0.25	0.00
119				116	5.05	rs1563834	64584298	*HMGA2*	1.63E-03	8.22E-03	0.00	0.18
119				116	5.05	rs7961706	64528165	*HMGA2*	3.37E-03	1.70E-02	0.07	0.08
119				116	5.05	rs6581658	64503612	*HMGA2*	3.77E-03	1.90E-02	0.32	0.00
119				116	5.05	rs7973574	64558999	*HMGA2*	7.35E-03	3.71E-02	0.03	0.00
119				116	5.05	rs11834895	64533277	*HMGA2*	8.12E-03	4.10E-02	0.00	0.00
120	rs1495377	12	69863368	329	8.65	rs1495372	69750995	*TSPAN8*	2.64E-03	2.29E-02	0.05	0.03
120				329	8.65	rs1533240	69745543	*TSPAN8*	3.69E-03	3.19E-02	0.07	0.03
120				329	8.65	rs11178555	69725080	*TSPAN8*	5.17E-03	4.47E-02	0.04	0.03
121	rs4760790	12	69921061	81	3.44	rs17109546	69925963	*TSPAN8*	1.67E-02	5.73E-02	0.00	
122	rs35767	12	101399699	106	4.58	rs12322412	101433718	*IGF1*	2.16E-02	9.88E-02	0.13	0.01
123	rs7957197	12	119945069	481	12.68	rs7132270	119888194	*HNF1A*	3.48E-04	4.41E-03	0.00	
124	rs10747083	12	131551691	18	1.79	rs1882314	131536377	*GALNT9, FBRSL1*	1.59E-01	2.84E-01	0.01	0.34
125	rs11619319	13	27385599	45	3.17	rs7983013	27376934	*PDX1*	1.70E-02	5.40E-02	0.00	
126	rs576674	13	32452302	59	7.22	rs576674	32452302	*KL*	1.22E-01	8.82E-01	1.00	1.00
127	rs2806739	13	53199780	467	7.26	rs17639850	53116226	*OLFM4*	1.06E-03	7.72E-03	0.27	0.05
128	rs2066219	13	68428665	487	12.11	rs9572044	68400858	*LOC338862*	4.65E-03	5.63E-02	0.00	0.12
129	rs1359790	13	79615157	154	11.08	rs9545330	79597167	*SPRY2*	1.01E-03	1.11E-02	0.02	0.12
129				154	11.08	rs17804768	79598890	*SPRY2*	1.28E-03	1.41E-02	0.01	0.12
130	rs2877832	14	26870017	409	7.96	rs11620859	27198434	*LOC100505 967*	1.55E-03	1.24E-02	0.03	0.07
131	rs2241119	14	80628718	63	2.78	rs10083503	80626837	*TSHR*	5.14E-02	1.43E-01	0.13	0.02
132	rs3783347	14	99909014	83	4.82	rs11624049	99922573	*WDR25*	6.15E-03	2.96E-02	0.03	0.06
133	rs730570	14	100212643	84	11.47	rs1802710	100270398	*DLK1*	9.38E-03	1.08E-01	0.00	0.00
134	rs2009833	15	47980845	108	6.72	rs16963036	47983835	*ATP8P4*	2.65E-02	1.78E-01	0.11	
135	rs7172432	15	60183681	320	4.37	rs2042610	60068209	*VPS13C*	5.18E-03	2.26E-02	0.01	0.24
135				320	4.37	rs11071635	59968800	*VPS13C*	7.14E-03	3.12E-02	0.01	0.17
135				320	4.37	rs17238252	60031573	*VPS13C*	9.81E-03	4.29E-02	0.03	0.23
136	rs1436953	15	60201306	65	4.22	rs4114259	60170457	*C2CD4A*	1.98E-01	8.33E-01	0.04	
137	rs7177055	15	75619817	330	4.46	rs11632729	75509061	*HMG20A*	8.89E-04	3.96E-03	0.01	0.23
137				330	4.46	rs16968835	75521316	*HMG20A*	9.01E-04	4.02E-03	0.01	0.23
137				330	4.46	rs7166293	75216908	*HMG20A*	2.60E-03	1.16E-02	0.01	0.08
137				330	4.46	rs11637488	75220516	*HMG20A*	2.68E-03	1.19E-02	0.01	0.08
137				330	4.46	rs11633318	75389753	*HMG20A*	3.75E-03	1.67E-02	0.01	0.09
137				330	4.46	rs7162362	75447148	*HMG20A*	5.53E-03	2.46E-02	0.01	0.07
137				330	4.46	rs11639314	75327157	*HMG20A*	6.96E-03	3.10E-02	0.04	0.44
137				330	4.46	rs1022172	75580414	*HMG20A*	7.94E-03	3.54E-02	0.38	0.93
137				330	4.46	rs2055903	75644046	*HMG20A*	8.71E-03	3.88E-02	0.00	0.06
138	rs11634397	15	78219277	55	2.60	rs12911387	78190181	*ZFAND6*	8.83E-02	2.30E-01	0.61	0.86
139	rs2028299	15	88175261	68	2.29	rs8034631	88223669	*AP3S2*	6.88E-05	1.58E-04	0.76	0.93
139				68	2.29	rs4932261	88231400	*AP3S2*	1.50E-04	3.43E-04	0.67	0.97
139				68	2.29	rs4932148	88195360	*AP3S2*	1.75E-04	4.00E-04	0.93	0.97
139				68	2.29	rs11638138	88229759	*AP3S2*	1.85E-04	4.25E-04	0.67	0.97
139				68	2.29	rs8031576	88181218	*AP3S2*	3.81E-04	8.73E-04	0.87	1.00
139				68	2.29	rs2028299	88175261	*AP3S2*	4.58E-04	1.05E-03	1.00	1.00
139				68	2.29	rs3784386	88238430	*AP3S2*	5.82E-04	1.34E-03	0.49	0.22
139				68	2.29	rs12912009	88230152	*AP3S2*	6.37E-04	1.46E-03	0.67	0.94
139				68	2.29	rs893617	88182282	*AP3S2*	7.49E-04	1.72E-03	0.90	1.00
139				68	2.29	rs1866476	88229547	*AP3S2*	7.85E-04	1.80E-03	0.67	0.97
139				68	2.29	rs11634658	88170152	*AP3S2*	9.74E-04	2.23E-03	0.33	0.93
139				68	2.29	rs3825866	88238642	*AP3S2*	9.81E-04	2.25E-03	0.49	0.22
139				68	2.29	rs986505	88219417	*AP3S2*	1.10E-03	2.52E-03	0.71	0.93
139				68	2.29	rs9920421	88169071	*AP3S2*	1.12E-03	2.56E-03	0.73	0.93
139				68	2.29	rs9920775	88169277	*AP3S2*	1.24E-03	2.84E-03	0.75	0.93
139				68	2.29	rs2351706	88185119	*AP3S2*	1.35E-03	3.10E-03	0.89	1.00
139				68	2.29	rs3784388	88238305	*AP3S2*	1.51E-03	3.47E-03	0.49	0.22
139				68	2.29	rs7174330	88235583	*AP3S2*	2.60E-03	5.97E-03	0.50	0.21
139				68	2.29	rs2043882	88240757	*AP3S2*	4.11E-03	9.42E-03	0.49	0.22
139				68	2.29	rs2043881	88240964	*AP3S2*	4.26E-03	9.78E-03	0.49	0.23
139				68	2.29	rs3853638	88244144	*AP3S2*	4.83E-03	1.11E-02	0.52	0.22
139				68	2.29	rs10852124	88231468	*AP3S2*	6.22E-03	1.43E-02	0.44	0.20
139				68	2.29	rs11629791	88250139	*AP3S2*	7.14E-03	1.64E-02	0.61	0.87
139				68	2.29	rs7111	88174877	*AP3S2*	7.71E-03	1.77E-02	0.93	1.00
139				68	2.29	rs12594808	88201888	*AP3S2*	9.67E-03	2.22E-02	0.93	0.97
139				68	2.29	rs1371135	88192274	*AP3S2*	1.09E-02	2.51E-02	0.93	1.00

139				68	2.29	rs17241560	88230206	*AP3S2*	1.52E-02	3.50E-02	0.28	0.21
139				68	2.29	rs17240471	88172175	*AP3S2*	1.65E-02	3.78E-02	0.07	0.33
139				68	2.29	rs3759831	88215646	*AP3S2*	1.67E-02	3.82E-02	0.72	0.93
139				68	2.29	rs3803534	88242874	*AP3S2*	1.98E-02	4.54E-02	0.72	0.94
139				68	2.29	rs2279849	88245643	*AP3S2*	2.09E-02	4.79E-02	0.72	0.90
140	rs8042680	15	89322341	121	9.84	rs41470046	89265552	*MAN2A2*	1.25E-02	1.23E-01	0.00	
141	rs11642841	16	52402988	71	3.00	rs16952523	52366195	*FTO*	1.35E-04	4.06E-04	0.03	0.02
141				71	3.00	rs4783820	52374285	*FTO*	3.21E-03	9.64E-03	0.01	0.02
141				71	3.00	rs12597786	52378808	*FTO*	7.61E-03	2.28E-02	0.01	0.02
141				71	3.00	rs1421085	52358455	*FTO*	1.41E-02	4.23E-02	1.00	0.75
142	rs7202877	16	73804746	201	3.23	rs8052132	73821638	*BCAR1*	3.04E-02	9.81E-02	0.00	
143	rs391300	17	2163008	174	2.66	rs216175	2114440	*SMG6*	1.10E-02	2.92E-02	0.12	0.04
144	rs1042522	17	7520197	22	5.92	rs8073498	7510423	*TP53*	2.93E-02	1.73E-01	0.15	0.01
145	rs4925115	17	17662182	223	3.62	rs9897309	17564713	*RAI1*	8.98E-03	3.25E-02	0.04	0.27
145				223	3.62	rs7208561	17556253	*RAI1*	1.09E-02	3.94E-02	0.00	0.23
145				223	3.62	rs8075153	17563391	*RAI1*	1.29E-02	4.66E-02	0.00	0.19
146	rs4430796	17	33172153	3	1.46	rs11651755	33173953	*HNF1B*	1.97E-02	2.88E-02	0.46	0.97
146				3	1.46	rs11658063	33177985	*HNF1B*	3.14E-02	4.60E-02	0.42	0.73
147	rs10460009	18	2938029	101	5.41	rs4797092	2920138	*LPIN2*	1.50E-02	8.08E-02	0.01	0.01
148	rs12970134	18	56035730	211	4.63	rs17782313	56002077	*MC4R*	4.77E-03	2.21E-02	0.20	0.86
148				211	4.63	rs921971	56012643	*MC4R*	6.82E-03	3.16E-02	0.78	1.00
148				211	4.63	rs8089364	56009809	*MC4R*	6.89E-03	3.19E-02	0.78	1.00
148				211	4.63	rs12964203	56054584	*MC4R*	7.45E-03	3.45E-02	0.94	1.00
148				211	4.63	rs8084834	55944569	*MC4R*	8.35E-03	3.86E-02	0.13	0.58
148				211	4.63	rs12969709	56010543	*MC4R*	8.52E-03	3.94E-02	0.78	0.96
148				211	4.63	rs1942880	55944189	*MC4R*	9.22E-03	4.27E-02	0.13	0.58
148				211	4.63	rs1942859	55950429	*MC4R*	9.48E-03	4.39E-02	0.13	0.58
149	rs12454712	18	58996864	1	1.00	rs12454712	58996864	*BCL2*	3.96E-01	3.96E-01	1.00	1.00
150	rs10401969	19	19268718	365	9.53	rs3761082	19110727	*TMEM161A*	3.61E-03	3.44E-02	0.00	0.01
151	rs3786897	19	38584848	155	5.67	rs12150970	38619311	*PEPD*	2.22E-03	1.26E-02	0.00	0.16
151				155	5.67	rs11084735	38618840	*PEPD*	6.09E-03	3.45E-02	0.01	0.04
151				155	5.67	rs11084734	38617428	*PEPD*	7.95E-03	4.50E-02	0.01	0.03
151				155	5.67	rs34910385	38582135	*PEPD*	8.11E-03	4.60E-02	0.04	0.13
152	rs472265	19	44272577	56	6.76	rs12609418	44309292	*PAK4*	5.22E-02	3.53E-01	0.06	0.14
153	rs8108269	19	50850353	13	2.39	rs10415769	50850483	*GIPR*	2.17E-01	5.18E-01	1.00	1.00
154	rs2302593	19	50888474	98	5.76	rs2302593	50888474	*QPCTL*	1.72E-01	9.89E-01	1.00	1.00
155	rs6113722	20	22505099	569	12.32	rs1203880	22490023	*FOXA2*	1.63E-03	2.00E-02	0.14	0.71
155				569	12.32	rs1203868	22480012	*FOXA2*	1.73E-03	2.13E-02	0.08	0.74
155				569	12.32	rs1203877	22489476	*FOXA2*	1.74E-03	2.14E-02	0.14	0.71
155				569	12.32	rs1203889	22494921	*FOXA2*	2.12E-03	2.61E-02	0.14	0.71
156	rs6072275	20	39177319	413	6.20	rs3795131	39203389	*PLCG1*	2.69E-03	1.67E-02	0.02	0.04
156				413	6.20	rs6102274	39154691	*TOP1*	4.58E-03	2.84E-02	0.01	
156				413	6.20	rs16989561	39176664	*TOP1*	4.71E-03	2.92E-02	0.01	
156				413	6.20	rs909802	39370229	*ZHX3*	5.99E-03	3.71E-02	0.02	0.27
156				413	6.20	rs6129760	39179817	*TOP1*	6.08E-03	3.77E-02	0.03	0.05
156				413	6.20	rs4812493	39382799	*ZHX3, LPIN3*	7.00E-03	4.34E-02	0.02	0.27
156				413	6.20	rs6093461	39326780	*ZHX3*	8.00E-03	4.96E-02	0.02	0.27
157	rs4812829	20	42422681	178	4.12	rs2144908	42419131	*HNF4A*	1.48E-03	6.08E-03	1.00	1.00
157				178	4.12	rs4812829	42422681	*HNF4A*	1.91E-03	7.87E-03	1.00	1.00
157				178	4.12	rs6031489	42327317	*GDAP1L1*	3.26E-03	1.34E-02	0.27	0.65
157				178	4.12	rs6017306	42329982	*GDAP1L1*	3.40E-03	1.40E-02	0.27	0.65
157				178	4.12	rs16988991	42423191	*HNF4A*	5.46E-03	2.25E-02	1.00	1.00
158	rs2833610	21	32307057	53	2.41	rs2248975	32296672	*HUNK*	5.12E-03	1.23E-02	0.55	0.10
158				53	2.41	rs3787694	32295043	*HUNK*	1.29E-02	3.12E-02	0.55	0.10
158				53	2.41	rs3746856	32294620	*HUNK*	1.46E-02	3.51E-02	0.55	0.10
158				53	2.41	rs16988780	32309829	*HUNK*	1.88E-02	4.52E-02	0.31	

Abbreviation: Chr, chromosome

[a]Index SNPs previously reported to be associated with type 2 diabetes or glucose homeostasis

[b] total number of SNPs in a locus bound by SNPs at 1000 Genomes CEU data with $r^2 \geq 0.3$ with the index SNPs within 500 kb

[c]SNPs with unadjusted *P* value < 0.05 in locus-wide analysis

[d]unadjusted *P* value

[e]*P* value adjusted for number of effective SNP per locus

[f]r^2 between regional SNP and index SNP using ASW and CEU data
