# Supplementary material for: Meta-Analysis of Genome-Wide Association Studies in African Americans Provides Insights into the Genetic Architecture of Type 2 Diabetes
Source: PLoS Genet. 2014 Aug 7;10(8):e1004517. doi: 10.1371/journal.pgen.1004517 (PMC4125087; doi:10.1371/journal.pgen.1004517)
Supplement: Table S7 — BMI-adjusted association for SNPs from stage 1 GWAS meta-analysis selected for replication. (PDF) [file pgen.1004517.s011.pdf]

Table S7. BMI-adjusted association for SNPs from stage 1 GWAS meta-analysis selected for replication

Chr	Position	SNP	Locus	Alleles[a]	RAF	OR (95% CI)[b]	*P*	P_{het}	N
1	239254905	rs679992	*RGS7*	T/C	0.50	1.13(1.08-1.18)	6.53E-07	1.56E-02	22033
2	30921523	rs12613372	*CAPN13-GALNT14*	G/C	0.07	1.27(1.16-1.39)	4.33E-07	1.45E-01	21059
3	169527567	rs9290337	*LOC389174*	G/A	0.27	1.14(1.07-1.23)	1.24E-04	8.45E-01	13773
3	178198612	rs1905499	*TBL1XR1*	G/T	0.59	1.11(1.06-1.16)	1.89E-05	5.83E-01	22949
3	178203474	rs1905496	*TBL1XR1*	A/G	0.59	1.11(1.06-1.16)	1.78E-05	5.76E-01	22890
4	931518	rs2290402	*TMEM175*	T/C	0.05	1.35(1.19-1.54)	4.77E-06	7.01E-01	18708
6	31455430	rs2244020	*HLA-B*	G/A	0.69	1.11(1.06-1.17)	3.34E-05	3.16E-02	22875
6	121716077	rs4946566	*GJA1*	G/C	0.03	1.4(1.21-1.62)	6.96E-06	4.73E-01	17184
6	169234624	rs12175557	*SMOC2*	A/G	0.02	1.54(1.27-1.86)	8.36E-06	8.54E-01	17925
7	43286956	rs9648079	*HECW1*	G/A	0.74	1.16(1.09-1.23)	1.05E-06	9.57E-01	21164
7	43287119	rs10231619	*HECW1*	T/C	0.74	1.15(1.09-1.22)	1.79E-06	9.69E-01	21521
7	43292722	rs4724195	*HECW1*	T/C	0.71	1.14(1.08-1.2)	3.70E-06	5.11E-01	22889
7	43293128	rs10272268	*HECW1*	C/T	0.71	1.14(1.08-1.2)	3.02E-06	5.90E-01	22935
7	43293209	rs10951720	*HECW1*	G/C	0.71	1.14(1.08-1.2)	3.14E-06	4.40E-01	22949
7	43293220	rs10951721	*HECW1*	C/T	0.71	1.14(1.08-1.2)	2.40E-06	5.03E-01	22931
7	43294434	rs10242062	*HECW1*	T/C	0.69	1.14(1.08-1.2)	8.19E-07	4.83E-01	22951
7	43296714	rs7794383	*HECW1*	T/C	0.74	1.15(1.08-1.22)	3.49E-06	9.61E-01	21183
8	68775944	rs7003257	*CPA6*	T/C	0.73	1.14(1.07-1.21)	1.23E-05	7.97E-01	21194
8	95905316	rs11786088	*INTS8*	T/C	0.22	1.13(1.07-1.2)	1.54E-05	7.56E-01	22025
8	95926087	rs17359493	*INTS8*	G/A	0.22	1.14(1.08-1.2)	4.25E-06	7.95E-01	22938
8	95929347	rs16917079	*INTS8*	G/A	0.21	1.14(1.08-1.2)	6.22E-06	8.33E-01	22944
8	95930461	rs16917081	*INTS8*	G/A	0.25	1.13(1.07-1.19)	1.20E-05	4.01E-01	22919
8	95931079	rs6986418	*INTS8*	G/A	0.25	1.13(1.07-1.19)	1.11E-05	5.18E-01	22955
8	95944398	rs11782617	*INTS8*	T/G	0.21	1.12(1.06-1.19)	5.32E-05	8.37E-01	22803
8	95946461	rs12056517	*INTS8*	T/C	0.22	1.13(1.07-1.19)	1.49E-05	7.24E-01	22934
8	135126458	rs2168707	*ZFAT*	G/A	0.11	1.24(1.12-1.36)	1.40E-05	3.34E-01	13750
9	129871150	rs10739706	*SLC25A25*	G/A	0.62	1.13(1.08-1.19)	6.78E-07	3.37E-01	22913
9	130111543	rs2231645	*TRUB2*	A/T	0.66	1.15(1.08-1.23)	1.20E-05	5.12E-01	16111
9	130111544	rs2231644	*TRUB2*	C/T	0.66	1.16(1.08-1.23)	1.06E-05	5.00E-01	16811
10	114744078	rs7901695	*TCF7L2*	C/T	0.45	1.22(1.16-1.28)	1.88E-16	4.05E-01	22948
10	114745486	rs4132115	*TCF7L2*	T/G	0.15	1.25(1.17-1.33)	1.06E-11	9.63E-03	22813
10	114746031	rs4506565	*TCF7L2*	T/A	0.45	1.17(1.12-1.23)	5.86E-11	1.73E-03	22247
10	114746248	rs7068741	*TCF7L2*	T/C	0.15	1.3(1.21-1.39)	2.07E-14	3.69E-01	21180
10	114746275	rs7069007	*TCF7L2*	C/G	0.11	1.31(1.22-1.41)	4.12E-13	3.05E-01	22951
10	114748339	rs7903146	*TCF7L2*	T/C	0.30	1.36(1.28-1.44)	3.01E-26	2.11E-01	20274
10	114749435	rs11196187	*TCF7L2*	A/G	0.07	1.36(1.2-1.54)	7.42E-07	2.96E-01	15831
10	114751709	rs12098651	*TCF7L2*	A/G	0.19	1.27(1.18-1.35)	6.90E-12	7.78E-01	20274
10	114759396	rs4319449	*TCF7L2*	G/T	0.09	1.32(1.2-1.46)	1.83E-08	4.31E-01	19735
10	114789081	rs7081912	*TCF7L2*	A/G	0.16	1.21(1.13-1.3)	5.25E-08	7.34E-01	20274
10	114807226	rs7907632	*TCF7L2*	G/A	0.16	1.22(1.13-1.31)	6.62E-08	7.81E-01	20274
11	2135246	rs3842770	*INS-IGF2*	A/G	0.23	1.18(1.11-1.25)	8.18E-08	7.16E-01	19705
11	2160864	rs11043007	*TH-ASCL2*	G/T	0.81	1.25(1.15-1.36)	4.29E-07	7.56E-01	19654
11	2162468	rs7396243	*TH-ASCL2*	G/T	0.80	1.25(1.14-1.36)	4.40E-07	7.89E-01	19654
11	2163785	rs11564703	*TH-ASCL2*	G/A	0.80	1.25(1.15-1.36)	4.23E-07	5.42E-01	19654
11	2661919	rs231356	*KCNQ1*	T/A	0.27	1.15(1.08-1.22)	2.55E-05	9.77E-01	19654
11	2796327	rs2237892	*KCNQ1*	C/T	0.90	1.24(1.14-1.34)	2.84E-07	3.75E-01	22227
11	2806106	rs2283228	*KCNQ1*	A/C	0.89	1.24(1.15-1.34)	4.67E-08	2.08E-01	22330
12	64451469	rs12049974	*HMGA2*	T/A	0.42	1.12(1.06-1.19)	2.79E-05	3.88E-01	20274
12	64537207	rs343092	*HMGA2*	T/G	0.81	1.18(1.1-1.26)	7.72E-07	9.16E-01	22927
15	58155498	rs335810	*ANXA2*	A/C	0.69	1.12(1.07-1.19)	1.43E-05	5.84E-01	21097
16	59250689	rs1423882	*GNPATP*	A/G	0.34	1.12(1.07-1.18)	7.67E-06	6.68E-01	22846

Abbreviations: Chr, chromosome; RAF, risk allele frequency; OR, odds ratio; CI, confidence interval; P_{het}, heterogeneity *P* value

[a]Alleles are ordered as risk allele/other allele aligned to the forward strand of NCBI Build 36

[b]Odds ratio are reported with respect to the risk allele
