# Supplementary material for: Meta-Analysis of Genome-Wide Association Studies in African Americans Provides Insights into the Genetic Architecture of Type 2 Diabetes
Source: PLoS Genet. 2014 Aug 7;10(8):e1004517. doi: 10.1371/journal.pgen.1004517 (PMC4125087; doi:10.1371/journal.pgen.1004517)
Supplement: Table S8 — Expression Quantitative Trait Loci (eQTL) analysis for the genome-wide significant SNPs for T2D. Results are shown for suggestive evidence of cis-association (P<0.05) between the genome-wide significant SNPs and their proxies with the genes within 1 Mb of the associated SNPs. (PDF) [file pgen.1004517.s012.pdf]

Table S8. Expression Quantitative Trait Loci (eQTL) analysis for the genome-wide significant SNPs for T2D. Results are shown for suggestive evidence of *cis*-association ($P < 0.05$) between the genome-wide significant SNPs and their proxies with the genes within 1Mb of the associated SNPs.

	Associated SNPs[a]				Proxy SNPs[b]								
**Locus**	**Chr**	**Position**	**SNP**	**Alleles[c]**	**SNP**	**Alleles**	**Source**	**Tissue**	**Probe**	**eQTL**	**Beta[d]**	**SE**	P_{eQTL}
HLA	6	31455430	rs2244020	G/A	rs2244020	G/A	Genevar-CEU	LCL	GI_13376873-S		-0.04	0.01	4.28E-03
HLA	6	31455430	rs2244020	G/A	rs2244020	G/A	Genevar-CEU	LCL	GI_24475831-S		-0.24	0.11	2.82E-02
HLA	6	31455430	rs2244020	G/A	rs2244020	G/A	Genevar-CEU	LCL	GI_25952110-S		-0.18	0.09	4.57E-02
HLA	6	31455430	rs2244020	G/A	rs2244020	G/A	Genevar-CEU	LCL	GI_5454157-S		0.18	0.07	1.43E-02
HLA	6	31455430	rs2244020	G/A	rs2244020	G/A	Genevar-YRI	LCL	GI_13376875-S		0.04	0.02	3.89E-02
HLA	6	31455430	rs2244020	G/A	rs2244020	G/A	Genevar-YRI	LCL	GI_45505160-I		0.03	0.01	3.65E-02
HLA	6	31455430	rs2244020	G/A	rs2244020	G/A	Genevar-YRI	LCL	GI_45580710-I		-0.10	0.03	1.69E-03
TCF7L2	10	114748339	rs7903146	T/C	rs7903146	C/T	MuTHER	FAT	ILMN_1739659	*ZDHHC6*	-0.03	0.01	9.90E-03
TCF7L2	10	114748339	rs7903146	T/C	rs7903146	C/T	MuTHER	LCL	ILMN_1734096	*DCLRE1A*	0.02	0.01	3.17E-02
INS-IGF2	11	2135246	rs3842770	A/G	rs3842770	A/G	Genevar-YRI	LCL	GI_32479524-I	*INS-IGF2*	-0.04	0.02	5.46E-03
KCNQ1	11	2661919	rs231356	T/A	rs231356	A/T	MuTHER	FAT	ILMN_1699867	*IGF2*	0.03	0.02	3.41E-02
KCNQ1	11	2661919	rs231356	T/A	rs231356	A/T	MuTHER	FAT	ILMN_2148527	*H19*	0.08	0.03	2.90E-03
KCNQ1	11	2661919	rs231356	T/A	rs231356	A/T	MuTHER	FAT	ILMN_2356578	*TH*	-0.01	0.01	3.44E-02
KCNQ1	11	2661919	rs231356	T/A	rs231356	A/T	MuTHER	LCL	ILMN_1688569	*KCNQ1*	-0.01	0.01	3.22E-02
KCNQ1	11	2806106	rs2283228	A/C	rs2283228	A/C	Genevar-CEU	LCL	GI_21071005-S	*KCNQ1*	0.18	0.08	2.41E-02
KCNQ1	11	2806106	rs2283228	A/C	rs2283228	A/C	Genevar-CEU	LCL	GI_34734072-I	*KCNQ1*	-0.05	0.02	1.16E-02
KCNQ1	11	2806106	rs2283228	A/C	rs2283228	A/C	Genevar-YRI	LCL	GI_32479526-I	*KCNQ1*	0.03	0.01	4.98E-02

Abbreviations: Chr, chromosome; eQTL, expression quantitative trait loci; SE, standard error

[a] Genome-wide significant SNPs

[b] Proxy SNPs had $r^2 \geq 0.8$ with the genome-wide significant SNPs in the 1000 Genomes ASW data

[c] Associated SNP alleles are ordered as risk allele/other allele

[d] effect sizes of gene expression are reported with respect to the first allele for proxy SNPs
