# Supplementary material for: Meta-Analysis of Genome-Wide Association Studies in African Americans Provides Insights into the Genetic Architecture of Type 2 Diabetes
Source: PLoS Genet. 2014 Aug 7;10(8):e1004517. doi: 10.1371/journal.pgen.1004517 (PMC4125087; doi:10.1371/journal.pgen.1004517)
Supplement: Table S9 — Putative regulatory SNPs predicted from the ENCODE project for the genome-wide significant SNPs and their proxies at TCF7L2, INS-IGF2, KCNQ1 and HMGA2. (PDF) [file pgen.1004517.s013.pdf]

Table S9. Putative regulatory SNPs predicted from the ENCODE project for the genome-wide significant SNPs and their proxies at *TCF7L2*, *INS-IGF2*, *KCNQ1* and *HMGA2*.

Locus	Chr	Position	Associated SNP[a]	Proxy SNP[b]	Proteins bound[c]	Motif changed[d]
TCF7L2	10	114758349	rs7903146	rs34872471		TEF
TCF7L2	10	114758349	rs7903146	rs7903146		Pou3f2
KCNQ1	11	2849530	rs2283228	rs2237892		SREBP
KCNQ1	11	2849530	rs2283228	rs2237897	CTCF, POL2, RAD21, SMC3, TFAP2A, TFAP2C, POLR2A, HNF4A	
KCNQ1	11	2849530	rs2283228	rs74046911	POL2, TFAP2A, TFAP2C, POLR2A, HNF4A	
HMGA2	12	66250940	rs343092	rs971779		Pou2f1, FOXP1
HMGA2	12	66250940	rs343092	rs2583939		TAL1::TCF3
HMGA2	12	66250940	rs343092	rs2257883		Mef2
HMGA2	12	66250940	rs343092	rs343092	RFX3	
HMGA2	12	66250940	rs343092	rs343093		p53

Abbreviations: Chr, chromosome

[a]Most strongly associated SNPs identified from genome-wide significant loci

[b] Proxy SNPs had $r^2 \geq 0.8$ with the best SNPs in the 1000 Genomes ASW data

[c] Protein binding site identified through ChIP-seq, and [d] predicted motif change through position weight matrices for proxy SNPs using HaploReg [30] and RegulomeDB [64]
