# Supplementary material for: Meta-Analysis of Genome-Wide Association Studies in African Americans Provides Insights into the Genetic Architecture of Type 2 Diabetes
Source: PLoS Genet. 2014 Aug 7;10(8):e1004517. doi: 10.1371/journal.pgen.1004517 (PMC4125087; doi:10.1371/journal.pgen.1004517)
Supplement: Text S1 — Description of GWAS and replication studies. (PDF) [file pgen.1004517.s014.pdf]

## Description of Stage 1 Genome-wide Association Studies

### *Atherosclerosis Risk in Communities (ARIC) Study*

The ARIC study is a prospective population-based study of atherosclerosis and cardiovascular disease that included 15,792 participants (27% African Americans) aged 45-64 years at baseline visit between years 1987-1989 from four US communities [67]. In this study, 955 T2D subjects diagnosed at any of the baseline or follow-up visits and 414 subjects with normal glucose tolerance (NGT) at all visits were included. All subjects were self-reported African Americans recruited from two communities (Jackson, MS and Forsyth, NC).

### *Coronary Artery Risk Development in Young Adults (CARDIA)*

The CARDIA study is a prospective multi-center investigation of the natural history and etiology of cardiovascular disease that included 5,115 participants (52% African Americans) aged 18-30 years at baseline visit between years 1985-1986 from four US communities (Birmingham, AL; Chicago, IL; Minneapolis, MN and Oakland, CA) [68]. Follow-up examinations occurred at years 2, 5, 7, 10, 15, and 20. In this study, 94 T2D subjects diagnosed at any visits and 654 subjects with NGT at all visits were included. All subjects were self-reported African Americans.

### *Cleveland Family Study (CFS)*

The CFS study is a prospective family-based study originally designed to study the risk factors for sleep apnea. In total, 2,534 subjects (46% African Americans) from 352 families in the Cleveland, Ohio metropolitan area were examined for up to four visits over a period of 16 years between years 1990-2006 [69]. In this study, 81 T2D and 98 NGT unrelated subjects at the fourth visit were included. All subjects were self-reported African Americans.

### *Cardiovascular Health Study (CHS)*

The CHS is a prospective population-based cohort study of risk factors for coronary heart disease and stroke in adults ≥65 years conducted across four field centers (Forsyth County, NC; Sacramento County, CA; Washington County, MD; and Pittsburgh, PA) [70]. The original predominantly Caucasian cohort of 5,201 subjects was recruited in 1989-1990 from random samples of the Medicare eligibility lists; subsequently, an additional predominantly African-

American cohort of 687 subjects was enrolled. DNA was extracted from blood samples drawn on all participants at their baseline examination. Genotyping was performed at the General Clinical Research Center's Phenotyping/Genotyping Laboratory at Cedars-Sinai using the Illumina HumanOmni1-Quad_v1 BeadChip system on 844 African American CHS participants who consented to genetic testing and had DNA available for genotyping. In this study, 226 T2D diagnosed at any visits and 474 subjects with NGT at all visits were included.

***Family Heart Study (FamHS)***

The Family Heart Study began in 1992 and subjects were recruited for an extensive clinical examination during the years 1994-1996. A second visit approximately 8 years later was conducted, in which a sample of African American families (N=624) was recruited at the University of Alabama in Birmingham field center [71]. The primary purpose of the visit was to obtain two sequential cardiac multidetector CT (MDCT) exams for individual coronary arteries and the aorta to estimate subclinical atherosclerosis burden as calcification. Measurements of the most important CHD risk factors in the lipid, glucose metabolism, blood pressure, and anthropometry domains were also assessed, along with medical history and medication use. In this study, 146 T2D cases and 322 controls were included. All subjects were self-reported African Americans.

***Family Investigation of Nephropathy in Diabetes (FIND)***

FIND is a multicenter study designed to identify genetic determinants of diabetic nephropathy in four ethnic groups including African Americans, European Americans, Mexican Americans, and American Indians [72]. Participants are entered into either of two main protocols (Family and MALD) and a retinopathy sub-study. The FIND Family protocol ascertained families of probands with diabetic nephropathy who had either two living parents or a diabetic sibling. The MALD protocol enrolled African American and Mexican American individuals with nephropathy, their offspring, and unrelated control individuals. Probands and family members are considered as having T2D if currently or previously treated with insulin, oral hypoglycemic drugs, or both. For diabetic subjects not treated with drugs or subjects with no history of diabetes, plasma hemoglobin $A_{1c}$ ($HbA_{1c}$) levels or, if possible, fasting plasma glucose concentrations were measured at the time of study entry. $HbA_{1c}$ ≥6.0% suggested diabetes and

was followed with plasma glucose testing to confirm a diagnosis of diabetes. In this study, 754 unrelated self-reported African American T2D cases were combined with 920 cases and 801 controls from the WFSM study for association analysis. Ascertainment criteria for cases were similar between the two studies.

***Genetic Study of Atherosclerosis Risk (GeneSTAR)***

GeneSTAR is a longitudinal family-based study to examine the determinants of incident atherosclerotic coronary heart disease, stroke, and vascular disease among apparently healthy first degree adult relatives of hospitalized probands with documented coronary disease prior to 60 years of age [73]. Between 1990 and 2006, GeneSTAR enrolled 1,342 asymptomatic, apparently healthy African American siblings and offspring of the proband and his/her siblings, as well as co-parents of the offspring, all between the ages of 21 and 80. In this study, 228 T2D cases diagnosed at any visit and 620 control subjects with NGT at all visits who had both genotype data that passed strict quality control measures and diabetes phenotype data were included.

***Genetic Epidemiology Network of Arteriopathy (GENOA)***

GENOA is one of the four networks in the Family Blood Pressure Program (FBPP) which recruited hypertensive black and non-Hispanic white sibships for linkage and family-based association studies to investigate genetic contributions to blood pressure in multiple racial groups [74]. Recruitment (Exam 1, 1995-2000 and Exam 2, 2000-2005) was population-based in two geographic locations: Jackson, Mississippi and Rochester, Minnesota. African Americans were recruited solely at the Jackson field center. Hypertensive probands were ascertained from the Jackson cohort of the Atherosclerosis Risk in Communities (ARIC) study if they were in a sibship with ≥ 2 individuals with essential hypertension (systolic blood pressure (BP) ≥140 mm Hg or diastolic BP ≥90 mm Hg on the second and third clinic visit), diagnosed prior to age 60, and consented to participate. Index sib-pairs with possible secondary hypertension, including sib-pairs with previously diagnosed kidney disease (defined by serum creatinine level > 2.5 mg/dl), were excluded. GENOA African Americans overlapping with participants in the ARIC study were excluded. In this study, 293 T2D participants and 246 NGT participants and who were self-reported African Americans were included.

***Healthy Aging in Neighborhoods of Diversity across the Life Span Study (HANDLS)***

The HANDLS study is an interdisciplinary, community-based, prospective longitudinal epidemiologic study examining the influences of race and socioeconomic status (SES) on the development of age-related health disparities among socioeconomically diverse African Americans and whites in Baltimore [75]. This study investigates whether health disparities develop or persist due to differences in SES, differences in race, or their interaction. This study is unique because it assesses over a 20-year period physical parameters as well as evaluate genetic, biologic, demographic, and psychosocial parameters of African American and white participants with higher and lower SES. The study domains include: nutrition, cognition, biologic biomarkers, body composition and bone quality, psychophysiology, physical function and performance, socio-demographics, psychosocial, neighborhood environment, and cardiovascular disease. Utilizing data from these study domains will facilitate understand the driving factors behind persistent black-white health disparities in overall longevity, cardiovascular disease, and cognitive decline. The mechanisms or biologic and molecular pathways through which the health and longevity trajectories of individuals in American society are influenced are unknown at this time.

The HANDLS design is an area probability sample of Baltimore based on the 2000 Census. The study protocol facilitated our ability to recruit 3,722 participants from Baltimore, MD with mean age 47.7 (range 30-64) years, of which 2,200 were African Americans (59%) and 1,522 were whites (41%), and 41% reported household incomes below the 125% poverty delimiter. The study is currently conducting wave 3 designed as a re-examination wave of all participants seen between years 2004-2009. In this study, 104 T2D cases diagnosed at any visit and 664 controls with NGT at all visits and who were self-reported African Americans were included.

***Health, Aging, and Body Composition (Health ABC) Study***

The Health ABC Study is a NIA-sponsored prospective cohort study to investigate the factors that contribute to incident disability and the decline in function of healthier older persons, with a particular emphasis on changes in body composition in old age [76]. Between April 1997 and June 1998, the study recruited 3,075 community-dwelling adults (41% African Americans) aged

70-79 years who were initially free of mobility and activities of daily living disability from the Pittsburgh, PA, and Memphis, TN, metropolitan areas. In this study, prevalent and incident cases of T2D were combined to constitute T2D cases. Prevalent T2D cases at the baseline clinic visit (1997-1998) were defined by self-reported MD diagnosis of diabetes or fasting plasma glucose ≥ 126 mg/dl or OGTT ≥ 200 mg/dl. Incident cases were identified at Year 2 (1998-1999), 4 (2000-2001), and 6 (2002-2003) using the same criteria applied to the baseline visit. Controls were defined as the followings: not self-reporting MD diagnosis of diabetes, and fasting plasma glucose < 100 mg/dl, and 2-hour OGTT glucose < 140 mg/dl at the baseline visit and at all available follow-up visits. In this study, 382 T2D cases and 232 NGT controls who were self-reported African Americans were included. For data analysis, the covariates age and BMI were selected at baseline for prevalent T2D cases, at the visit when the participant was first identified as a case for incident T2D cases, and at the last available visit when fasting plasma glucose and OGTT were measured for controls.

***Howard University Family Study (HUFS)***

The HUFS is a population-based study of African American families enrolled from the Washington, D.C. metropolitan area [77]. In the first phase of recruitment, a randomly ascertained cohort of 350 African American families with members in multiple generations from the Washington, D.C. metropolitan area were enrolled and examined. Families were not ascertained based on any phenotype. In a second phase of recruitment, additional unrelated and phenotypically unascertained individuals from the same geographic area were enrolled. In this study, 183 T2D cases and 738 NGT controls were included.

***Jackson Heart Study (JHS)***

The JHS is a prospective population-based study to examine the risk factors of cardiovascular disease, T2D, obesity, chronic kidney disease and stroke among African Americans in the Jackson, Mississippi metropolitan area [78]. A total of 5,301 self-identified African Americans were recruited from two cohorts of unrelated (aged 35-84 years) and nested family-based (aged ≥21 years) subjects during the baseline visit between years 2000-2004. In this study, 333 T2D and 1450 NGT subjects at baseline visit who were not enrolled in the ARIC study were included.

### *Multi-Ethnic Study of Atherosclerosis (MESA)*

The MESA is a study of the characteristics of subclinical cardiovascular disease (disease detected non-invasively before it has produced clinical signs and symptoms) and the risk factors that predict progression to clinically overt cardiovascular disease or progression of the subclinical disease [79]. MESA researchers study a diverse, population-based sample of 6,814 asymptomatic men and women aged 45-84 who were enrolled between years 2000-2002. Approximately 38% of the recruited participants are white, 28% African-American, 22% Hispanic, and 12% Asian, predominantly of Chinese descent. Participants were recruited from six field centers (Wake Forest School of Medicine, Columbia University, Johns Hopkins University, University of Minnesota, Northwestern University, and University of California - Los Angeles). Key phenotypes included coronary calcification, ventricular mass and function, flow-mediated endothelial vasodilation, carotid intimal-medial wall thickness and presence of echogenic lucencies in the carotid artery, lower extremity vascular insufficiency, arterial wave forms, electrocardiographic (ECG) measures, standard coronary risk factors, socio-demographic factors, lifestyle factors, and psychosocial factors. DNA has been extracted and lymphocytes cryopreserved. Participants have been contacted every 9 to 12 months throughout the study to assess clinical morbidity and mortality. Data from up to the fourth visit are available for analyses. In this study, 411 T2D subjects diagnosed at any visits and 793 subjects with NGT at all visits who were self-reported African Americans were included.

### *Multi-Ethnic Study of Atherosclerosis Family (MESA Family) Study*

The general goal of the MESA Family Study is to locate and identify genes contributing to the genetic risk for cardiovascular disease by looking at the early changes of atherosclerosis within families (mainly siblings) [80]. The MESA Family cohort was recruited from six MESA field centers. MESA Family participants underwent the same examination as MESA participants during May 2004 - May 2007. In a small proportion of subjects, parents of index subjects participating in MESA Family were studied but only to have blood drawn for genotyping. DNA was extracted and lymphocytes immortalized for 1,633 non-classic MESA family members (950 African Americans and 683 Hispanic-Americans) from 594 families, yielding 3,026 sibpairs. In this study, 69 T2D cases and 551 NGT controls who were self-reported African Americans were included.

***Sea Islands Genetic Network – Reasons for Geographic And Racial Differences in Stroke (SIGNET-REGARDS)***

The Sea Islands Genetics Network (SIGNET) study consists of REGARDS (Reasons for Geographic And Racial Differences in Stroke), Project SuGAR (Sea Islands Genetic African American Registry), the COBRE (Center of Biomedical Research Excellence) Oral Health pilot project, "An Epidemiological Study of Periodontal Disease and Diabetes: Cytokine Genes and Inflammation Factors", and SLEIGH (Systemic Lupus Erythematosus in Gullah Health study). All SIGNET samples (n= 4,298) were genotyped using the Affymetrix Genome-Wide Human SNP Array 6.0. Imputation was performed using MACH (version 1.0.16) to impute all autosomal SNPs using the CEU+YRI reference panel (as supplied by Goncalo Abecasis) from build 36 (2,318,207 SNPs in total). All subjects are African American (AA) and all provided written informed consent.

REGARDS is an observational cohort of 30,239 AA and white men and women enrolled in their homes after a telephone interview in 2003-2007 [81]. Participants were a national sample aged 45 years or older, oversampled from the southeastern stroke belt (56%) and were 58% female and 42% black by design. Participants were followed every 6 months by telephone to ascertain health outcomes, with validation of stroke, coronary heart disease, death and other ancillary study endpoints. Diabetes was defined as taking insulin or oral hypoglycemics, or either a fasting blood glucose $\geq$126 mg/dl or a nonfasting blood glucose $\geq$200 mg/dl. For SIGNET, we selected all AA REGARDS diabetes cases recruited from SC, GA, NC, and AL, and an equivalent number of race, sex, and age-strata matched diabetes-free controls. We also included all participants not already selected that were current residents of the 15-county "Low Country" region of SC and GA (SC counties Beaufort, Berkeley, Charleston, Colleton, Dorchester, Georgetown, Hampton, Horry, Jasper; GA counties Bryan, Camden, Chatham, Glynn, Liberty, McIntosh). Only the REGARDS sample from the SIGNET project was included in this investigation, and is referred to as SIGNET-REGARDS. GWAS genotyping was completed for 2,384 SIGNET-REGARDS participants used in this study, including 1,141 with diabetes and 1,243 without diabetes.

***Wake Forest School of Medicine (WFSM)***

The WFSM is a cross-sectional case-control study designed to examine the genetics of T2D and end-stage renal disease (ESRD) in African Americans [6,82]. In this study, the cases included 920 subjects with both T2D and ESRD recruited from dialysis facilities in the southeastern U.S. T2D patients were diagnosed after the age of 25 and did not receive only insulin therapy since diagnosis. In addition, cases had at least one of the following inclusion criteria: a) T2D diagnosed at least 5 years before initiating renal replacement therapy, b) background or greater diabetic retinopathy and/or c) ≥100 mg/dl proteinuria on urinalysis in the absence of other causes of nephropathy. The controls included 801 African American subjects without a current diagnosis of diabetes or renal disease recruited from the community and internal medicine clinics at WFSM. All subjects were recruited in North Carolina, South Carolina, Georgia, Tennessee, or Virginia. The FIND and WFSM samples were combined for association analysis.

***Women's Health Initiative – SNP Health Association Resource (WHI-SHARe)***

WHI is one of the largest (n=161,808) studies of women's health ever undertaken in the U.S. There are two major components of WHI: (1) a Clinical Trial (CT) that enrolled and randomized 68,132 women ages 50 – 79 into at least one of three placebo-control clinical trials (hormone therapy, dietary modification, and calcium/vitamin D); and (2) an Observational Study (OS) that enrolled 93,676 women of the same age range into a parallel prospective cohort study [83]. A diverse population including 26,045 (17%) women from minority groups were recruited from 1993-1998 at 40 clinical centers across the U.S. Of the CT and OS minority participants enrolled in WHI, 12,157 (including 8,515 self-identified African Americans and 3,642 self-identified Hispanic subjects) who had consented to genetic research were eligible for the WHI SHARe GWAS project. In this study, 1,964 T2D cases and 6,243 non-diabetic controls who were self-reported African Americans were included.

**Description of Stage 2a Replication Studies**

***Electronic Medical Records and Genomics Network (eMERGE)***

The Electronic Medical Records and Genomics (eMERGE) consortium formed in 2007 to leverage electronic medical record (EMR) linked DNA biorepositories can be leveraged for high-throughput genomic research [84]. It consists of five institutions (Group Health Cooperative,

Marshfield Clinic, Mayo Clinic, Northwestern University, and Vanderbilt University) each using an EMR for documentation of routine clinical care linked to a research specimen biorepository. The self-identified (NU) or third-party-identified (VU) African-American T2D cases and controls come from only the latter two sites. These institutions obtained appropriate approval from their respective Institutional Review Boards, and made use of a common Data Use Agreement to enable data sharing between institutions. Additional details have been previously published [84,85]. We used the existing clinical diagnostic criteria developed by the American Diabetes Association to develop a medical informatics algorithmic approach to identify 730 T2D patients as well as 830 control subjects without T2D using commonly captured EMR data, including diagnostic codes, medications, and laboratory test results. The algorithms are described elsewhere [86], and genotyping was performed at the Broad Institute on Illumina 1M Bead Chips [87].

***The Charles Bronfman Institute for Personalized Medicine (IPM) BioBank Program***

The IPM Biobank Program is a consented, EMR-linked medical care-setting biorepository of the Mount Sinai Medical Center (MSMC) drawing from a population of over 70,000 inpatients and 800,000 outpatient visits annually [88]. The MSMC serves diverse local communities of upper Manhattan, including Central Harlem (86% African Americans), East Harlem (88% Hispanic Latino), and Upper East Side (88% Caucasian/white) with broad health disparities. The IPM Biobank operations are fully integrated in clinical care processes, including direct recruitment from clinical sites waiting areas and phlebotomy stations by dedicated Biobank recruiters independent of clinical care providers, prior to or following a clinician standard of care visit. Recruitment currently occurs at a broad spectrum of over 30 clinical care sites. Case and control status was derived directly from participants' longitudinal EMR. Type 2 diabetes cases were defined as age $\geq 25$ and any of the followings: random glucose $\geq 200$ mg/dl ever, physician-entered diagnosis (at least 2 occurrences on 2 separate days) ever, or T2D medication (at least 2 occurrences on 2 separate days) ever. Controls were defined as age $\geq 25$ and not having T2D and all glucose $< 100$ mg/dl (if any). In this study, genotype and phenotype data for 1,617 cases and 2,163 NGT controls of African ancestry were available for analyses. Of these, 814 individuals (532 cases and 282 controls) were genotyped using the Affymetrix Genome-Wide Human SNP Array 6.0 array and 2,966 individuals (1,085 cases and 1,881 controls) were genotyped using the

Illumina HumanOmniExpressExome BeadChip. Case-control genome-wide association analyses were performed separately in the two subsets.

***The Insulin Resistance Atherosclerosis Study (IRAS)***

The Insulin Resistance Atherosclerosis Study (IRAS) was an epidemiologic cohort study designed to examine the relationship between insulin resistance and carotid atherosclerosis across a range of glucose tolerance [89]. Individuals of self-reported African-American ethnicity were recruited in Oakland and Los Angeles, CA. Recruitment was balanced across age and glucose tolerance status. In this study, 115 unrelated T2D cases and 164 controls at baseline visit from the IRAS were included.

***The Insulin Resistance Atherosclerosis Family Study (IRAS Family)***

The IRAS Family Study was a family study designed to examine the genetic and epidemiologic basis of glucose homeostasis traits and abdominal adiposity. Details of the IRAS Family Study are described elsewhere [90]. Briefly, self-reported African American pedigrees were recruited in Los Angeles, CA. Probands with large families were recruited from the initial non-family-based IRAS Study [89], which was modestly enriched for impaired glucose tolerance and T2D, as well as from the general community. In this study, 66 T2D cases and 513 controls from 42 families at baseline visit of the IRAS Family Study were included.

***Southern Community Cohort Study (SCCS)***

The SCCS is a prospective cohort study focusing on investigation of racial disparities in the risk of cancer and other chronic diseases. Approximately 86,000 men and women ages 40-79 from 12 southeastern U.S. states were enrolled between 2002 and 2009, with African Americans comprising two-thirds of the cohort. Participants completed a comprehensive, in-person, baseline interview covering various aspects of health conditions, and behavioral factors, including personal and family medical history, and other lifestyle factors. During this interview, participants were asked, "Has a doctor ever told you that you have had diabetes or high blood sugar?" Participants responding yes were asked follow-up questions regarding their age at first diagnosis and use (and names) of prescription medications taken to manage their diabetes. In current study, we only considered subjects who meet the following additional criteria as cases: 1)

on T2D medication, 2) age of diagnosis >30, 3) no cancer at study enrollment, 4) self-reported AAs. Subjects responded ‘no’ to the question "Has a doctor ever told you that you have had diabetes or high blood sugar?" and had1) no cancer at study enrollment and 2) self-reported AAs were considered as potentially eligible controls. Controls were frequency-matched to cases (1:1) and by age at enrollment (within 5 years) to age of T2D onset, gender, and residence (same state). A total of 1,130 T2D cases and 1,130 controls were included in this study. Study participants provided written informed consent, and the study was approved by committees for the use of human subjects at both collaborating institutions.

***Wake Forest School of Medicine (WFSM)***

The WFSM replication cohort recruited African American T2D cases with ESRD and controls using the same criteria as the case and control subjects that were used in the GWAS study [6,82]. T2D cases without evidence of nephropathy were also recruited from medical clinics, churches, health fairs and community resources at the same geographic areas. In this study, 2,403 T2D cases (1,256 cases with both T2D and ESRD, and 1,147 cases with T2D) and 683 controls were included.

**References**


66. The ARIC Investigators (1989) The Atherosclerosis Risk in Communities (ARIC) Study: design and objectives. The ARIC investigators. Am J Epidemiol 129: 687-702.
67. Musunuru K, Lettre G, Young T, Farlow DN, Pirruccello JP, et al. (2010) Candidate gene association resource (CARe): design, methods, and proof of concept. Circ Cardiovasc Genet 3: 267-275.
68. Friedman GD, Cutter GR, Donahue RP, Hughes GH, Hulley SB, et al. (1988) CARDIA: study design, recruitment, and some characteristics of the examined subjects. J Clin Epidemiol 41: 1105-1116.
69. Redline S, Tishler PV, Tosteson TD, Williamson J, Kump K, et al. (1995) The familial aggregation of obstructive sleep apnea. Am J Respir Crit Care Med 151: 682-687.
70. Fried LP, Borhani NO, Enright P, Furberg CD, Gardin JM, et al. (1991) The Cardiovascular Health Study: design and rationale. Ann Epidemiol 1: 263-276.
71. Higgins M, Province M, Heiss G, Eckfeldt J, Ellison RC, et al. (1996) NHLBI Family Heart Study: objectives and design. Am J Epidemiol 143: 1219-1228.
72. Knowler WC, Coresh J, Elston RC, Freedman BI, Iyengar SK, et al. (2005) The Family Investigation of Nephropathy and Diabetes (FIND): design and methods. Journal of Diabetes and Its Complications 19: 1-9.
73. Kral BG, Mathias RA, Suktitipat B, Ruczinski I, Vaidya D, et al. (2011) A common variant in the *CDKN2B* gene on chromosome 9p21 protects against coronary artery disease in Americans of African ancestry. J Hum Genet 56: 224-229.
74. Daniels PR, Kardia SL, Hanis CL, Brown CA, Hutchinson R, et al. (2004) Familial aggregation of hypertension treatment and control in the Genetic Epidemiology Network of Arteriopathy (GENOA) study. Am J Med 116: 676-681.
75. Evans MK, Lepkowski JM, Powe NR, LaVeist T, Kuczmarski MF, et al. (2010) Healthy aging in neighborhoods of diversity across the life span (HANDLS): overcoming barriers to implementing a longitudinal, epidemiologic, urban study of health, race, and socioeconomic status. Ethn Dis 20: 267-275.
76. Harris TB, Visser M, Everhart J, Cauley J, Tylavsky F, et al. (2000) Waist circumference and sagittal diameter reflect total body fat better than visceral fat in older men and women. The Health, Aging and Body Composition Study. Ann N Y Acad Sci 904: 462-473.
77. Adeyemo A, Gerry N, Chen G, Herbert A, Doumatey A, et al. (2009) A genome-wide association study of hypertension and blood pressure in African Americans. PLoS Genet 5: e1000564.
78. Taylor HA, Jr., Wilson JG, Jones DW, Sarpong DF, Srinivasan A, et al. (2005) Toward resolution of cardiovascular health disparities in African Americans: design and methods of the Jackson Heart Study. Ethn Dis 15: S6-4-17.
79. Bild DE, Bluemke DA, Burke GL, Detrano R, Diez Roux AV, et al. (2002) Multi-ethnic study of atherosclerosis: objectives and design. Am J Epidemiol 156: 871-881.
80. Manichaikul A, Chen WM, Williams K, Wong Q, Sale MM, et al. (2011) Analysis of family- and population-based samples in cohort genome-wide association studies. Hum Genet.
81. Howard VJ, Cushman M, Pulley L, Gomez CR, Go RC, et al. (2005) The reasons for geographic and racial differences in stroke study: objectives and design. Neuroepidemiology 25: 135-143.

82. McDonough CW, Palmer ND, Hicks PJ, Roh BH, An SS, et al. (2011) A genome-wide association study for diabetic nephropathy genes in African Americans. Kidney Int 79: 563-572.
83. Women's Health Initiative (1998) Design of the Women's Health Initiative clinical trial and observational study. The Women's Health Initiative Study Group. Control Clin Trials 19: 61-109.
84. McCarty CA, Chisholm RL, Chute CG, Kullo IJ, Jarvik GP, et al. (2011) The eMERGE Network: a consortium of biorepositories linked to electronic medical records data for conducting genomic studies. BMC Med Genomics 4: 13.
85. Kho AN, Pacheco JA, Peissig PL, Rasmussen L, Newton KM, et al. (2011) Electronic medical records for genetic research: results of the eMERGE consortium. Sci Transl Med 3: 79re71.
86. Kho AN, Hayes MG, Rasmussen-Torvik L, Pacheco JA, Thompson WK, et al. (2012) Use of diverse electronic medical record systems to identify genetic risk for type 2 diabetes within a genome-wide association study. J Am Med Inform Assoc 19: 212-218.
87. Turner S, Armstrong LL, Bradford Y, Carlson CS, Crawford DC, et al. (2011) Quality control procedures for genome-wide association studies. Curr Protoc Hum Genet Chapter 1: Unit1 19.
88. Tayo BO, Teil M, Tong L, Qin H, Khitrov G, et al. (2011) Genetic background of patients from a university medical center in Manhattan: implications for personalized medicine. PLoS ONE 6: e19166.
89. Wagenknecht LE, Mayer EJ, Rewers M, Haffner S, Selby J, et al. (1995) The insulin resistance atherosclerosis study (IRAS) objectives, design, and recruitment results. Ann Epidemiol 5: 464-472.
90. Henkin L, Bergman RN, Bowden DW, Ellsworth DL, Haffner SM, et al. (2003) Genetic epidemiology of insulin resistance and visceral adiposity. The IRAS Family Study design and methods. Ann Epidemiol 13: 211-217.
